# Acknowledgment to Reviewers of *Sensors* in 2021

**DOI:** 10.3390/s22031052

**Published:** 2022-01-29

**Authors:** Sensors Editorial Office

**Affiliations:** MDPI AG, St. Alban-Anlage 66, 4052 Basel, Switzerland

Rigorous peer-reviews are the basis of high-quality academic publishing. Thanks to the great efforts of our reviewers, *Sensors* was able to maintain its standards for the high quality of its published papers. Thanks to the contribution of our reviewers, in 2021, the median time to first decision was 16 days and the median time to publication was 40 days. The editors would like to extend their gratitude and recognition to the following reviewers for their precious time and dedication, regardless of whether the papers they reviewed were finally published:
A. D. PrasadKean C. AwA. Dan WilsonKechen SongAakanksha TewariKee S. MoonAarón Ángel Salas-SánchezKeekeun LeeAaron J. PungKefeng GuoAashish PriyeKefeng JiAbas SabouniKegang ZhaoAbayomi OtebolakuKei Hang Katie ChanAbbas Abbaszadeh ShahriKei MasaniAbbas AcarKei NagashimaAbbas HaddadiKei NakagawaAbbas MadaniKeiichi KitajoAbbas OrandKeiji NagaiAbbas ShafieeKeiko OgawaAbbas ShahriKeisuke OtsukaAbbass NasserKeith GoossenAbd Krim SeghouaneKeji YangAbdallah MoubayedKele XuAbdel F. IsakovicKelly M. SullivanAbdel Fattah ShetaKelly SheerinAbdeldjalil OuahabiKelm KlemensAbdelfateh KerroucheKelvin Ian AfrashtehfarAbdelhak MerizigKelvin WongAbdelhameed FawzyKemal SümserAbdelhamied AshrafKen KiyonoAbdelkader Nasreddine BelkacemKen MasamuneAbdelkrim RebiaiKen PfeuferAbdellah ChehriKen SasakiAbdellatif AkjoujKen WatanabeAbdellatif ZaidiKeng-Hao LiuAbdelmalek BouguettayaKenichiro TodorokiAbdelrhman MohamedKenji AonoAbdelwahab BoualouacheKenji KawashimaAbdollah AhmadiKenji ShimazoeAbdon AtanganaKenneth A. LoparoAbdu GumaeiKenneth LohAbdul MajeedKenneth M. WachaAbdul Rahim FerhanKenneth ScheplerAbdul Rashid AzizKenneth SutherlandAbdul Rashid ShariffKenny DavilaAbdulhamit SubasiKenric NelsonAbdullah Aamir HayatKenshi SahoAbdullah AbuolaimKensuke OgawaAbdullah AlharthiKenta IitaniAbdullah AlomariKenta MatsumuraAbdullah BayramKenta ObataAbdullah LakhanKentaroh ToyodaAbdullah WaqasKeonwook KimAbdulrahman Khalaf Al-AliKeren DaiAbdul-Sattar KaddourKerry PeekAbebe Belay AdegeKerry SunAbebe DiroKertész IstvánAbel Cabrera MartínezKeshav SinghAbel Yeboah-OforiKesheng WangAbhay KotnalaKetan KotechaAbhijeet RavankarKetson Roberto Maximiano Dos SantosAbhijit GhoshKeunhwa LeeAbhijit SarkarKeval VoraAbhik BanerjeeKévin BouchardAbhilash PandyaKe-Vin ChangAbhimanyu Singh GarhwalKevin DesaiAbhimanyu ThakurKevin F. BronsonAbhishek GhoshKevin HuangAbhishek KaushikKevin M. DanielsAbhishek KumarKevin MurphyAbhishek Kumar JhaKevin NickelsAbhishek KunduKevin StaatsAbhishek SharmaKevin TangAbhishek TiwariKeyan WangAbhishek VenketeswaranKeyvan JaferzadehAbhrajit SenguptaKezheng LiAbid KhanKh Tohidul IslamAbid NadeemKhader HamdiaAbiel Aguilar-GonzálezKhader ShameerAbraham Boby RibyKhadour AghiadAbu Sadat Md SayemKhaldoun AlkhalifehAbu Saleh Md BakibillahKhaled ArdahAbu Sayed ChowdhuryKhaled GiasinAbu Ul Hassan Sarwar RanaKhaled ShuaibAbubakar SharafatKhalfalla AwedatAchilleas SamarasKhalid ElgazzarAchim BittnerKhalid SaeedAchim HabekostKhalid SayoodAchraf AmmarKhalil RamadiAda FortKhan MuhammadAdam Adam GlowaczKhan Rana Muhammad AsadÁdám BárdosKhawaja Khalid MehmoodAdam BrysiewiczKhayyam MasoodAdam BujnowskiKhin ThaAdam ChromyKhristine Joy C. AntiguaAdam DomańskiKhurram HameedAdam GąskaKhushboo MunirAdam GlowaczKi H. ChonAdam HenschkeKi Il KimAdam HorinKi Soo ParkAdam KawalecKi Yong OhAdam KiersztynKia DashtipourAdam KlimowiczKiang Jean-FuAdam KłodowskiKi-Baek LeeAdam KolińskiKichun LeeAdam KoniuszyKien NguyenAdam Krzysztof PilatKien Voon KongAdam L. KaczmarekKieran MoranAdam M. WojciechowskiKifayat UllahAdam MaleyKihan ParkAdam MarchewkaKiho LimAdam MartowiczKi-Ho ParkAdam MilikKihwan KimÁdám NyergesKiichi NiitsuAdam ReevesKi-Il KimAdam RinglerKijin KimAdam RogowskiKijoon LeeAdam RosińskiKil ChongAdam RutkowskiKim Fung TsangAdam SantorelliKim Jee-InAdam WisniewskiKiman KimAdam WojciechowskiKimerly PowellAdam ZagubieńKimihiro SakagamiAdam ZającKimin YunAdam ZiębińskiKimmo KeränenAdamantia StamouKin Hing LoAdel AltiKi-Nam JooAdel FathyKinan GhanemAdela BadauKing Fai HungAdelaida AvinoKing Man SiuAdelaide MirandaKin-hong WongAdele SaterianoKinjiro AmanoAdewumi John BabafemiKira V. VyatkinaAdi S. Abu-ObeidahKiril AlexievAdilson BerveglieriKirill TomyshevAdithya BalasubramanyamKishor Datta GuptaAditya KarnikKishu RanjanAditya P. MathurKi-Sun LeeAdmir MulahusićKittisak JermsittiparsertAdnan Ahmad CheemaKiwon MoonAdnan MahmoodKiyotaka FujisakiAdnan MujezinovićKiyoung KimAdnan Ul HaqueKjartan HalvorsenAdnane NoualKlaas DijkstraAdolfo Ruiz-CallejaKlaas PaulyAdolfo SantoroKlara MegyeriAdrian BarbuKlas IhmeAdrian BekasiewiczKlaske Van KammenAdrian BurlacuKlaudia SmolągAdrian Elmi-TeranderKlaus CocoAdrián Fernández GavelaKlaus KorenAdrian FilipescuKlaus MathwigAdrian GligorKlaus Michael SpohrAdrián González-SieiraKlaus SchäferAdrian KapczyńskiKlaus SzielaskoAdrian KeatingKlaus ThoeniAdrian KliksKlaus Werner StöckelhuberAdrian KorodiKleftodimos AlexandrosAdrian Martinez-RivasKlemen BučarAdrian OlaruKlemen KregarAdrian PeidroKleomenis KalogeropoulosAdrian S. BarbKnut ØvsthusAdrian Sergiu DarabantKogularasu SakthivelAdrian StercaKoichi KuritaAdrian SternKoichi MaezawaAdrian TamayoKoichiro MiyamotoAdrian WilsonKoichiro ShiomoriAdrian WlodarczakKoji HashiguchiAdriana AlexandruKoji InoueAdriana DapenaKongzhe ChenAdriana FerlazzoKonlin ShenAdriana Peña Pérez NegronKonrad Jerzy KapciaAdriana PuiuKonrad KielbasinskiAdriana Vargas-NordcbeckKonrad MollenhauerAdrian-Ioan PetrariuKonrad RudnickiAdrian-Mihail StoicaKonrad WaluśAdriano AndradeKonstantin B. YushkovAdriano ColaKonstantin E. SamouylovAdriano MoreiraKonstantin GugletaAdriano SantosKonstantin KozlovAdriel AraújoKonstantin MarkovAdrien UgonKonstantin MikhaylovAdrienn DinevaKonstantin MikhelsonAdroaldo RaizerKonstantin NeusypinAfaque ManzoorKonstantin NikolaevAfef FekihKonstantin P. AndryushinAgapi MesodiakakiKonstantin P. GaikovichAgárdi AnitaKonstantin RumyantsevAgata GabryelskaKonstantinos A. BanitsasAgata GiełczykKonstantinos C. ZikidisAgata KociaKonstantinos DemertzisAgata KołakowskaKonstantinos GiannakisAgata StanekKonstantinos KarampidisAgata Szłapa-KulaKonstantinos KoliasAgathoklis GiaralisKonstantinos KotisAgeliki KaratzaKonstantinos LimniotisAggeliki SgoraKonstantinos M. GiannoutakisAggelos PallikarakisKonstantinos MakantasisAgne Paulauskaite-TarasevicieneKonstantinos MaliatsosAgnelo R. SilvaKonstantinos MisiakosAgnes PurwidyantriKonstantinos NikolakopoulosÁgnes Vathy-FogarassyKonstantinos TountasAgnese SbrolliniKonstantinos TsongasAgnieszka AdamczukKonstantins JefimovsAgnieszka BarczakKoojana KuladinithiAgnieszka BitkowskaKooktae LeeAgnieszka BlokusKoorosh GharehbaghiAgnieszka ChoroszuchoKörstgens VolkerAgnieszka Dołhańczuk-ŚródkaKosai RaoofAgnieszka DudziakKosmas AlexopoulosAgnieszka FiszerKosmas DimitropoulosAgnieszka Gadomska-GajadhurKosmas KavadiasAgnieszka Kącka-ZychKostas KolomvatsosAgnieszka KonysKostas PeppasAgnieszka KrólickaKostas YiannopoulosAgnieszka LazarowskaKota KodamaAgnieszka Łupicka-SłowikKothandam GopalakrishnanAgnieszka MlynarskaKouichi SakuraiAgnieszka PękalaKourosh ZareiniaAgnieszka StolarczykKovacic BostjanAgnieszka SzczęsnaKranthi K. ManiamAgnieszka Szmelter-JaroszKrassimira VlachkovaAgnieszka SzypłowskaKrešimir TrontlAgnieszka TercjakKrishanu RoyAgnieszka TomaszewskaKrishna P. KadiyalaAgnieszka WareńczakKrishna PersaudAgnieszka WięckowskaKrishnamurthy VemuruAgnieszka WosiakKrishnan MurugappanAgostino CortesiKristel FobeletsAgostino ForestieroKristen M. MeiburgerAgostino IadiciccoKristi SantiAgostino OcchiconeKristian DokicAguinaldo FraddosioKristian KostalAgustin L. Herrera-MayKristian MathisAgustin Zaballos DiegoKristijan LenacAgustın-Agüera PérezKristina DaunoravicieneAhad BehboodiKristina L. Allen-BradyAh-Lian KorKristína MachováAhmad AliKristina StojmenovaAhmad Al-KhasawnehKristina ŠutienėAhmad BazziKristine KrajnakAhmad FarooqKristoffer PeterssonAhmad HassanKrystian ErwińskiAhmad JahanbakhshiKrystian JobczykAhmad JalalKrystian KoziołAhmad JbaraKrystian MistewiczAhmad MohsinKrystian PykaAhmad MostafaKrystian RadlakAhmad P. TaftiKrystyna JabłońskaAhmad SulimanKrzysztof B. BećAhmad T. AlmutawaKrzysztof BarteckiAhmad Taher AzarKrzysztof BlecharzAhmed A. IbrahimKrzysztof BrzostowskiAhmed AbassKrzysztof ChwastekAhmed AbdelazizKrzysztof CzaplewskiAhmed Abdelmottaleb OmarKrzysztof DołowyAhmed AbouhussienKrzysztof GaskaAhmed AljaafKrzysztof GóreckiAhmed DorrahKrzysztof GórnickiAhmed El OualkadiKrzysztof GrochlaAhmed El-AwamryKrzysztof JamroziakAhmed ElhattabKrzysztof K. DudekAhmed ElnakibKrzysztof KarwowskiAhmed ElSaidKrzysztof KlimaszewskiAhmed ElSayedKrzysztof KluzaAhmed IsmailKrzysztof KukielkaAhmed JalalKrzysztof KulpaAhmed KhaledKrzysztof MałeckiAhmed Mohamed Fahmy YousefKrzysztof MusiołAhmed MohammedKrzysztof NausAhmed RachidKrzysztof NykaAhmed RadyKrzysztof OkarmaAhmed Ramadhan Al-ObaidiKrzysztof PielichowskiAhmed Shaharyar KhwajaKrzysztof PieńkowskiAhmed ZahranKrzysztof PrzybyłAhmed Zainul AbideenKrzysztof PrzystupaAhmed ZohaKrzysztof Psiuk-MaksymowiczAhmet Can SabuncuKrzysztof R. KarszniaAhmet Fatih TabakKrzysztof RechowiczAhmet ZengïnKrzysztof RogowskiAhyoung LeeKrzysztof RyczkoAibin YanKrzysztof RzeckiAi-Chun PangKrzysztof SawickiAida AzadeganKrzysztof SchabowiczAiert Amundarain IrizarKrzysztof SkabekAigars AtvarsKrzysztof SkrzypkowskiAiguo SongKrzysztof SośnicaAi-hong JiKrzysztof StępieńAijun ZhuKrzysztof SzabatAilan CheKrzysztof TadyszakAileni Raluca MariaKrzysztof TargielAimé Lay-EkuakilleKrzysztof TomczykAimée MearsKrzysztof WajdaAimin ChangKrzysztof WalasAimin JiangKrzysztof WałęsaAimin YangKrzysztof WesolowskiAirat SakhabutdinovKsenia OstrowskaAirat Zh. SakhabutdinovKshitij SharmaAishwaryadev BanerjeeKuan SunAisling O’DriscollKuang ShengAitor AlmeidaKuang-Hao LinAitor ArriolaKuan-Hui LeeAitor Oyarbide-ZubillagaKuanyu ChenAjay AshokKuen-Suan ChenAjay K. VermaKui XuAjay KevatKumar Biswajit DebnathAjay PalKumar PrashantAjay Vikram SinghKumar YelamarthiAjeet KaushikKun JiaAjit AhlawatKun Lin LeeAjit JhaKun LiuAjith PattammattelKun QianAjmal KhanKun XuAkanksha BhutaniKun ZhangAkbar A. KhatibiKun-Chih ChenAkhil KandhariKun-Hui ChenAkhil VaidKuniaki NagamineAkhila VeerubhotlaKunjie ChenAkhilesh S. P. KhopeKun-mean HouAkhilesh ThyagaturuKunpeng FengAkhtar HussainKuo-Chang LuAkihiro NakataniKuo-Cheng HuangAkilu Yunusa-KaltungoKuo-Chih ChuangAkinori IrizawaKuo-Hu ChenAkio TsunedaKuo-Jen ChangAkira IkutaKuo-Kun TsengAkitoshi ShiotariKuo-Liang ChungAkkapol Suea-NgamKuo-Liang HuangAkm Azad HossainKuo-Lung LianAkos KereszturiKuo-Lung WangÁkos KukoveczKuo-Ping LinÁkos OdryKuo-Yi LinAkrem SellamiKurt AmmerAku VisuriKurt DebattistaAla KhalifehKurt MalyAla MoradianKurt W. KolasinskiAlaa Abdulhady JaberKutubuddin AnsariAlaa AlZoubiKwan Kyu ParkAlaa Awad AbdellatifKwang LeeAlaelddin Fuad Yousif MohammedKwang Ryul BaekAlain GeigerKwang-Hoon KimAlain TremeauKwangseok OhAlan CalheirosKwok Ping ChanAlan DavoustKwok Tai ChuiAlan Demetrius Baria ValejoKwok-Fan ChowAlan J. MichaelsKwo-Ting FangAlan McGibneyKyandoghere KyamakyaAlan MeierKyeong-Keun ChoiAlan T. LitchfieldKyle NelsonAlanoud SubahiKyle R. WiltAlban GoupilKyo InoueAlban KuriqiKyong Hon KimAlbane SaintenoyKyoung Youl ParkAlbena AntonovaKyriaki KostoglouAlberico SonnessaKyu Tae ParkAlbert ArgilagaKyu-haeng LeeAlbert De VriesKyujung KimAlbert LeeKyuman LeeAlbert ModiKyung Chul OhAlbert RogersKyung Hyun ChoiAlbert SabbanKyung Wha OhAlbert SolernouKyungeun SungAlbert VetteKyungha MinAlberto BarontiniKyungHi ChangAlberto CalatroniKyung-Joon ParkAlberto CardosoKyungkwang JooAlberto Comesaña-CamposLadislav BeranekAlberto Cordoba IzaguirreLadislav FőzőAlberto CoriglianoLadislav MarišAlberto DoriaLadislav PolakAlberto Eloy García GutiérrezLaeticia PetitAlberto FernandezLaia SubiratsAlberto Fernández-IsabelLaibing JiaAlberto FerranteLai-Kwan ChauAlberto GallegosLaio O. SemanAlberto Gómez-CaballeroLaio Oriel SemanAlberto GregoriLaís Canniatti BrazacaAlberto Izquierdo-FuenteLaith AlzubaidiAlberto Jorge Rosales SilvaLajos DarócziAlberto López-MartínezLal Mohan BhowmikAlberto Luviano-JuárezLaleh AvazpourAlberto MartiniLaleh BadriaslAlberto Miguel Bonastre PinaLalitha Madhavi Konila SriramAlberto MuscioLambros EkonomouAlberto OchoaLamiaa ElrefaeiAlberto Ochoa-ZezzattiLan XiangAlberto OlmoLan ZhangAlberto OrtizLana RuzicAlberto Pedrouzo-UlloaLance ChampagneAlberto PellegrinelliLang XuAlberto ReattiLanto RasolofondraibeAlberto Reyna MaldonadoLapo MiccinesiAlberto Rico-YusteLara A. ThompsonAlberto Rodrigue MartinezLara Del ValAlberto Rodríguez-ArchillaLara MottaAlberto RoncagliaLarisa DunaiAlberto Sánchez-AlzolaLarisa LvovaAlberto Tellaeche IglesiasLarissa Pereira Ribeiro TeodoroAlberto VallanLars Ohlsson FhagerAlberto VillalongaLarysa NeduzhaAlcenísio J. Jesus-SilvaLas KourisAldo GhisiLasitha PiyathilakaAldo GordilloLaszlo B. KishAldo JesorkaLászló BeneAldo MinardoLaszlo CsirmazAldo QuattroneLászló Csurgai-HorváthAldobenedetto ZottiLászló DemkóAleixandre ManuelLászló JuhászAlejandro Alvarado-LassmanLászló KovácsAlejandro BustosLászló SiposAlejandro CarrascoLatif Ullah KhanAlejandro Castañeda-MirandaLaura Alejandra Martínez-TejadaAlejandro Castillo AtocheLaura AntonelliAlejandro Criado FernándezLaura BragonzoniAlejandro DiazLaura BurattiniAlejandro García-Miranda FerrariLaura D’AlfonsoAlejandro Gómez-AlanísLaura DaniusevičiuteAlejandro JavaloyesLaura DossiAlejandro Martinez-RiosLaura FabbianoAlejandro Molina ZarcaLaura FalaschettiAlejandro Ramirez-RojasLaura FarinaAlejandro RodriguezLaura GastaldiAlejandro Roman LoeraLaura GiarréAlejandro Santos Martínez-SalaLaura I. Garay-JimenezAlejandro Vasquez-RifoLaura JasińskaAlejandro VegaLaura M. RoaAleksandar DimitrijevicLaura McnamaraAleksandar MaksimovicLaura MihaiAleksandar MešićLaura MorettiAleksandar MilosavljevićLaura PeraltaAleksandar ObradovicLaura PierucciAleksandar VakanskiLaura PirovanoAleksandar-Saša MilakovićLaura RomeoAleksander KrólLaura RuseAleksander LisowskiLaura StefaniAleksander MendykLaura TarantinoAleksander SešekLaura TolesAleksander SniadyLaura VerdeAleksander V. MarusinLaura-Maria DogariuAleksander WosniokLauren BensonAleksander ZidanšekLaurence De ClippeleAleksandr KlimovLaurence JolivetAleksandr Leonidovich KazakovLaurent BaboutAleksandr OreshonkovLaurent BarthesAleksandr RakhmangulovLaurent BeaudoinAleksandr RomanovLaurent SegersAleksandr SakhnevychLaurent SpinelleAleksandra Kawala-SterniukLaurentiu BaschirAleksandra KrampikowskaLauro OjedaAleksandra KristoLawrence R. RobinsonAleksandra KrólikowskaLazar BerbakovAleksandra RadulovićLázaro Eduardo Da SilvaAleksandra ŚwierczyńskaLazaros ArestiAleksandra SzydłowskaLazo M. ManojlovićAleksei TepljakovLazzaro Di BiaseAleksei UdovichenkoLe SunAleksejs ZacepinsLe Van MinhAleksey Anatolievich ZakharenkoLe YaoAleksey ErmoshkinLeandro Buss BeckerAleksey Yurevich KokhanovskiyLeandro MaioAleksy KwilinskiLefteris BenosAlena Novak SedlackovaLehel CsatóAleš FidlerLei FanAleš HaceLei GaoAleš JanotaLei GuoAles ProchazkaLei HanAleš SlívaLei HuangAleš VysockýLei JingAlessandra AnzolinLei LiAlessandra BiancolilloLei MaoAlessandra BorghiLei MeiAlessandra CapolupoLei QiuAlessandra FilippiLei ShiAlessandra LuccheseLei WanAlessandra RizzardiLei WangAlessandra Scotto di FrecaLei WuAlessandra SorrentinoLei XueAlessandra VitanzaLei YuAlessandra ZanutLei ZhaiAlessandro AldiniLei ZhangAlessandro BazziLei ZhouAlessandro BurgioLei ZhuAlessandro CapraLeijiao GeAlessandro CasavolaLeila KeshavarzAlessandro CataniaLeila Merghem BoulahiaAlessandro CelestiniLeilei LiAlessandro CerutiLei-Ming MaAlessandro CicolinLenka LhotskaAlessandro CidronaliLenny Van Erp-van Der KooijAlessandro CilardoLeo K. ChengAlessandro CocliteLéo MartireAlessandro CultreraLeobardo HernandezAlessandro Di BenedettoLeocundo Aguilar NoriegaAlessandro Di MarcoLeon ReznikAlessandro Enrico Cesar RedondiLeon RothkrantzAlessandro FantiLeon StefanijaAlessandro FedeliLeonard DausAlessandro FilippeschiLeonard FelicettiAlessandro Fraleoni-MorgeraLeonard HanssenAlessandro FrancoLeonard StoicaAlessandro GabrielliLeonardo Acho ZuppaAlessandro GaldelliLeonardo AguayoAlessandro Giuseppe D’AloiaLeonardo Alexandre Peyré-TartarugaAlessandro GrecoLeonardo AngeliniAlessandro LabellaLeonardo CiaccheriAlessandro Maria SelvitellaLeonardo D’AcquistoAlessandro MarroniLeonardo FrancoAlessandro MasulloLeonardo Garcia-GarciaAlessandro MeduriLeonardo HonórioAlessandro MengarelliLeonardo Juan Ramirez LopezAlessandro MingottiLeonardo Nascimento FerreiraAlessandro MorelliLeonardo PantoliAlessandro MoroLeonardo RundoAlessandro OrtisLeonardo VicarelliAlessandro Paolo DagaLeoneed M. KirilovAlessandro PozzebonLeonel Jorge Ribeiro NunesAlessandro PuiattiLeonel Paredes-MadridAlessandro RasuloLeong Chuan KwekAlessandro RidolfiLeonid BolotovAlessandro ScanoLeonid FetisovAlessandro ScordoLeonid SatrapinskyyAlessandro SeverinoLeonid V. KatkovskyAlessandro SopegnoLeonidas KourisAlessandro StefanoLeopoldo Acosta SanchezAlessandro TerenziLeopoldo Altamirano RoblesAlessandro TestaLesley GibsonAlessandro TonacciLeslie Ching Ow TiongAlessandro TorresaniLeslie WongAlessandro VizzarriLesya AnishchenkoAlessandro ZampognaLeszek AmbroziakAlessandro ZonaLeszek LuchowskiAlessio AboudanLeszek NowosielskiAlessio BastiLetitia MireaAlessio BruttiLev A. MatveevAlessio BuzzinLev JakubAlessio CascardiLev KuzminAlessio De AngelisLev T. PerelmanAlessio FascistaLevente CzumbilAlessio MartinelliLevente KleinAlessio PignalberiLevente TamásAlessio RicciLewei TangAlessio RossiLewis NkenyereyeAletha GómezLeyuan LiuAlex Borges VieiraLi FuAlex ButeanLi GangAlex BystrovLi KunAlex Fan XuLi LiAlex FragosoLi LinAlex FuerbachLi LiuAlex J. YuffaLi SunAlex MouapiLi TanAlex Sandro Roschildt PintoLi YangAlex ScottLi ZhaoAlex WeddellLi ZhengAlexander Aleksandrovich SafonovLia E AciuAlexander Alexandrovich GromovLian Yu ZhengAlexander BergmannLiana StanescuAlexander ChizhikLianbo MaAlexander DeistingLiandong LiAlexander FedotovLianfu HanAlexander FerwornLiang ChenAlexander GillersonLi-Ang LeeAlexander HunoldLiang LiangAlexander HvatovLiang XiaoAlexander IvannikovLiang XieAlexander KalashnikovLiang ZhaoAlexander KocianLiang ZouAlexander KölpinLiang-Bi ChenAlexander M. SamoylovLianggui LiuAlexander MorchenkoLiang-Hung WangAlexander Nesterov-MüllerLiangjiang YuAlexander OmelyanchikLiangjing YangAlexander PalmanshoferLiangliang ChengAlexander RobitzschLiangliang YangAlexander RouchLiangtian WanAlexander RyzhovLiangxing HuAlexander S. MachikhinLianjie ZhouAlexander SboevLianming LiAlexander SchaumLiansheng WangAlexander SchulzLianwu GuanAlexander SutinLianyong QiAlexander SutorLibin HuangAlexander TarasovLibin YeAlexander TiniusLibo GaoAlexander V. MantzarisLibo MaAlexander VelmuzhovLibo MengAlexander VolyarLibo ZhaoAlexander WöhrerLibor HargasAlexander YakuninLibor PekarAlexander Yu MeigalLibor VášaAlexandra BousiaLichota PiotrAlexandra DuminilLi-Chung WuAlexandra GemitziLidia FioriniAlexandra Kämpfer-HomsyLidia FotiaAlexandre BrandãoLidia OgielaAlexandre Campeau-LecoursLidia Sánchez GonzálezAlexandre Fieno Da SilvaLidong YangAlexandre Fonseca BrandãoLieselotte CortenAlexandre G. EvsukoffLieva Van LangenhoveAlexandre LocquetLifei WangAlexandre M. P. BotasLi-Fong LinAlexandre MeyerLifton Joseph JohnAlexandre NassifLiguo SunAlexandre PereiraLihai ZhangAlexandre RobichaudLiHaojun LiAlexandre SavaLihong DuanAlexandre SerresLihua LiAlexandre Vervisch-PicoisLihua MaAlexandros El SachatLihua WangAlexandros KarakostisLihui FengAlexandros PitilakisLihui LuoAlexandros StergiouLijun SunAlexandru ArchipLijun ZhangAlexandru BarsanLijun ZhaoAlexandru GheteLili WanAlexandru IsarLili XingAlexandru LavricLilian SosaAlexandru MartianLilian WitthauerAlexandru OneaLiliana Fadul-PachecoAlexandru PîrjanLiliana PorojanAlexandru SoriciLiliana Szyszka-SommerfeldAlexandru TakacsLiliana Vale CostaAlexandru VulpeLilly LiAlexandru-Nicolae TudosieLiming FangAlexandu VulpeLiming NieAlexei DmitrievLiming WangAlexei KamshilinLin CaoAlexei NabokLin ChenAlexey BeskopylnyLin FengAlexey BuzmakovLin LinAlexey FominLin MengAlexey G. VoloboyLin QiAlexey IvanovLin ShuAlexey KashevnikLin WangAlexey KureevLin WuAlexey S. KononikhinLin ZhangAlexey ShitvovLina GarcésAlexey ShutovLina LundgrenAlexey V. PovolotskiyLina MohjaziAlexey Vorob’evLina ZouAlexey WolfLinas PetkevičiusAlexios MylonasLinas SvilainisAlexios P. DouvalisLinbo ShaoAlexios PapacharalampopoulosLinchao LiAlexis MendezLincoln SilvaAlfonso Ariza QuintanaLinda M. EerikäinenAlfonso Gómez-EspinosaLinda PaternÒAlfonso González-BrionesLinda SenigagliesiAlfonso GuarinoLindsay BottomsAlfonso Isidro López DíazLindy BlackburnAlfonso Jose Lopez RiveroLinfeng XuAlfonso Maria PonsiglioneLing ChenAlfonso Martínez-NovaLing Tim WongAlfonso MastropietroLing YuAlfonso MuñozLingfei MoAlfonso Padilla-VivancoLingfu KongAlfonso Prieto GuerreroLingjia GuAlfonso ZambonLingju MengAlfred O. AnkrahLingli YuAlfred SteinLingling MaAlfred TeischingerLingqian ZhangAlfredo CigadaLingwei XuAlfredo De LeoLingxia MuAlfredo FalconieriLingxiang ZhengAlfredo GardelLingxin ChenAlfredo GomesLing-Yun LiAlfredo GonzalezLingzhong GuoAlfredo GüemesLinh NguyenAlfredo HernándezLinh Truong-HongAlfredo J. PérezLinhui SunAlfredo Márquez LuceroLinhui ZhaoAlfredo Moreira Caseiro RochaLinling KuangAlfredo PerisLinmi TaoAlfredo Pinedo-AlvarezLino FigueiredoAlfredo RochaLinpei LiAlfredo Rosado MuñozLinping ZhaoAlgazy ZhauytLinqing LuoAlher Mauricio HernandezLinru XuAli A. ShukurLinyi LiuAli AlmagbileLinyuan XiaAli Al-NajiLinyue GaoAli BakhshinejadLionel NkenyereyeAli BaladorLionel ReveretAli BoolaniLior MedinaAli Caglar ÖzenLipan LeontinaAli CharkheshtLiping DuAli ChelliLiping HuangAli DehghanfirouzabadiLiping QiAli El AmineLi-Ping XuAli Emre KaplanLiqiang LiAli FarmaniLiqiang NieAli H. Husseen Al-NuaimiLiqin CaoAli Hassan SodhroLiqin GeAli HosseininavehLiqing ChenAli Imam SunnyLiqun HouAli Ismail AwadLiqun SunAli JamoosLis K. NanverAli JohariLisa M. GallagherAli Kafash HoshiarLisandro LovisoloAli KazemianLiseane Padilha ThivesAli KouraniLisheng FanAli LalbakhshLisheng JinAli Mehmandoost KotlarLisheng XuAli MirzaeiLishengsa YueAli MohammedLisu YuAli MousivandLitao HanAli PassianLiu WangAli Riza EktiLiuDi JiangAli RohanLiudmila G. ShamanaevaAli TafarojnoruzLiudmila I. Gerasimova-MeigalAli TavallaeiLiutauras MarcinauskasAli ZolfagharianLiuyang ZhangAlia Méndez-AlboresLiuyi LingAlice Ahlem OthmaniLive Steinnes LutebergetAlice BuffiLivia Alexandra Dinu GugoasaAlice GeminianiLívia Florio SgobbiAlice MadoniaLivia PetrescuAlicia E. Torres-GarcíaLivinti PetruAlicia HerreroLivio D’AlviaAlicia TriviñoLiviu C. MicleaAlicja OlejniczakLiviu CiupituAlicja WioraLiviu DutaAlija PašićLiviu VimanAlim SamatLiviu-Adrian CotfasAlin DragomirLi-Wei KangAlin GramaLi-Wei KoAlin ZamfiroiuLiwei LiAlina BarbulescuLiwei LiuAlina CaddemiLixiang LiAlina KarabchevskyLixiang WuAlina PykaLiyan ZhangAlina RoitbergLiyaning (Maggie) TangAlina VasilescuLiza LeeAlina WilkowskaLizeth TorresAlina-Cristina BuneaLizhen WangAlin-Mihai CaileanLizhi PanAliona Ivanovna DregleaLjubomir VračarAlireza AhmadianLlinbo LiAlireza AlamdarLlorenç Cerdà-AlabernAlireza BabaeiLluís AlbarracínAlireza BorhaniLo TiantianAlireza EntezamiLogan T. TrujilloAlireza HaqiqatnejadLoïca AvantheyAlireza MohammadiLoizos PelecanosAlireza TabatabaeenejadLok ShresthaAlisa KozitsinaLonesome MalamboAlisa RudnitskayaLong Chiau MingAlison De Oliveira MoraesLong KangAlison RodgerLong LiAlison ScottLong LingliangAlistair McEwanLong LiuAliyu AliyuLong QiAljoscha RheinwaltLong QueAlkinoos AthanasiouLong T. TruongAllan L. SchaeferLong ZhangAllie AndersonLongjiang LiAllison L. ClouthierLongju LiuAllister GibbonsLongjun DongAllister LoderLongKun GuoAllouis ChristopheLongsheng FuAlma Y. AlanisLongteng TangAlmira RamanavicieneLongxiang GuoAlmudena Diaz ZayasLoredana DumitrascuAlmudena RivadeneyraLorella MarinucciAlmus KenterLorena ParraAlon SchclarLorena Parra BoronatAlonso Alonso-AlonsoLorenzo AngelettiAlp KarakoçLorenzo BrognaraAlper AksacLorenzo CarnevaleAlphus D WilsonLorenzo De CarliAl-Sakib PathanLorenzo FaggioniAlti AdelLorenzo GraziAltino M. SampaioLorenzo IorioÁlvaro AntónLorenzo LottiAlvaro AraujoLorenzo MucchiAlvaro Araujo PintoLorenzo OlivieriÁlvaro Astasio-PicadoLorenzo OrtegaÁlvaro Briz-RedónLorenzo PalmaÁlvaro De La Llana CalvoLorenzo PutzuAlvaro GutierrezLorenzo RicciÁlvaro GutiérrezLorenzo Ricciardi CelsiAlvaro Hernandez AlonsoLorenzo RosaAlvaro Joffre Uribe QuevedoLorenzo ScaleraAlvaro LlariaLorenzo SpinelliÁlvaro Lozano MurciegoLorenzo Teppati LosèAlvaro MaciasLorenzo VangelistaÁlvaro Miguel Carneiro TorrinhaLoreto Di DonatoÁlvaro Moreno SotoLoreto PescosolidoÁlvaro NoriegaLori-Ann R. SacreyAlvaro Parres-PeredoLoris NanniAlvaro RodriguezLoris RovedaAlvaro SuarezLotfi ChaariÁlvaro SuárezLouise AdaAlvise BenetazzoLouise CraigAlwin PouloseLouise DevigneAmad ZafarLoukas BampisAmadeo J. Arguelles-CruzLounis ChermakAmaia Mendez ZorrillaLourdes María Fernández SeguínAmal A. Al-ShargabiLourenco PereiraAmalia Beatriz Orúe LópezLovorka LibricAmalia Floriou-ServouLu BaiAmalia LuqueLu GuoAmalia Luque-SendraLu YuAman BehalLu ZhangAman KaurLua NgoAmanda C. MillsLuana LuginiAmanda EsquivelLuana OlivieriAmani Yousef OwdaLubos BuznaAmanpreet KaurĽuboš OvseníkAmany SarhanLubos RejfekAmarajothi DhakshinamoorthyLuboš SmolíkAmardeep SinghLuca AbeniAmbra FioravantiLuca ArdigòAmbur RamakrishnanLuca BaradelloAmedeo AmoresanoLuca BruschiniAmeena Al SumaitiLuca BruzzoneAmélia Martins DelgadoLuca CalderoniAmelia TrematerraLuca CicalaAmer ZakariaLuca CosmoAmerico CorreiaLuca D’AciernoAmin AminiLuca DavoliAmin AnjomshoaaLuca De StefanoAmin GorjiLuca De VitoAmin Hosseinian-FarLuca Di AngeloAmin KomeiliLuca FerrettiAmin MobasheriLuca FredianelliAmin ShotorbaniLuca LabateAmin TaleiLuca LombardiAmin UllahLuca MagistrelliAmin ZehtabianLuca MartinoAmina SeferagićLuca MassiddaAmir AltafLuca OggioniAmir AsifLuca PallottaAmir BaghdadiLuca Paolo ArdigòAmir BenzaouiLuca PiancastelliAmir GalehdarLuca PiroddiAmir GhiamiLuca ReggianiAmir Haider MalikLuca RosafalcoAmir HandelmanLuca SaniAmir HatamieLuca SantiniAmir HooshiarLuca SchirruAmir Hosein OveisLuca TestarelliAmir Hossein BehbahaniLuca VolleroAmir M. AnvarLucas Blandón-NaranjoAmir Masoud MolaeiLucas De Oliveira TeixeiraAmir MosaviLucas EncarnaçãoAmir NasrollahiLucas F. GerezAmir RahmatiLucas J. KoernerAmir Reza RamtinLucas Prado OscoAmir ShemerLucas V. R. AlvesAmirhossein TehranchiLuci MartinAmirkianoosh KianiLucia BilleciAmirreza AghakhaniLucia Hilario PérezAmirreza KhodadadianLucia NardoneAmit DvirLucia RusuAmit Kumar BatarLucian Gheorghe GruionuAmit Kumar SikderLucian PetrescuAmitabh MishraLucian Stefanita GrigoreAmitava DattaLucian TrifinaAmiya NayakLuciana Freitas BastosAmjad IqbalLuciana LabancaAmjad Y. MajidLuciane Silva MartelloAmmar AhmedLucian-Mihai CosovanuAmmar AlazabLuciano Alves De OliveiraAmmar ArmghanLuciano BertiniAmmar ElsheikhLuciano BoeselAmmar MuthannaLuciano NoceraAmna QureshiLuciano ScaltritoAmol S. PatwardhanLucija BrezočnikAmos NgLucile RutkowskiAmparo Nuñez-AndrésLucinda LeonardAmr AdlyLucja Dybowska-SarapukAmrit AbrolLucjan JerzykiewiczAn Cong TranLucjan KozielskiAna AleksićLucjan SetlakAna B. RuescasLudovico ValliAna ColimLudovit KovanicAna Dinora Guzman-ChavezLu-Hua FuAna DjuricLuige VladareanuAna Fernández-TenaLuigi BenedicentiAna Filipa SequeiraLuigi CapozzoliAna Gonzalez-MarcosLuigi CoppolinoAna Isabel BarbosaLuigi De BellisAna Isabel De Andrés RubioLuigi De NapoliAna Isabel MolinaLuigi De SimioAna Jiménez MartínLuigi Di CostanzoAna Kuzmanić SkelinLuigi FortunaAna MadureiraLuigi La SpadaAna Maria RochaLuigi ManfrediAna NovoLuigi Maria GalantucciAna Paula Marques RamosLuigi MeccarielloAna Rita Elias BrásLuigi VertuccioAna RoviscoLuigi Vito StefanelliAna Vázquez AlejosLuis AbegãoAnabela OliveiraLuis Andreu CaravacaAnahita Jablonski-MomeniLuis AnidoAnamaria BjeloperaLuis Barba-GuamanAna-Maria GalanLuís BernardoAnand ChandrasekharLuís Carlos SantosAnand KoiralaLuis CarúsAnand MishraLuis CoelhoAnand NayyarLuís CorreiaAnand PaulLuis Cruz-PirisAnand PrakashLuis Díaz-BalloteAnand RajuLuis Emanuel Moutinho SilvaAnand Vazhapilli SureshbabuLuis Enrique García-MuñozAnandhakumar SukeriLuis Enrique González JiménezAnando SenLuis Fernando De Mingo LópezAnandram VenkatasubramanianLuis Fernando HerránAnargyros ChatzitofisLuis Fernando Luque VegaAnargyros T. BaklezosLuis Francisco DíezAnastasia AngelakiLuis G. De La FragaAnastasia LavrenkoLuis Gómez DénizAnastasia VybornovaLuis Gonzaga Baca RuizAnastasiia V. BakhchinaLuis HernandezAnastasija CollenLuis Hernández-CallejoAnastasija NikiforovaLuis IglesiasAnastasios DimouLuis Ignacio Lopera GonzálezAnastasios DoulamisLuis J. De La Cruz LlopisAnastasios GotziasLuis Jesús Villarreal-GómezAnastasios KyritsisLuis Jorda-BordehoreAnastasios L. KesidisLuis M. Camarinha-MatosAnastasiya E. RunnovaLuis Miguel Gomes AbegãoAnastassios M. StamatelosLuis MoutinhoAnatoli PopovLuis MoyneAnatoly N. ReshetilovLuis Muñoz-SaavedraAnatoly SavelievLuis N. López De LacalleAnatoly SkripalLuis Naranjo-ZeledónAnca Delia JurcutLuis Omar Colombo-MendozaAnca Loredana UdriștoiuLuis Orozco BarbosaAnca MargineanLuis ParrillaAnca MironLuis Parrilla RoureAnca MorarLuis PayáAnda BelciuLuis Pérez-DomínguezAnder ArriandiagaLuís Pinto Da SilvaAnders BergmanLuís ProençaAnders KaestnerLuis Rodriguez-BenitezAnderson A. FelixLuís RosaAnderson S. L. GomesLuís RoseiroAnderson Wedderhoff SpenglerLuis Sanabria-RussoAnderson Zanardi FreitasLuis Urquiza-AguiarAndon Dimitrov LazarovLuis VelascoAndon LazarovLuisa Gomes PereiraAndoni BeriainLuisa JorgeAndras MarkusLuisa QuevedoAndré AguiarLuiz Antonio BaccalaAndré B.M. SouzaLuiz Carlos Sandoval GoesAndré Caceres CarrilhoLuiz Eduardo Virgilio Da SilvaAndré Eugênio LazzarettiLuiz GonçalvesAndré G. FerreiraLuka PavicAndré HürkampLuka RumoraAndré Luís Ferreira De MeirelesLuka VukićAndré Luís Marques MacatoLukas ChrpaAndré Luiz Lins AquinoLukas ChruszczykAndre Marques-SmithLukáš KučeraAndre MoraLukas MargaritisAndré OttoniLukáš OndicˇAndre PasaLukáš PeterAndré Pimenta FreireLukáš RichteraAndre Schneider De OliveiraLukasz AmbrozinskiAndré SoaresŁukasz BednarzAndrea AbateŁukasz DziudaAndrea BalliniŁukasz JankowskiAndrea BalloLukasz JanowskiAndrea BizzegoŁukasz JanuszkiewiczAndrea BonciLukasz JelenAndrea CaroppoŁukasz KnypińskiAndrea CataldoŁukasz KrzywieckiAndrea CesariniŁukasz KulasAndrea ChiericiLukasz MakowskiAndrea ChiuriŁukasz MalińskiAndrea CioncoliniŁukasz MaślikowskiAndrea CirilloŁukasz OleksyAndrea De MartinLukasz OrmanAndrea DemecoLukasz SadowskiAndrea Di LiddoŁukasz SienkiewiczAndrea Di SchinoLukasz SójkaAndrea EhrmannŁukasz SzeleszczukAndrea FarnhamLukasz WieclawAndrea FasanoŁukasz WyciślikAndrea FranciniLulu SunAndrea FuscoLulu WangAndrea GaltarossaLuminita GhervaseAndrea GilioliLuminiţa MoraruAndrea GiorgettiLun-Chi ChenAndrea Gonzalez-MontoroLunke FeiAndrea LechiancoleLuo LiuAndrea LoddoLurui ZhaoAndrea LuttgenLuz Berenice López-HernándezAndrea MaioLuz Del Carmen Gómez-PavónAndrea ManzoniLuzheng BiAndrea MarinLvwen HuangAndrea MariscottiLydia Min-Ying SuAndrea MarottaLydia SobotovaAndrea MasciadriLynda SedikkiAndrea MasieroLyubka PashovaAndrea MescolaLyudmila DimitrovaAndrea MichelM. A. Hannan Bin AzharAndrea NicolòM. Ariel WallaceAndrea PerucchiM. Gomez-GonzalezAndrea PirasM. Mahmood AliAndrea ProtoM. Mustafa RafiqueAndrea PucciM. Pilar GardeAndrea RiaM. Salauddin RaselAndrea RomanoniM. Shamim KaiserAndrea ScorzaM. Teresa Teixidó UllodAndrea TangherloniM. Z. NaserAndrea TigriniMª Nieves Piña CapóAndrea TonoliMaanak GuptaAndrea ValenteMaarten WeynAndrea VitaliMacedon MoldovanAndreas BirkMaciej GucmaAndreas BorgschulteMaciej HarasAndreas BraeuerMaciej HojdaAndreas BraunMaciej JedlińskiAndreas D. TsigopoulosMaciej KlaczynskiAndreas FischerMaciej KubońAndreas FlorosMaciej MajewskiAndreas GeorgopoulosMaciej MatuszewskiAndreas HeinMaciej MatysAndreas HoffmannMaciej NikodemAndreas KalogeropoulosMaciej NowakAndreas KelerMaciej RomaniukAndreas KönigMaciej S. WróbelAndreas LarentzakisMaciej StefańczykAndreas PenirschkeMaciej SułowiczAndreas PesterMaciej TrusiakAndreas RauhMaciej WojtczakAndreas RichterMaciej ZaborowiczAndreas RoncatMaciej ZajkowskiAndreas SchützeMadaín Pérez PatricioAndreas StadlbauerMadalina DumitriuAndreas TsakalofMadalina Simona BaltatuAndreas WeberMadalina-Georgiana BerceanuAndrei AksjonovMădălin-Dorin PopAndrei BorșaMadallah AlruwailiAndrei FotiadiMadhu GyawaliAndrei GheorghiuMadhu SheriAndrei I. MaimistovMadis RatasseppAndrei KirilenkoMaeda HiroakiAndrei LavrinenkoMagdalena CzarnogorskaAndrei OlaruMagdalena Igras-CybulskaAndrei PotapovMagdalena KachniewskaAndrei RotaruMagdalena KopernikAndrei StanMagdalena Król-ZielińskaAndrei StancalieMagdalena KusnierzAndrei SurženkovMagdalena MachnoAndrei TarasovMagdalena MieloszykAndrei TsarevMagdalena Niemczewska-WójcikAndrei VasilateanuMagdalena PalaczAndrei Vasile NastutaMagdalena PilarskaAndrei VatavuMagdalena StobieckaAndrei VladykoMagdalena SzymczykAndreia FreitasMagdalena Valentina LunguAndrej AnžlinMagdalena WłodarskaAndrej BugajevMaged Mahmoud MarghanyAndrej GrguricMaggie HeAndrej JermanMagna GabrieleAndrej KastrinMagnus HolmAndrej M. SavićMagnus WesterlundAndrej NovákMahdi HeydariAndrej SarjasMahdi NaghshvarianjahromiAndrej ZgankMahdi RezapourAndreja Trojner-BregarMahdi TakaffoliAndrés Arcia-MoretMahdi YousefiAndres Blanco OrtegaMahdi ZareeiAndrés G. MarrugoMahendra PiraveenanAndrés García FlorianoMahendra ShuklaAndrés J. PrietoMaher KhalielAndrés Omar TiseiraMahesh SoniAndres ÖpikMahima Agumbe SureshAndres RubianoMahmood ShafieeAndrés Sepúlveda AllendeMahmoud ElbattahAndrés Trujillo-LeónMahmoud ElsisiAndrew AquilaMahmoud HassanAndrew BellMahmoud NabilAndrew HamiltonMahmoud NasrAndrew HatchettMahmoud OudaAndrew HerschfeltMahmoud R. M. AtallaAndrew J. HarvieMahmoud WagihAndrew J. KolarikMahyar KhorasaniAndrew M. GeronimoMai The VuAndrew MaloneMaik HerbigAndrew MesoMaiko SakamotoAndrew P LapointeMailyn Pérez LivaAndrew P. LavenderMaira Beatriz Hernandez MoranAndrew PaiceMaira Martins Da SilvaAndrew PetersMaja BrykAndrew R. HarrisMaja GoršičAndrew R. WillisMaja KrčumAndrew RevillMaja MateticAndrew WardMaja PusnikAndrew WixtedMajeed AbdulAndrew WoodMajid HosseinpourAndrey A. ZhirnovMajid KhazaeeAndrey AfanasievMajid Mufaqam Syed-AbdulAndrey AlekseenkoMaju KuriakoseAndrey ChernovMakhsudsho G. NematovAndrey D. PryamikovMakiko KobayashiAndrey EliseyevMakoto AokiAndrey GalyaevMakoto HasegawaAndrey Georgievich PaulishMakoto MiyakoshiAndrey I. Poddel’skyMalak HenchiriAndrey KoptyugMalcolm JoyceAndrey LavrinenkoMalgorzata DominoAndrey LunkovMałgorzata I. MichalczykAndrey OsipovMalgorzata Klaudia GuzowskaAndrey PryamikovMałgorzata NorekAndrey StepnovMałgorzata OchotaAndrey V. MityakovMalgorzata OrczykAndri RiidMalgorzata PankowskaAndrija BernikMałgorzata Plechawska-WójcikAndrius ČeponisMalgorzata Renigier-BilozorAndrius DzedzickisMalgorzata SyczewskaAndriy SemenovMałgorzata SztubeckaAndrzej BębenMałgorzata WojtaszekAndrzej BieniekMalick DiakhateAndrzej BrudnickiMalik HaddadAndrzej BuchowiczMalik M. Naeem MannanAndrzej BurghardtMalinka IvanovaAndrzej Eugeniusz StatecznyMalte FrovelAndrzej FelskiMalte MisolAndrzej GajewskiMamede De CarvalhoAndrzej GnatowskiMamoona HumayunAndrzej JanutkaMamta AgiwalAndrzej KarbowskiMamtha BallaAndrzej KasperskiManabu AtakaAndrzej KatuninManabu SatoAndrzej KaźmierczakManabu TsukadaAndrzej KoszewnikManasvi LingamAndrzej KotyraManel Del ValleAndrzej KrukManel TaboadaAndrzej KubitManish BhattAndrzej KudelskiManisha SahuAndrzej ŁebkowskiManlio BaccoAndrzej MarcinkowskiManmeet Mahinderjit SinghAndrzej MaterkaManob Jyoti SaikiaAndrzej MichalskiManod WilliamsAndrzej MituraManohar DasAndrzej MyśliwiecManohar Prasad BhandariAndrzej NowrotManoj Kumar MahataAndrzej OsuchManoj PandaAndrzej PacanaManolis TsiknakisAndrzej PaszkiewiczManos PanaousisAndrzej PawlowskiMansur AsAndrzej PerecManuel A. ArmadaAndrzej PolanczykManuel AleixandreAndrzej PrzybyłManuel ArbeloAndrzej SiomaManuel Arias-MontielAndrzej SkoczeńManuel ArrebolaAndrzej StatecznyManuel Au-Yong-OliveiraAndrzej TomporowskiManuel Bou-caboAndrzej TypiakManuel BuitragoAndrzej WłochManuel CabaleiroAndrzej ZankiewiczManuel De La Torre JuarezAndy StammManuel Eugenio Morocho-CayamcelaAndżelika KrupińskaManuel F. González-PenedoAnerise De BarrosManuel Fernández-VeigaAneta HerbutManuel FerreAneta Nowak-MichtaManuel Filipe Pereira Cunha Martins CostaAneta Poniszewska-MarandaManuel GablerAneta PrijićManuel García GarcíaAneta Ptak-ChmielewskaManuel García SánchezAneta SlodekManuel GrañaAnfeng LiuManuel GüntherAngel Carreño-OrtegaManuel Gutiérrez-CapitánAngel CaţaronManuel HerreraAngel Ciprian CormosManuel J. C. S. ReisAngel DiéguezManuel J. FonsecaÁngel Giménez PastorManuel Jesús Domínguez-MoralesAngel JimenezManuel Joaquim Bastos MarquesÁngel Morales-GarcíaManuel Joaquim Sabença FelicianoAngel MurManuel Lopez-MartinAngel Perez-CruzManuel MartinezAngel RodriguezManuel MorenoÁngel Ruiz ZafraManuel OcañaAngela Di VirgilioManuel Omar Meranza-CastillónAngela DigulescuManuel PereaÁngela Fernández PascualManuel Prado-VelascoAngela LombardiManuel Rodriguez-MartinAngela RepanoviciManuel Rodriguez-RastreroAngélica De AntonioManuel Sierra-CastañerAngélica Román GuerreroManuel Utrilla-MansoAngeliki KritikakouManuel V. RamalloAngeliki PapalouManuel Vargas VillanuevaAngelo ColucciaManuela AvadaneiAngelo GalanteManuela ChessaAngelo GulinattiManuela IngaldiAngelo MarcelliManuela ZudeAngelo Marcelo TussetMao MaoAngelo OdettiMao ShanAngelo TroedhanMaohua LinAngelos AmditisMaohua PanAngelos KarlasMaokun LiAngelos P. MarkopoulosMaolin ChenAngga HermawanMaomao ChenAnh Thu Phan-HoMaorong GeAnh TranMaosen WangAnh TruongMaosi ChenAnh-Tu NguyenMar Vilanova De La TorreAnhtuan LeMaram Bani YounesAnia CraveroMarat MukhametzhanovAnil B. ShriraoMarc BrechtAnil Kumar KhambampatiMarc DuquennoyAnil ShriraoMarc FrincuAnirban ChowdhuryMarc KurzAnirban PaulMarc Marín-GenescàAnirban SinhaMarc Mateu-MateusAniruddha KaushikMarc SolalAniruddha RayMarc VidalAnisa KaurMarc WeinbergAnish Prasad ShresthaMarc WittmannAnja HesselbarthMarcel AmelootAnja KeskinarkausMarcel BouvetAnja SchlichtMarcel FajkusAnjan GudigarMarcel KordošAnkana KakotiMarcel KurucAnkit JhaMarcel NicolaAnkit RavankarMarcela Alina FărcașiuAnkita SinhaMarcela Bindzárová GergeľováAnn L. BaldwinMarcela MuneraAnna A. OkunkovaMarcello BertoAnna BanaszekMarcello CostaAnna BarneyMarcello Di RisioAnna BazanMarcello IasielloAnna Bazan-KrzywoszańskaMarcello Magri AmaralAnna BergamaschiMarcello SanguinetiAnna Chachaj-BrekieszMarcelo Bender PerotoniAnna Cleta CroceMarcelo DavidAnna D’AuriaMarcelo FariaAnna GorbenkoMarcelo Félix AlonsoAnna GubalMarcelo FernandesAnna HadamusMarcelo GarcíaAnna IlnickaMarcelo GitiranaAnna Jamroz-WisniewskaMarcelo Hazin AlencarAnna Kaminska-ChuchmalaMarcelo NogueiraAnna Knitter-PiątkowskaMarcelo PetryAnna KobusińskaMarcelo Sampaio De AlencarAnna KruspeMarcelo WerneckAnna KuznetsovaMárcia BarrosAnna M. RakoczyMárcia C. FigueiredoAnna MamouMarcin AdamczykAnna MichalakMarcin BalcerzykAnna N. BerlinaMarcin BernaśAnna PakulaMarcin ChodźkoAnna Paradowska-StolarzMarcin CiecholewskiAnna PorębskaMarcin DerlatkaAnna ScandurraMarcin DerwichAnna SiedliskaMarcin DrozdAnna Sołtysik-PiorunkiewiczMarcin GnybaAnna StaerzMarcin GórskiAnna TriantafyllouMarcin HabrychAnna UmbertMarcin JagodaAnna VaskuriMarcin JesionekAnna VilàMarcin KafarskiAnna VisviziMarcin KicinskiAnna Zawada-TomkiewiczMarcin KluczykAnnalisa D’ArcoMarcin KolakowskiAnnamaria CostaMarcin KorzeńAnnarita TedescoMarcin KowalskiAnne MichelinMarcin KremieniewskiAnne PallarèsMarcin LackowskiAnne RoskenMarcin LawnikAnne SchwarzMarcin LucknerAnnegret MündermannMarcin NabiałekAnne-Maria LaukkanenMarcin PiekarczykAnnica KristofferssonMarcin PlucińskiAnnika K. JägerbrandMarcin RudzkiAnoop ChauhanMarcin SpiralskiAnsgar SchwirtzMarcin StaniekAnshu RastogiMarcin StraczkiewiczAntal HibaMarcin SuszynskiAntanas CenysMarcin UrbanowiczAnthony CahillMarcin Wa̧torekAnthony CampbellMarcin WitczakAnthony ChoiMarcin WozniakAnthony CoustouMarcin ZychAnthony DichiaraMárcio Falcão Santos BarrosoAnthony G. ConstantinidesMárcio Júnior LacerdaAnthony J. ClarkMarcius Fabius De CarvalhoAnthony Neal WatkinsMarco A. Moreno-ArmendárizAnthony PamartMarco A. Panduro PanduroAnthony WakerMarco AbbatangeloAnthoula GaigneauxMarco Alexandre RibeiroAntoine DupréMarco Antonio Álvarez GonzálezAntoine FournierMarco Antonio PerezAntoine SchmeitzMarco Antonio Sotelo MongeAntoine VacavantMarco Antonio TeixeiraAntoine Weill-DuflosMarco ArnesanoAntolino GallegoMarco Aurelio Nuño-MagandaAntolino Gallego MolinaMarco BalsiAnton AntonovMarco BaùAnton GradišekMarco BernardoAnton IlievMarco BindiAnton KonevMarco BobingerAnton KonushinMarco BonoperaAnton KorniienkoMarco BottaAnton KosMarco BraviAnton ManakhovMarco BuiattiAnton PletersekMarco BuzzelliAnton PopovMarco CaniatoAnton RassõlkinMarco CapogniAnton S. BychkovMarco Cardenas-JuarezAnton S. TarasovMarco CarminatiAnton UmekMarco CarotenutoAnton VershovskiiMarco CarratùAntonella CarbonaroMarco CassinelliAntonella CurulliMarco CeccarelliAntonella D’AlessandroMarco CicalaAntonella FaliniMarco CicconeAntonella TatarelliMarco CiveraAntonello SantiniMarco Claudio De SimoneAntoni GrauMarco CocconcelliAntoni Llopis-LorenteMarco CostanzoAntoni MartinezMarco CrescentiniAntoni NowakowskiMarco D. Vásconez-MazaAntoni Perez-NavarroMarco D’AlessandroAntonia LopresideMarco D’AlonzoAntonija TadinMarco Dell’IsolaAntonin PlatilMarco DepianteAntonino CanaleMarco EspositoAntonino LaudaniMarco FaggellaAntonino MasaracchiaMarco FilippucciAntonino NaroMarco FuringhettiAntonino NatalelloMarco GabicciniAntonino PietropaoloMarco GadolaAntonino ProtoMarco GazzoniAntonino QuattrocchiMarco GermanottaAntonino QuattroneMarco GervasiAntonino S. FiorilloMarco GhislieriAntonino ScandurraMarco GrossiAntonio A. AguiletaMarco IosaAntonio A. MoyaMarco KnaflitzAntonio AlbiolMarco LapegnaAntonio Alex AmorMarco LaraccaAntonio BalzanellaMarco LaurinoAntonio BanderaMarco LeoAntonio BartolomeoMarco LimongielloAntonio CaggianoMarco ListantiAntonio Carlos De Oliveira JúniorMarco LombardiAntonio Carlos SobieranskiMarco MaggialiAntonio CicchellaMarco MandoliniAntonio CilfoneMarco MarconAntonio ComparettiMarco MartaloAntonio CorradiMarco MatassoniAntonio CorteseMarco MauriAntonio CostanzoMarco MercuriAntonio CuccaroMarco MiliucciAntonio Di BartolomeoMarco MiniaciAntonio Di MaioMarco MobilioAntonio E. Jimenez-CanoMarco P. Soares Dos SantosAntónio Espirito-SantoMarco Pérez-HernándezAntonio EspositoMarco PiccirilliAntonio F. Díaz GarcíaMarco PiconeAntonio Fernández-CaballeroMarco PortaAntonio Fernandez-LopezMarco PortelliAntonio FormisanoMarco ReggianniniAntonio GarcíaMarco RianiAntonio Garcia-JerezMarco RighiAntonio GesualdoMarco Roberto CavallariAntonio Guillen-PerezMarco RosoneAntónio J. R. NevesMarco SampietroAntonio J. RodríguezMarco Sanna AngotziAntonio J. Sanchez-SalmeronMarco SantonicoAntonio J. Torija MartinezMarco ScharringhausenAntonio Jesus FernandezMarco SchiavoneAntonio Jesús Yuste DelgadoMarco Stefano DemarchiAntonio José Calderón GodoyMarco TalliniAntonio José Estepa AlonsoMarco TreguaAntonio José Lozano GuerreroMarco VaccariAntonio JuniorMarco Vinicio Hernández ArriagaAntonio LázaroMarco ZappatoreAntonio Luiz Pereira De Siqueira CamposMarcos ArzaAntonio M. G. TommaselliMarcos Benedito SchimalskiAntónio M. LopesMarcos CavenaghiAntonio ManzaliniMarcos De Oliveira JuniorAntonio Matencio EscolarMarcos Díaz QuezadaAntónio Miguel MorgadoMarcos Eduardo ValleAntonio Miguel Ruiz ArmenterosMarcos F. S. TeixeiraAntonio MinopoliMarcos Quiñones-GrueiroAntónio MonteiroMarcos RodriguesAntonio MoschittaMarcos SchimalskiAntonio MuñozMarcos Silva MartinsAntonio NovelliMarcos Tostado-VélizAntonio OliveiraMarcus HammerAntonio OrlandiMarcus OW GrimmAntonio PardoMarcus SchmidtAntonio ParzialeMarcus Tullius ScottiAntonio PepeMarcus VaranisAntonio Pérez-GonzálezMarcus VollmerAntonio PescapèMarderos SayeghAntonio PetošićMare KoitAntónio PintoMarek AmanowiczAntónio RamosMarek BarskiAntonio RicchiMarek BolanowskiAntonio Rios-NavarroMarek BugajAntonio Riul, Jr.Marek DługoszAntonio Robles-GómezMarek FraštiaAntonio Ruiz MartínezMarek GancarzAntonio Sarasa-CabezueloMarek JaskiewiczAntonio SarolicMarek KonefałAntonio SorianoMarek KozieńAntónio SousaMarek KraftAntonio SuppaMarek KrawczukAntonio ValenteMarek LampartAntonio Valenzuela GutiérrezMarek LefikAntónio VieiraMarek MagdziakAntonios MouratidisMarek MiskowiczAntonios X. LalasMarek MoravcikAntonis AlexandridisMarek PenhakerAntoon LigtenbergMarek PokornyAntreas TheodosiouMarek ProchazkaAntti LehikoinenMarek PrzyborskiAntti VehkaojaMarek SawickiAnu G. BourgeoisMarek SikoraAnuar De Jesus Fernandez OlveraMarek SłońskiAnubhav SrivastavaMarek StencelAnuchit JitpattanakulMarek SzlósarczykAnuj TiwariMarek UrbanikAnuradha KarMarek VagašAnuradha SinghMarek WęglowskiAnuran MakurMarek WesołowskiAnuroop GaddamMarek ŻyczkowskiAnurup DattaMarek ZygmuntAnusha WithanaMaren FriesenAnusuya PalMargarita Tecpoyotl-TorresAnxi YuMargarita-Arimatea Diaz CortesAn-Yeu WuMargherita CapriottiAnyoji MasayukiMargherita PeruzziniAphrodite KtenaMaria Adele GiamberardinoApostolos AxenopoulosMaria Alexandra TeodósioApostolos GkamasMaria Angeles Caballero-MoraApostolos KorlosMaría Ángeles Verdejo EspinosaApostolos KousaridasMaria Antonietta FerraraApostolos PapathanassiouMaria António CastroApostolos TsagarisMaria Baldeon CalistoApostolos XenakisMaría Blanca CamineroArabas PiotrMaria BostenaruAraliya MoslehMaria ButakovaAram Ter-SarkisovMaría Campo-ValeraArangarajan VinayagamMaría Carmen Blanco-LópezArantxa UrangaMaría Carmen CarneroArash AhmadivandMaría Carmen PérezArash AkbariniaMaria Colomba ComesArash JenabMaría Concepción Pérez CárcelesArash M. DizqahMaría Criado SanzArash TakshiMaria C.T. CangussúArata SuzukiMaria D. MirandaArati SridharanMaria Da Graca Campos PimentelAravin Prince PeriyasamyMaria De Lourdes Bueno Trindade GaloAravind Sukumaran RajamMaria Del Carmen Romero-GarcíaAravinda RaoMaría Dolores Fernández RamosArbnor PajazitiMaría Dolores Gómez PulidoArcangelo MerlaMaria Eduarda FerreiraArdavan RahimianMaria Elisa TataAre Hugo PrippMaria Erminia GenoveseArezoo EmadiMaría Estrella Sousa VieiraArfan GhaniMaria Fátima PaulinoArgyris N. StassinakisMaria Francesca PiacentiniArgyris ToubekisMaría Francisca CarreñoArgyro-Maria BoutsiMaría Francisca Rosique ContrerasAri HamdaniMaria Gabriela LagorioArianna PesciMaria Gabriella XibiliaArie C. SeijmonsbergenMaria Gamella CarballoAriel DzwonkowskiMaría García FernándezAriel LellouchMaria Giovanna BiancoAriel Soares TelesMaria Giovanna MasciottaArif MustafazadeMaria Giuseppina LimongelliArif Ul AlamMaria Graça RuanoArifur R. KhanMaria Grazia GrimaldiArijit GhoshMaria Grazia ManeraArinc OzturkMaria GritsevichArindam ChakrabartyMaría Isabel Prieto BarrioArindam DuttaMaría Isabel Rodriguez FerradasAris PsilovikosMaria Jesus De La Fuente AparicioAris TsangrassoulisMaría Jesús Lobo-CastañónAris TsolisMaría José García-PolaAristeidis TsagkarisMaria Jose Gomez-SilvaAritz Bilbao-JayoMaria K. RybarczykArjun NeupaneMaria KaratassiouArkadiusz DobrzyckiMaria KihlArkadiusz GardeckiMaria KonstantakiArkadiusz KampczykMaria KoroziArkadiusz MystkowskiMaria KrechowiczArkadiusz StanulaMaria Lucia MigliettaArkadiusz SzarekMaria Luisa BraungerArkadiusz TomczykMaria Luisa VillaniArkadiusz ZarzyckiMaria Luz Rodriguez-MendezArkady KaryakinMaria Maddalena CalabrettaArkady RedkinMaria Magdalena Bujnowska-FedakArkady SerikovMaria MatsiolaArlindo SilvaMaria Mendoza-MuñozArmando BarretoMaria MrówczyńskaArmando FerreiraMaría NapalArmando J. PinhoMaria NishevaArmando RicciardiMaria PanitsaArmin LehmannMaria PedreroArmin MasoumianMaria Pia ViggianoArmin MehrabiMaría Pilar Pecci LloretArmin ParavlicMaria PopArmin SchmidtMaria R BonsignoreArmir BujariMaría R. Fernández-RuizArnab PalitMaria Ramos PayánArnaldo BatistaMaria RaposoArnaldo Leal-JuniorMaria RashidiArnau Mir TorresMaria RigouArnaud CuissetMaria RizziArnaud GouelleMaria RomanoArnaud Le BrisMaria RubegaArnaud PeizeratMaria SeimeniArne KüderleMaria SeleznevaArnulfo Ramos-JiménezMaria SergeevaÁron TörökMaria SileoAroutin KhachaturianMaria StrianeseArpad GellertMaria SurmenevaArpad KarsaiMaria Teresa Baeza-RomeroArsénio ReisMaría Teresa García-OrdásArslane Hamza-CherifMaría Teresa LázaroArta DiloMaría Teresa LópezArtak HeboyanMaría Teresa Sanz-PascualArtem A. KuznetsovMaria VasconcelosArtem KruglovMaria Vesna NikolicArtem PadokhinMaría Victoria SebastiánArtem Sergeevich TrifonovMaría Yolanda Castellote-CaballeroArtem V. NikonorovMaria-Alexandra PaunArtemios KarvounisMariachiara RicciArtemissia-Phoebe NifliMariacrocetta SambitoArthur DizonMariah HoskinsArthur I. PetrovMaría-José Terrón-LópezArthur MolnarMaría-Luisa Pérez-DelgadoArtjoms ObushevsMarian ApostolArtur BanachMarian BresticArtur BejgerMarian DrusaArtur CzerwińskiMarian GaiceanuArtur GafurovMarian IonArtur Jorge FerreiraMarián KučeraArtur KierzkowskiMarian LopatkaArtur M. GafurovMarian MarčišArtur MakarMarian ŠofrankoArtur MaurinMarian VladescuArtur PałaszMarian WiwartArtur PolińskiMarian WysockiArtur RydoszMariana Alfaro-GómezArtur SpivakMariana BusilaArtur Tadeusz KrzyżakMariana De Jesus Paiva ProençaArtur WilkowskiMariana FragaArturas KaklauskasMariana KolbergArtūras KilikevičiusMariana MarinArturas SerackisMariana PetrovaArturo Aparicio GilMariangela ValloneArturo Garcia-PerezMarianne MaktabiArturo Montejo-RáezMarianne PerrinArturo Reyes-GonzálezMarianne SilvaArturo VeraMariano BrescianiArtyom Dmitrievich ObukhovMariano GalloArumugam SangiliMariano SerraoArun ArjunanMariapia FaruoloArun JaganathanMarica FranziniArun Kumar SangaiahMarie BalkováArun Kumar SomaMarie-Josépha YoussefArun Prakash PeriyasamyMariela PadillaArūnas LukoševičiusMarie-Luce BourguetArunkumar ChandrasekharMarietta FodorArup K. GeorgeMariia NazarkevychArvind UpadhyayMariia PukalchikArxel De LeónMarija Brkic BakaricAsaad AlmssadMarija JozanovićAsami KenichiMarija KlopčičAsanga UdugamaMarija KrsticAsanka G. PereraMarijo BuzukAsanthi JinasenaMariko NakanoAsdrúbal López-ChauMarilena RonzanAsem M. AliMarin MarinAsha RaniMarin MarinovAshish GoelMarina BolsunovskayaAshish RauniyarMarina BonomoloAshish SaxenaMarina IndriAshley LyonsMarina KhazovaAshok ChhetryMarina ManeaAshok Kumar PatilMarina MatosAshok VaseashtaMarina MirandaAshrafizadeh Seyed NezameddinMarina RestaAshton TheakstoneMarina RumyantsevaAshutosh BhardwajMarina TopaAshutosh Dhar DwivediMarinella CocoAshutosh SharmaMarinella GiuntaAshwin AshokMarinko BarukčićAshwin Kumar MyakalwarMarino VilovićAshwin Ramesh BabuMario A. Quiroz-JuárezAsif KhanMario Alvarado-LorenzoAsim KhanMario AntunesAsiya MustafinaMario A.R. DantasA.S.M. KayesMario CesarelliAsm ShihavuddinMario Eduardo Rivero-AngelesAsma AdnaneMario FargnoliAsma FallahMario GuevaraAssunta TaverniseMario HirzAsta MikalauskienėMario IodiceAsterios LeonidisMário João S. F. SantosA.T. Ezhil VilianMário José Gonçalves Cavaco MendesAtakan SahinMario KusekAtanu JanaMario L. RuzAtefeh Hajijamali AraniMario Luis Ruz RuizAtefeh KarimzadehMario ManzoAtena Roshan FekrMario MarchettiAthanasios AnastasiouMario MarinelliAthanasios DimitriouMario Martinez-ZarzuelaAthanasios KoutrasMario Massimo FogliaAthanasios NikolaidisMario MeroneAthanasios RakitzisMario MilicevicAthanasios TiliakosMario MiscuglioAthanasios VasilakosMario MontagudAthanassios G. KanatasMário Nuno MataAthena RoumboutsosMario Ortiz GarcíaAthina AlexopoulouMario PardoAthina TsanousaMário Pereira VéstiasAthos TrecrociMario PrsaAtiđa SelmaniMário ReisAtif ShahzadMario Ricardo Gongora-RubioAtis ElstsMario RothbauerAtslands RochaMário S. RodriguesAtsushi FukasawaMario SicilianiAtsushi HosokawaMario TamagnoneAtsushi ItouMario ValderramaAtsushi K. KonoMário VazAtsushi KimuraMario VerdicchioAtsushi MitaniMario VersaciAtsushi MotogaitoMário VéstiasAtsushi TeramotoMário W. L. MoreiraAtta Ur RehmanMario WallnerAttila FrankoMarioara AvramAttila HiltMariofanna MilanovaAttila KerteszMariolino De CeccoAttila KissMarion MundtAttila NagyMarios AnagnostopoulosAttila TothMarios RaspopoulosAtul KulkarniMarios SophocleousAtul SajjanharMarios TzouvarasAudrius ČereškaMarisa MaximianoAudrius DedeleMarisa NicolaiAudrone DumcieneMarius BumbacAugusto Cesar Da Silva BezerraMarius CoriciAugusto CiuffolettiMarius GavrilescuAugusto MontisciMarius Iulian MihailescuAugustyn LorencMarius MineaAuk KimMarius PrelipceanuAuksė EndriulaitienėMarius SchneiderAurel Florentin Cătălin NegrilăMarius VochinAurelian MarcuMarius VolmerAurélie TourniéMarius-Constantin VochinAurélien ArnaubecMariusz BajgerAurélien DantanMariusz BuciakowskiAurelija BurinskienėMariusz DąbrowskiAurelio BermúdezMariusz DruźbickiAurélio Luiz Magalhães CoelhoMariusz GiergielAurelio MeloMariusz KłonicaAurore Avarguès-WeberMariusz KormanekAvanish MishraMariusz KostrzewskiAvgis HadjipapasMariusz MajdańskiAvi KarsentyMariusz OszustAvinash SharmaMariusz P. WilkAxel BoeseMariusz PelcAxel BuchnerMariusz RząsaAxel HahnMariusz SpechtAxel SikoraMariusz SzwochAyad M. Fadhil Al-QuraishiMariusz TryznowskiAyako NakaneMariusz UchrońskiAyal AnisMariusz WęglarskiAyan ChatterjeeMariusz ZiejaAyaz AhmadMariya Konsulova-BakalovaAydar NasybullinMarjan GolobAyham ZaitounyMarjan MansourvarAykut TamerMarjan MernikAyman El-ZohairyMark A. ArnoldAymen FlahMark AuslenderAyşe BozlakerMark BerhowAyush DusiaMark CarrollAzhar AbbasMark Cronin-GolombAzhar ZamMark F. HansenAzita GoudarziMark J. PotosnakAziz AmineMark JacksonAziz ShahMark Kenneth QuinnAzlan ZahidMark LevinAzra TafroMark LowryAzwirman GusrialdiMark McCormickAzzeddin NagharMark MelnykowyczB. N. Pavan KumarMark MuellerBabak JamaliMark PotterBabak MohammadiMark RollBabak Shahian JahromiMark SchulzBabette GötzendorferMark T. MarshallBabu R. DawadiMarkéta PotůčkováBac NguyenMarkku LöytönenBader AldawsariMarko BizjakBadicu GeorgianMarko HölblBahaa AnsafMarko JermanBahman JavadiMarko JurcevicBahman MoraffahMarko KaticBahram LaviMarko KosBahram RavaniMarko MäkynenBai LiMarko MalajnerBai XueMarko MatulinBaifan ChenMarko PetkovsekBakhtiar FeizizadehMarko SimicBakhtiyar OrazbayevMarko StupinBala MuralikrishnanMarko SvacoBalakrishna GokarajuMarkos G. TsipourasBalázs HeiligMarkos PetousisBalázs KocsiMarkus BuchmannBalázs NagyMarkus FeldtBalbina Casas-MéndezMarkus FiedlerBambang KuswandiMarkus JobstBander A. AlzahraniMarkus M. HoffmannBaniya BabuMarkus UlmschneiderBaodi LiuMarkus VinczeBaoding ZhouMaros SmondrkBaodong XuMarouan MizmiziBaofeng GuoMarshed Kassim MohamedBaoguo XuMart MinBaohua QiangMarta BełkaBaoping CaiMarta Campos FerreiraBaoqi SunMarta Doval MiñarroBaoye SongMarta Elena Losa IglesiasBappaditya MandalMarta Ferreiro-GonzálezBaptiste ChometteMarta Fiedot-TobołaBarath RaghavanMarta JarczewskaBarbara BezerraMarta KadelaBarbara ContieroMarta LorenziniBarbara FabbriMarta PeñaBarbara George-JaeggliMarta Ruiz LlataBarbara GierobaMarta RusnakBarbara GiussaniMarta SigutBarbara GuidiMarta Sylvia Del RioBarbara KleinMarta TessaroloBarbara Koroušić SeljakMarta Wlodarczyk-SielickaBarbara LeporiniMarta ZanolettiBarbara M. MasiniMarta Zurek-MortkaBarbara S. ChaparroMartha Arbayani ZaidanBarbara StrugMartha VardakiBarbara VercelliMartin AugustynekBarbara VillariniMartin BarczykBarbara WagnerMartín Barrère CambrúnBardia YousefiMartin BauerBaris GabrieleMartin BeerBarrie Hayes-GillMartin BitterBarry EvansMartin BoldtBarry LavineMartin BorošBart PlovieMartin BrandlBart SpronckMartin BurgdorfBartłomiej AmbrożkiewiczMartin ĆalasanBartlomiej BlachowskiMartin ČížekBartłomiej ĆmielewskiMartin DaumillerBartłomiej GuzowskiMartin GeyerBartłomiej SzewczykMartin GoubejBartłomiej TorońMartin GulanBartolomeo Della VenturaMartin HagmüllerBartosz DalewskiMartin HempelBartosz MarcinkowskiMartin Hering-BertramBartosz MillerMartin J. StraussBartosz MindurMartin J.-D. OtisBartosz MitkaMartin JuddBartosz SawikMartin KasparickBartosz SzelągMartin KenyeresBartosz SzulczyńskiMartin KlempaBartosz WieczorekMartin KopaniBas PetersMartin MaguireBasaran Bahadir KocerMartin MoskoBasil Mohammed Al-HadithiMartin OtisBasilio PueoMartin PechBasilio VescioMartin PollákBassel Al HomssiMartin RichterBassem Abd-El-AttyMartin RobinsonBastian EngelmannMartín RodríguezBastien ConfaisMartin Romero-SanchezBasuraj BhowmikMartin RožánekBaudilio Coto GarcíaMartin SchätzBavencoffe MaximeMartin SchmittBayu Adhi TamaMartin ŠipošBayu Taruna Widjaja PutraMartin Valtierra-RodriguezBeat GöpfertMartin VelasBeata HejmanowskaMartina BanchelliBeata KurcMartina CaramentiBeata Łopaciuk-GonczarykMartina ManciniBeata MarciniakMartina Medvidović-KosanovićBeata Paczosa-BatorMartina Teresa BevacquaBeata PalczynskaMartina VettorettiBeáta StehlíkováMartine NeckebroekBeata ZimaMartino AldrigoBeatrice ArvintiMartino GiaquintoBeatrice MotellaMartino MariniBeatriz Garcia-BañosMartins FerreiraBeatriz GomesMartiwi Diah SetiawatiBeatriz Gómez-MartínMartynas PatašiusBeatriz Jurado SánchezMarvin Coto-JiménezBeatriz L. BoadaMarvin R. PaulsenBee Kang TanMarx Leandro Naves SilvaBeelee ChuaMary Beth ArensbergBegoña C. ArrueMaryam AminiBehnam AtazadehMaryam DoborjehBehnam DezfouliMaryam SalehiBehrokh BeiranvandMaryamsadat TahavoriBehrooz H. YousefiMarzieh H. DerkaniBehzad Behzad FarahaniMasahiko AkiBehzad V. FarahaniMasahiro FujitaBehzad VaferiMasahiro SeikeBeijia LiuMasaki MichihataBeilei WuMasanori HaraBeiyu LinMasaomi IkedaBéla GengeMasashi SuganoBéla TakaricsMasato SoneBelal AhmadMasato YasumotoBelén CurtoMasaya TodaBen CerjanMasayasu MieBen HalkonMasayuki TanabeBen JohnsonMashaalah ZarejousheghaniBen StansfieldMasoud BaratiBen Van LierMasoud MirtaheriBen WitvlietMasoud Nazarian-SamaniBen WolfMassimiliano BenettiBence Tamás SzabóMassimiliano BordoniBenedetto BarabinoMassimiliano ComissoBenedict McLarneyMassimiliano DonatiBenedikt BiererMassimiliano GardaBenish FidaMassimiliano PastenaBenjamin L. MillerMassimiliano PepeBenjamin MentiplayMassimo CapocciaBenjamin MetcalfeMassimo ConfortiBenjamin MorrellMassimo DonelliBenjamin TurnbullMassimo FabrisBennett I BertenthalMassimo FiccoBennett LambertMassimo MarcaccioBennie Ten HakenMassimo MontisciBenoit CharbonnierMassimo MusacchioBenoit HilloulinMassimo OliveroBenoît IgneMassimo PetrarcaBenoît ParreinMassimo PieriBenoit PicouxMassimo SalviBenoît PirandaMassimo VivianiBenoit PiroMassimo Walter RivoltaBenpeng ZhuMassimo ZuccoBeomseok OhMasudul Haider ImtiazBerend Jan Van Der ZwaagMasudul ImtiazBeril SirmaçekMat DalgleishBernadette DorizziMatea ZajcBernard B. MunyazikwiyeMatej MencingerBernard KontnyMatej NjegovecBernard PottierMatej SupejBernard SchmidtMateja KlunBernard UguenMateo BurgosBernardo B. GattoMateus MendesBernardo WagnerMateusz AndrychowiczBernd BaumannMateusz BawajBernd J. StetterMateusz CzyzyckiBernd-Christian RennerMateusz DybkowskiBernhard J. HoendersMateusz KorpyśBernhard KoelmelMateusz KuklaBernhard ManhartsgruberMateusz MalanowskiBernhard PraherMateusz NowakBernhard Rainer TittmannMateusz PaligaBernhard SchmaussMateusz PasternakBernhard ZagarMateusz RzymoeskiBert DriessenMateusz SalachBerta Baca-BocanegraMateusz ZawadzkiBerthold HornMatheus Silva GonçalvesBertram TaetzMathew G. PelletierBertrand DavidMathew L. WymoreBertrand SchneiderMathew LeggBertrand SimonMathias BlandeauBeth SmithMathias ForjanBethel OsuagwuMathias LemmensBettina DebûMathieu DomenjoudBezerianos AnastasiosMathieu EtienneBhaben KalitaMathieu GraveyBhagya SamarakoonMatias Garcia-ConstantinoBharanidharan ShanmugamMatija JezeršekBharatesh ChakravarthiMatija KrznarBharath Babu NunnaMatija MilanicBharath RameshMatjaz SramlBhimsen RoutMatko ŠarićBhogendra MishraMatloob KhushiBiagio CarboniMatrella GuidoBiagio RaponeMatteo AneddaBian TianMatteo BolcatoBian WuMatteo BordoniBianchi MeiguinsMatteo BottinBianchini CarloMatteo Bruno LodiBiao HuMatteo Claudio PalpacelliBiao KongMatteo CortesiBiao YangMatteo CristaniBifeng HuMatteo Del SoldatoBih-Chyun YehMatteo LupertoBikram BanerjeeMatteo ManganelliBilal AsadMatteo MartucciBilal H. AlsalamMatteo MorettiBilal HussainMatteo RiccòBilal KhanMatteo SaverianoBiliana GeorgievaMatteo ScaramuzzaBin ChenMatteo SensiBin ChengMatteo SignorileBin GaoMatteo StrozziBin LiMatteo ValtBin ShenMatthew A. CooperBin ShengMatthew B. CoppockBin ShiMatthew B. MyersBin SongMatthew BrandsemaBin WangMatthew ConleyBin WuMatthew EadyBin YangMatthew GaddBin YinMatthew HobbsBin ZhangMatthew J. StainerBinbin MiMatthew PainBinbin YangMatthew PikeBinchun LuMatthew ScottBineng ZhongMatthew VealeBing LiMatthew WorseyBing SheMatthias DümpelmannBing SunMatthias RädleBing WangMatthieu N. BooneBing YuanMatti HuotariBing ZhuMattia BorgarinoBingbing GaoMattia BrambillaBingfei FanMattia ZorziBingfeng PanMattias DahlBingkun LuoMattias O’NilsBingpu ZhouMatúš PlevaBingqing LuoMaura PavesiBingrong MiaoMaurice G. CoxBingrui ChenMaurice MohrBingwei HeMaurice Van KeulenBinh Q. TranMauricio CerdaBinsheng ZhangMauricio David PerezBinxin LiuMauricio J. SilvaBiplab PaulMaurício Pamplona SegundoBirgitta Dresp-LangleyMauricio PorathBishnu RegmiMaurizio CampoloBiswajit PadhyMaurizio MagariniBiswajit SarkarMaurizio NaldiBiswanath DuttaMaurizio SpadavecchiaBivas PanigrahiMauro BelloneBjørn JægerMauro CaccavaleBjörn KrügerMauro Callejas CuervoBjørn Olav HogstadMauro De FeudisBjørn SkallerudMauro EpifaniBlanca Tejedor HerranMauro FaddaBlanka TundysMauro Fazion FilhoBlaž PodgorelecMauro FelizianiBlaž ŠkrljMauro FemminellaBo ChenMauro GaspariBo DaiMauro LeonardiBo FangMauro Lo BruttoBo HuangMauro MariottiBo LiMauro PaganoBo LiuMauro Sebastián InnocenteBo MaoMauro SodiniBo PeiMauro TropeaBo TanMavroulis SpyridonBo WangMax M. NorthBo WuMax Mauro Dias SantosBo YangMax Ortiz-CatalanBo YuMax RyadnovBo ZhangMaxim BakaevBo ZhengMaxim DulebenetsBobak MortazaviMaxim E. DarvinBocai GaoMaxim IvanovBochen ZhangMaxim KalininBocheng ZhuMaxim KhomenkoBodor MariusMaxim V. RyzhiiBofan SongMaxime Alex Junior KuitcheBofeng GuoMaxime BourgainBogdan Cătălin ȘerbanMaxime MoreaudBogdan Constantin NeaguMaxime MouyenBogdan Cristian FloreaMaximilian NicolaeBogdan DziadakMaximo CobosBogdan FirtatMaximo Morales-CespedesBogdan Florin FilipMay Portuguez CastroBogdan GhermanMaya DimitrovaBogdan GilevMayukh BhattacharyyaBogdan GrozaMayuresh Sunil PardeshiBogdan IancuMaziar NekoveeBogdan LipušMd Alamgir HossainBogdan OrzaMd. Al-Amin KhandakerBogdan PankiewiczMd AsikuzzamanBogdan RǎducanuMd Belal Bin HeyatBogdan SłodkiMd Eshrat E AlahiBogdan SoceaMd. Faruk HossainBogdan StrimbuMd. Humayun KabirBogdan TiganoaiaMd Juber RahmanBogdan ZagajewskiMd Junayed HasanBogdan-Constantin NeaguMd Mahbubur RahmanBoggarapu Praphulla ChandraMd Mominul AhsanBogusław CyganekMd Mostafa Kamal SarkerBogusław SzlachetkoMd Noor-A-RahimBogusława KwoczyńskaMd. Sanaul Haque MondalBo-Hao ChenMd ShamsuddohaBohdan RusynMd Sipon MiahBoitumelo MatsosoMd Tanvir HasanBojan MilovanovićMd Zafar IqbalBojan StumbergerMed Aymen ChalloufBolin ChenMehanathan PathmanathanBo-Lin JianMehdi A J Van Den BosBongsoon KangMehdi AddaBon-Gwan GuMehdi BoukallelBooma Sowkarthiga BalasubramaniMehdi BoukhechbaBoonaert JacquesMehdi EshaghBordel SánchezMehdi FoumaniBoris BačićMehdi GhoreyshiBoris DzantievMehdi HosseinzadehBoris G. VainerMehdi NosratiBoris GinzburgMehdi PazhooheshBoris Igor PalellaMehdi PirahandehBoris KudryashovMehdi RahmatiBoris LuninMehdi RoozmehBoris Mikhailovich ShumilovMehdi SalimiBoris MillerMehedi MasudBoris N. FedulovMehmet Alp IlgazBorislav DimitrovMehmet KarakoseBorislav StoyanovMehmet OgutBorivoje PašićMehmet SenelBorja Bordel SánchezMehran BehjatiBorja Espejo-GarcíaMehran MehrandezhBorja Genovés GuzmánMehran OraeeBorja PeleatoMehrmohammadi MohammadBorna AbramovićMehtab SinghBor-Ran LiMei PanBor-Shing LinMei SangBor-Shyh LinMeicheng LiBosheng QinMeikang HanBostjan BatageljMeimei LaiBoštjan KovačićMeir NitzanBoštjan MarkoliMeiwei KongBoštjan ŠumakMeizhen WangBotond Sandor KireiMel SuffetBoudewijn Van LeeuwenMelania SusiBousoulas PanagiotisMelania TeraBowei DongMelanie EliasBowen GongMelanie Haehnel-TaguchiBowen ZhuMelina P. IoannidouBowu ZhangMelina-Maria ZempilaBoxiang WangMelinda DavidBoxuan ZhongMelisa Del BarrioBoyang LiMelissa M. Rodríguez-DelgadoBoyd FowlerMelkie TadesseBozena BorowskaMeltem ElitasBożena HołaMeltem YanilmazBożena JarząbekMelvin DiazBozhao QiMeng GaoBožidar PotočnikMeng HanBrach PostonMeng LiBrad McDanelMeng LinBraden M. LiMeng ZhouBradley EdelmanMengbao FanBrain ViaMengchu ZhouBram VanderborghtMengfang LiBraml ThomasMeng-Fang LinBrandon FoubertMenghan HuBranka StojanovicMengistie KinduBranko BabusiakMengjie HanBranko KolaricMengjie ShiBrayan S. Zapata-ImpataMeng-Kun (Jason) LiuBrendan HeeryMenglan DuanBrett A. StoryMeng-Leong HowBrett BorghettiMenglong LiuBrett HamblyMengmi ZhangBrian ArgrowMengyan NieBrian BellMeng-Yun ChungBrian BernardMeonghun LeeBrian BirchMercedes AyusoBrian BrownMercedes Del Río CelestinoBrian ButkaMercedes RuizBrian HooverMercedes SollaBrian J. WisnerMerryn ColeBrian N. KimMert SevilBrian OlshanskyMerve KeskinBrian RockMeysam KeshavarzBrian VestalMezgeen RasolBrian Y. TsuiMhafuzul IslamBrice GrunertMhamed SayyouriBrigid Betz-StableinMi ChenBrik BouzianeMia FolkeBriliant Adhi PrabowoMiao HuBritt ÖstlundMiao LuBronisław StecMiao YuBrook GalnaMiaoqiang LyuBruce BernackiMiaowen WenBruce HaycockMicael NascimentoBruce StephenMicaela MorettiniBruce T. TsurutaniMicaela SchmidBruno Albuquerque De CastroMichael A. CowlingBruno AndòMichael A. LombardiBruno BrunoneMichael ArandBruno CaillierMichael BaddeleyBruno De Carvalho AlbertiniMichael BalchanosBruno FerreiraMichael BaldaufBruno FiondaMichael BarzBruno José Nievas SorianoMichael BorichBruno MärklMichael CoughlinBruno MasieroMichael D. GrayBruno O.S. TeixeiraMichael DeGiorgioBruno SilvaMichael DöllingerBruno SousaMichael DomingueBruno VolckaertMichael EllisonBruno WacogneMichael FarmerBruno WatierMichael FowlerBugra AlkanMichael FriebeBugra AyanMichael G. MaukBurak AlakentMichael GarstangBurcu GumuscuMichael GouldBurkhard WrengerMichael H. F. WilkinsonBushra JalilMichael HahnBushra ZamanMichael HauptBuyun ShengMichael HeiseByeong Ha LeeMichael HewsonByeong Hee KimMichael HöftByeong-Choon GooMichael I. OjovanByeongil KimMichael J. AbramsByoung Hun LeeMichael J. WoutersByoungChul KoMichael James PriceByoung-Hee LeeMichael KirchhoffBystrík DolníkMichael KuntzschByung Gwan HyunMichael L. MyrickByung Yong JeongMichael LataByung-Cheol KimMichael MacDonaldByung-Gyu KimMichael MangoldByung-Gyu YuMichael MarxByung-hoon KimMichael McGuireByunghun LeeMichael MeyerByung-Kuk SeoMichael MielewczikByung-Kwen SongMichael MortimerByungseok YooMichael MückByungTae OhMichael MuehlberghuberByungwook KimMichael MunzByung-Wook ParkMichael NikolaouByungwoon ParkMichael O’ByrneC. Ahamed SaleelMichael OsadebeyCaetano Mazzoni RanieriMichael O’TooleCai LiuMichael P. CaligiuriCai LuoMichael P. CravenCairo L. Nascimento, Jr.Michael R. KoblischkaCaisheng WeiMichael Reader-HarrisCaitlyn MurphyMichael RisticCaiwei ShenMichael SakellariouCaleb RasconMichael SemenovCalin CiufudeanMichael ShribakCalin CorciovaMichael ThompsonCălin VlădeanuMichael TrevinoCallum A. WalterMichael VannierCalvin EiberMichael WermanCamelia AvramMichael WinokurCamelia CerbuMichael Yu KataevCameron ParviniMichaela BalažikováCamila MG GussenMichaela ServiCamila P. SalomonMichail GianniouCamilla KärnfeltMichail J. BeliatisCamillo MarianiMichail KlontzasCamillo ResslMichail-Alexandros KourtisCamilo DíazMichal BalaziaCan HuangMichal BoreckiCan KoralMichał BrachCan KoyuncuMichal DolezelCàndid ReigMichal DudekCandido DuarteMichał FularzCanek PortilloMichal GálaCaner SahinMichał GinsztCanicious AbeynayakeMichal HolubCanjun YangMichal HuptychCao-An VuMichał JózwikCapuano RosamariaMichał JurekCarina Barbosa PereiraMichal KaczmarekCarina HahnMichal KebisekCarl James DebonoMichal KelemenCarl MalingsMichał KunickiCarl StrathearnMichal KvetCarla QueirosMichał LandowskiCarlo BaldisserriMichał MorawskiCarlo BongioanniMichał NowickiCarlo CastellanoMichał PająkCarlo DossiMichal PiaseckiCarlo DragoMichal PodporaCarlo Giacomo LeoMichał R. NowickiCarlo GiglioMichał R. WróbelCarlo Iapige De GaetaniMichał RogalaCarlo MassaroniMichał RomaszewskiCarlo MolardiMichal SchmirlerCarlo MorassoMichał ŚmiejaCarlo RainieriMichal StrzeleckiCarlo Requião Da CunhaMichal SzermerCarlo RicciardiMichal WieczorowskiCarlo TiebeMichał WojasińskiCarlo TiseoMichal WydraCarlos A. CifuentesMichalina BłażkiewiczCarlos A. P. SoaresMichalis A. SavelonasCarlos AbreuMichalis KassinopoulosCarlos Alberto Ferreira MarquesMichalis P. MichaelidesCarlos Alex Sander J. GuloMichalis SavelonasCarlos Andres Lara-NinoMichalis VrigkasCarlos Andres Luna VázquezMichalski AndrzejCarlos CapovillaMichel Eustáquio Dantas ChavesCarlos Carbonell CarreraMichel GayCarlos CarvalhoMichel JourlinCarlos CasqueiroMichel KasserCarlos Couder-CastañedaMichel MissonCarlos D. Garcia-BeltranMichel NielenCarlos D. Moreno-MorenoMichel PaindavoineCarlos DuarteMichel RiguidelCarlos E. Galván-TejadaMichel SalomonCarlos Eduardo Sanches De AndradeMichela BorghettiCarlos Eduardo ThomazMichela GoffredoCarlos EscobedoMichela QuadriniCarlos Fernandez-BassoMichelangelo GuaitoliniCarlos G. JuanMichele Arturo CaponeroCarlos García-RubioMichele BoffanoCarlos GriloMichele CalìCarlos GuindelMichele CarboniCarlos Gustavo Resque Dos SantosMichele ColajanniCarlos GutierrezMichele D’AttilioCarlos HernandoMichele De SantisCarlos IturrinoMichele DeiCarlos J. EscuderoMichele GirolamiCarlos Javier Sosa GonzálezMichèle GouiffèsCarlos LaranjeiraMichele MangiameliCarlos Lopez ArdaoMichele MastroianniCarlos M. MateoMichele OrtolaniCarlos M. Travieso-GonzalezMichele PisanteCarlos MarquesMichele RoccotelliCarlos MarquezMichele ScandolaCarlos MedranoMichelle LiouCarlos Moreno-GarcíaMichelle MellenthinCarlos MoronMichio NiwanoCarlos Muñoz-PobleteMigallón HéctorCarlos Oscar Sanchez SorzanoMiguel A. Jaramillo-MoránCarlos PascalMiguel A. SantoyoCarlos Perez-RamirezMiguel Ángel Caminero TorijaCarlos PfeifferMiguel Ángel Fernández TorresCarlos PlateroMiguel Angel Fernandez-GraneroCarlos R. Del-BlancoMiguel Ángel Gómez RuanoCarlos R. MichelMiguél Ángel GutierrezCarlos Renato MenegattiMiguel Ángel LópezCarlos Rizo-MaestreMiguel Angel Luque-NietoCarlos Roberto Hall BarbosaMiguel Ángel Maté GonzálezCarlos Roberto MinussiMiguel Ángel Naya VillaverdeCarlos Roldán-BlayMiguel Ángel SatorreCarlos Romero MoralesMiguel CarrascoCarlos RosadoMiguel Castelo-Branc SousaCarlos SennaMiguel ChavarríasCarlos SerodioMiguel Clavijo JiménezCarlos SerrãoMiguel Díaz-RodríguezCarlos SilvaMiguel Dovale AlvarezCarlos Soubervielle-MontalvoMiguel Franklin De CastroCarlos Torres-TorresMiguel Gabriel Villarreal-CervantesCarlos Travieso-GonzálezMiguel García-PinedaCarlos VazMiguel León-RodríguezCarlos ZafraMiguel Martínez GarcíaCarlos-Alberto Cruz-VillarMiguel Maté-GonzálezCarlos-D. Martínez-HinarejosMiguel Murillo-EscobarCarme CarrionMiguel NavarreteCarmelo CorsaroMiguel OssandonCarmelo MilitelloMiguel Pic AguilarCarmelo MineoMiguel R. Oliveira PanãoCarmelo SferrazzaMiguel Reis E. SilvaCarmen BisogniMiguel Rodríguez PérezCarmen Cristófol RodríguezMiguel Torre GarcíaCarmen DelgadoMiguel TorresCarmen JarenMiguel-Angel Luque-NietoCarmen Moret-TatayMiha AmbrožCarmen Peláez-MorenoMiha RavberCarmen ProMihael MakekCarmen VoicuMihael MohorcicCarmine CiofiMihaela AndreiCarmine MaffeiMihaela BacalumCarol CorsiMihaela Badea DoniCarol HabibMihaela BalanescuCarol MaherMihaela ColhonCarolina Del-Valle-SotoMihaela CretuCaroline HartleyMihaela GheorghiuCaroline KönigMihaela HnatiuCarrillo Dan GarcíaMihaela Ioana BaritzCarson LeungMihaela OleksikCarsten BehnMihaela PuiuCarsten DoscheMihaela TertişCarsten LaukampMihaela-Ligia UnguresanCasey ChowMihai Ciprian MărgărintCaspar ClarkMihai CiucCasper Boongaling AgatonMihai CrenganisCassio PaivaMihai GavrilasCastillo-Rivera SalvadorMihai Olimpiu TatarCataldo DoriaMihai SanduleanuCataldo GuaragnellaMihai UdrescuCatalin AlexandruMihai Victor PricopCatalin AmzaMihai ZahariaCătălin BuiuMihaiela IliescuCatalin Daniel CaleanuMihail AbrudeanCatalin I. PruncuMihail IancoviciCatalin PetrescuMihail MinescuCatalin PruncuMihalj BakatorCatalin StoeanMikael ForsmanCatalina Rus-CasasMikael LuthjeCatalina-Alice BrandusMikail F. LumentutCatalina-Lucia CocianuMikalai M. BudzevichCatalin-Iosif CionteaMike AdamsCatarina Ferreira Da SilvaMike MyersCatarina ReisMike Oluwatayo OjoCate MadillMikel EmaldiCaterina FedeMikel Ogueta-GutiérrezCathal HeaveyMikhail BasarabCatherine E. LangMikhail BasovCatherine GroganMikhail BeklemishevCátia MagroMikhail BelovolovCatia PrandiMikhail BogachevCecilia ClivatiMikhail Evgen’evich ProkhorovCecilia CristeaMikhail KirillinCecilia Di RubertoMikhail KomarovCecilia JimenezMikhail KrinitskiyCecilia Jimenez-JorqueraMikhail LytaevCecília Sik-LányiMikhail P. KozochkinCecilia SuraceMikhail SkvortsovCedric CaruanaMikhail SlobodyanCedric Le GentilMikhail TarkovCelestine IwendiMikhail ZolotukhinCem DirekoğluMikio DeguchiCen TangMikko PeltokangasCenek SasinkaMiklos FriedCengiz KoparanMikolaj BuchwaldCésar Benavente PecesMikołaj KarpińskiCésar Calvo-LoboMikuláš HubaCesar CameriniMilad GholamiCésar CárdenasMilad HeydariaanCesar CollazosMilad KamkarCesar Da CostaMilad KarimshoushtariCésar Elosúa AguadoMilan ČervenkaCésar Fernández-SánchezMilan DurdánCésar GattanoMilan DžundaCésar Llamas BelloMilan GnjatovićCésar Parcero-OubiñaMilan PolivkaCésar Ricardo Soto-OcampoMilan SmetanaCésar Yutaka OfuchiMilan SysCesare Di Girolamo-NetoMilan TomaCesare ValentiMiled AmineCezary BehrendtMilena B. ČukićCezary KowalczykMilena Faria PintoCezary OrłowskiMilena MarkovićCezary ZiółkowskiMiles GraftonChadd W. ClaryMilica JankovicChadin KulsingMiloš MilovanovićChadrasekhar LokaMiltiadis KandiasChae Eun RheeMilto MiltiadouChae-Bong SohnMilton Hirokazu ShimabukuroChah KarimaMilton PereiraChaitanya Kumar SuddapalliMiltos KyriakidisChakchai So-InMilutin RadonjicChakfong CheangMimi ReckerChan H. SeeMin KimChan Hwang SeeMin LiChan Su LeeMin PanChance TarverMin ParkChandan DeMin Seok MoonChandrama SarkerMin TuChandrasekaran JayaramanMin XiaChandreswar MahataMin XieChang Hong LinMin XuChang Kyu JeongMina SamukawaChang LiMina SheikhalishahiChang LiuMinas DasygenisChang PengMinas PatsalidesChang WangMinchae JungChang Won LeeMindy LevineChang Wu YuMindy S. LevineChangbao WenMinfang YehChanggil LeeMing LiuChangho LeeMing Tzer LinChangho YunMing YanChanghong WangMing YangChanghong YoumMing YuChang-Hoon ChoiMing YuanChanghoon LeeMing-Bao HuangChanghua ZhuMing-Chang PaiChangjiang KouMingchao LiChangqing CaoMingchen ZhugeChang-Sei KimMing-Chin ChuangChang-Seuk LeeMingda ZhouChangsheng DaiMingde DuChang-Soo LeeMingder YangChang-Wei HsiehMing-Feng GeChangwon KimMing-Fong TsaiChangyeun MoMinghsun WuChangyong CaoMinghui LiChangyong YimMing-Hung HsuChangyu LiuMing-Hung WangChang-yue ChiangMing-Jer JengChangzhao LiuMing-Jie SunChanjun JeonMingjie YinChanKyu KangMingjie ZhangChanon NgamsombatMingkui WuChansik ParkMingliang ZhouChansu YangMingmin ZhaoChan-Won ParkMingsen PanChanwoo MoonMingsheng MaChao ChenMingshun JiangChao FuMing-Shyan WangChao HuMingsong WangChao HuangMingsu SiChao LiuMing-wei HsuChao LuMingxuan ChenChao MiMingyang LuChao SunMing-Yang SuChao TanMingye JuChao WangMing-Yen HsiaoChao WuMingyu LimChao XuMingyuan YanChao ZhaiMingzhang ChenChaochen GuMingzhe JiangChaoching HoMingzhe ZhangChao-Chung PengMin-Ho LeeChaofan MaMinhong SunChaofang ZhaoMinh-Quang TranChaofeng WangMinh-Tu Cao (Luis) Chaofeng YeMinjeong HaChao-Hsien LeeMinjie GuoChaojun TangMinjoong RimChaoqun WangMin-Jung KangChaoqun XiangMinkoo KimChaoran LiuMin-Kook ChoiChao-Sung LaiMinkyu AhnChaotan SimaMinkyu SongChao-Tung YangMinna LanzChaouki HannachiMino SportelliChaoxing LiuMinoru SasakiChaoyang GongMinseok ChoiChaoyang JiangMin-Seok KangChao-Yang LeeMinshui HuangChaoyang TiMinvydas RagulskisChaoyang ZhangMinxiang ZengChaoyuan LinMiquel Sànchez-MarrèChaozhe JiangMira NaftalyCharalambos SergiouMiralda CukaCharalampos DimoulasMiran LeeCharalampos GeorgiadisMiran MerharCharalampos KalalasMiran PobarCharalampos KonstantopoulosMircea Cristian DudescuCharalampos N. PitasMircea Dorin VasilescuCharalampos OrfanidisMircea DulăuCharalampos ValsamosMircea HuleaCharalampos Z. PatrikakisMircea IvanescuCharilaos AkasiadisMircea NeagoeCharilaos C. ZarakovitisMircea Paul MuresanCharis NtakoliaMircea VladutiuCharles CarlsonMircea-Bogdan RadacCharles F. BabbsMirco MuttilloCharles KnightMirco SturariCharles MadewellMirela CatalinaCharles PontonnierMirela FrandesCharles SackettMirella De SistoCharles SammutMirHojjat SeyediCharles Sebiyo BatchoMiriam A. Carlos-MancillaCharlie CatlettMiriam ZachsenhouseCharlotte LawsonMirja TenhunenChase ShimminMirjana Pejić BachChathura AbeywickramaMirko De MelisChathuranga KumarageMirko MarrasChatzichristofis SavvasMirko MazzoleniChau YuenMirko MustonenChee Kiat SeowMirko PoljakChee Wei AngMirko ViroliChelladurai KaruppiahMirna Habuda-StanićChelsea DunningMirna ŽulecChe-Ming LiMiron WeinrebChen CaoMiroslav BuresChen ChenMiroslav CísarChen Chin-LingMiroslav JůzlChen ShiMiroslav KulichChen WangMiroslav PástorChen XingMiroslav PohankaChen YangMiroslav PokornýChen ZhaoMiroslav VoznakChen ZhuMiroslav ŽivićChenchu XuMiroslava NedyalkovaCheng ChenMiroslaw MazurekCheng ChiMirosław NejmanCheng HuMirosław PajorCheng Siong ChinMiroslaw SwierczCheng SongMirosław SzmajdaCheng TuMiroslaw WitosCheng WuMirza PojskićCheng XuMirza SarajlicCheng YanMisa HayashidaCheng ZongMislav StepinacChengcai LengMitch PryorCheng-Chi LeeMitchell L. NeilsenCheng-Deng KuoMitchell WelchCheng-Hsien LiuMithun Kuniyil Ajith SinghCheng-Hsiung HsiehMithun MukherjeeCheng-Huan ChenMitja TruntičCheng-Hung ChuangMitra SubhabrataCheng-Hung LinMitrea Delia-AlexandrinaChengjin QinMitsugu HasegawaChengkuo LeeMitsuhiro ShigeishiCheng-Liang HuangMittal MohitCheng-Liang WangMizanoor RahmanChenglong ShaoMizuho NishioChengming SunMladen AmovićChengqing LiMladen JurišićChengqing LiuMladen ZrinjskiCheng-Shiu ChungMłynarczyk PrzemysławCheng-Te LinMM Manjurul IslamCheng-Ting HsuMo ZhaoCheng-Wei FeiMoacir Moacir GodoyChengwei ZhouMoayad AloqailyChengxi LiMobayode AkinsoluChengxi ZhangMocanu IrinaChengyong LiMochimitsu KomoriCheng-Yuan ChangModestos StavrakisChengyuan DongModris GreitansChengyuan LiuMogalahalli V. ReddyCheng-Zhi QinMohamad AlzayedChenhao ChiuMohamad AwadChen-Kun TsungMohamad SawanChensheng WuMohamadali MalakoutianChenshuang LiMohamed Abd ElazizChen-Ting LiaoMohamed Abdel-NasserChen-Wei ChenMohamed AbdelrahemChen-wen YenMohamed Amine FerragChenxi YangMohamed Atef Abbas ElsahartyChenxing WangMohamed Ben Haj FrejChenyu HuangMohamed BenbouzidCheol Gi KimMohamed BouazaouiCheol SongMohamed BoutaayamouCheol-Hong HwangMohamed DahmaneCheol-Hwan YouMohamed DarwishCheolsoo ParkMohamed E. TamazinCheon Won ChoiMohamed ElhabibyCheonyong KimMohamed El-HadidyChereches Nelu CristianMohamed FnadiCherlyn J NgMohamed Foysol ChowdhuryChetna GuptaMohamed HamdaouiChe-Wei LinMohamed HammadChi Bum AhnMohamed SaadChi Cuong VuMohamed SalahudeenChi Hou LeiMohamed SerryChi LeeMohamed SharafeldinChi ZhangMohamed ShehadehChia-Chen ChangMohamed ShehataChia-Chi ChengMohamed TamazinChia-Chin ChiangMohamed Wiem MkaouerChiachung ChenMohamed ZaghloulChia-Hsien FengMohammad A. AljamalChia-Hui LiuMohammad A. JaradatChia-Hung Dylan TsaiMohammad A. MaktoomiChia-Min YangMohammad AlibakhshikenariChia-Ming ChangMohammad Bagher DowlatshahiChiao-Chi LinMohammad BelalChiara AllegrettiMohammad Faridul Haque SiddiquiChiara BedonMohammad H. AlomariChiara BersaniMohammad H. Nadimi-ShahrakiChiara BertolinMohammad HammoudehChiara CeccariniMohammad HarbChiara GaribottoMohammad Hossein AmirhosseiniChiara GrudenMohammad Iman Mokhlespour EsfahaniChiara IacovelliMohammad JafariChiara MocenniMohammad JamshidiChiara PortesiMohammad Jobayer HossainChiara RomeiMohammad KakooeiChiara SorberaMohammad Kamrul HasanChia-Tai ChanMohammad KhisheChia-Wei TsaiMohammad Khubeb SiddiquiChia-Yeh HsiehMohammad Mahdi Rezapour MashhadiChi-Ching ChangMohammad Mahdi TajikiChi-Ching KuoMohammad Mehdi RashidiChichung CheungMohammad Mehedi HasanChien-Chang ChenMohammad MiralinaghiChien-Cheng JungMohammad MoghimiChien-Chung ShihMohammad NadimiChien-Hung LiMohammad NajafzadehChien-Hung LiaoMohammad NooriChien-Nan KuoMohammad Reza JabbarpourChien-Sheng ChenMohammad Reza KarafiChien-Sheng LiuMohammad Reza KhosravaniChien-Tai HongMohammad Reza MosaviChien-Yao WangMohammad Reza SalamiChih Chiang HongMohammad RmaytiChih Jer LinMohammad RostamiChih-Chang YuMohammad Saeid GhaffarianChih-Chen YihMohammad SafeeaChih-Chieh LiuMohammad ShojafarChih-Chieh YangMohammad Shokrolah ShiraziChih-Chin YangMohammad SoleymaniChih-Hao LeeMohammad TaghinejadChih-Hong LinMohammad Tavakkoli YarakiChih-Hsien HuangMohammad ValipourChih-Hsiung ShenMohammad WazidChih-Hung WangMohammad Yazdani AsramiChi-Hieu PhamMohammadali GhafarianChih-Peng FanMohammadreza ShokouhimehrChih-Ping LinMohammadreza SoltaninejadChih-Sheng HuangMohammed A. A. Al-qanessChi-Hua ChenMohammed AbuhamadChi-Hung HwangMohammed Al SadsoonChih-Wei ChiuMohammed AlhaddadChihwen HsuehMohammed Amin AlmaiahChih-Yang LinMohammed AwadChih-Yu KuoMohammed BahjaChih-Yu WangMohammed BazChih-Yu WenMohammed ChadliChih-Yung HuangMohammed DiykhChi-Jie LuMohammed ElmogyChikahiro ImashiroMohammed H. AlsharifChi-Kuei WangMohammed IjazChiman KwanMohammed MahmoudChin-Chi ChengMohammed Salah Al-RadhiChin-Chun TsaiMohammed Salih Mohammed GismallaChin-Feng LinMohand Arab DjeziriChing Biau TzengMohataz HossainChing Torng LinMohd Anuaruddin Bin AhmadonChing-Chi HsuMohd Azizi Abdul RahmanChing-Chou WuMohd Noor IslamChing-Hsin WangMohd Saiful Azimi Bin MahmudChing-Hung LeeMohd Shahrimie Mohd AsaariChing-Jer HuangMohit MittalChing-long ShihMohit SarafChing-Lung ChangMohiuddin AhmedChing-Mu ChenMohsen AsadniaChingShun LinMohsen AzarmiChing-Ta LuMohsen KaboliChin-Jen LinMohsen KoohestaniChin-Shiuh ShiehMohsen RanjbaranChinthaka PremachandraMohsen RezvaniChiou-Jye HuangMohsin JamilChiou-Shann FuhMoi Hoon YapChiranjibi SitaulaMoin U. AtiqueChiu C. TanMoinak MaitiChiuan-Chian ChiouMojca UrbancicChiu-Keng LaiMojtaba Ahmadieh KhanesarChiu-kuo LiangMojtaba ForghaniChi-Wai ChowMojtaba GhodsiChi-Wen LungMojtaba Jafari TadiChi-Yi TsaiMojtaba JoodakiChi-Ying LinMojtaba KhanesarChi-Yuk ChiuMojtaba NoghabaeiCho Nilar PhyoMojtaba ZeraatpishehChokri SendiMolnár BenceChong Hyun LeeMolong DuanChong LeiMona IonasChongdeuk LeeMona JaberChongwen HuangMonica Ballesta GaldeanoChongyi FanMonica BettaChoongsoo ShinMónica Fernández BarcielaChoong-un KimMonica FigueiredoChou Ching LinMonica FlorescuChouikhi SamiraMonica LöfvanderChouki SentouhMonica MariniChou-Yuan LeeMonica NecchiChris DritselisMonica PătrașcuChris H. BahnsenMónica PintoChris HepplewhiteMonica SirouxChris JoslinMonica TiboniChris McGibbonMonika BłaszczyszynChris RhodesMonika Cieslikiewicz-BouetChrister JohanssonMonika GelkerChristiaan Etienne VermeulenMonika Gwóźdź-LasońChristian A. CousinMonika SobiechChristian BeckerMonssef Drissi-HabtiChristian Bernhard KuttnerMonsur IslamChristian Bräuer-BurchardtMontañes Rosa RodriguezChristian Cabrera JojoaMontserrat CallejaChristian CordaMoon Il KimChristian DobelMoon K. KwakChristian EitzingerMoon-Kyu LeeChristian Fernández-CampusanoMoonseong KimChristian G. Quintero M.Moonsoo KangChristian GaleaMoreno D’AmicoChristian GianoglioMorgan FunderburkChristian KehlMoritz HessChristian KrambergerMoritz S. SchmidChristian KuttnerMorshed ChowdhuryChristian Mata MiquelMorteza BabaieChristian MesterMorteza GhorbaniChristian NapoliMoshe GaiChristian PehamMoshe Rav AchaChristian QuadriMostafa ElgendyChristian RummeyMostafa Ghobaei-AraniChristian SalimMostafa HaghiChristian SantschiMostafa HassanalianChristian ThomMostafa HusseinChristian VanhilleMostafa Kamal MasudChristian ViehwegerMostafa NabawyChristian VogeleyMostafa SadeghiChristian WernerMostefa Mohamed-SeghirChristian-Alexander BungeMotoaki HaraChristiane ThielemannMotoya SuzukiChristina KakderiMouad LemouddenChristina KellerMoussa HamadacheChristina RöckeMoustafa HusseinChristina Salchow-HömmenMrinal MandalChristina SiontorouM.S. ZobaerChristina StreliMu-chun WangChristine DetrembleurMueen UddinChristine DewiMufti MahmudChristine GabardoMugahed A. Al-antariChristine KallmayerMuguang WangChristine LetrouMuhammad Aamir CheemaChristodoulos KelirisMuhammad Abdul QyyumChristoffer R. HeckmanMuhammad AfaqChristoforos NtantogianMuhammad AfzalChristoph BayerMuhammad AhmadChristoph BodeMuhammad Ahsan SaeedChristoph HartmannMuhammad Ali ButtChristoph HintermüllerMuhammad Amir KhanChristoph Hoog AntinkMuhammad Amir ShafiqChristoph KralovecMuhammad AmithChristoph RascheMuhammad AnshariChristoph ReichMuhammad Arif MahmoodChristoph ReichertMuhammad AsadChristoph RitterMuhammad Asghar KhanChristoph StachMuhammad Ashfaq JiskaniChristoph Steup Muhammad Ashfaq KhanChristoph TholenMuhammad Asif Zahoor RajaChristoph WasserzierMuhammad AslamChristophe DelebarreMuhammad AttiqueChristophe JallaisMuhammad Attique KhanChristophe KolskiMuhammad AwaisChristophe LohrMuhammad AzizChristophe RosenbergerMuhammad AzmatChristophe VoisinMuhammad Ehatisham-ul-HaqChristopher A. G. KalninsMuhammad Fahad ZiaChristopher AdcockMuhammad FaheemChristopher BaerMuhammad Fazal IjazChristopher BallmannMuhammad Hassan KhanChristopher BeachMuhammad HussainChristopher BystroffMuhammad IkramChristopher ChesherMuhammad KashifChristopher DicesareMuhammad Kashif ShahidChristopher G HealeyMuhammad Khalil AfzalChristopher G. WilsonMuhammad Mahmood AliChristopher HolmesMuhammad NadeemChristopher KentMuhammad Naveed AmanChristopher PaoliniMuhammad Naveed Aman AmanChristopher StewartMuhammad NazirChristopher T. GoodinMuhammad Raheel BhuttaChristos AlexakosMuhammad RashidChristos DouligerisMuhammad Sadiq FareedChristos IoakeimidisMuhammad Saif Ullah KhalidChristos K. MichailMuhammad Salman Al FarisiChristos KokkinosMuhammad Salman HaleemChristos KoulamasMuhammad ShafiqChristos L. StergiouMuhammad Shuja KhanChristos MichailMuhammad Usman HadiChristos MourtziosMuhammad Usman YounusChristos NikolopoulosMuhammad Yousuf Irfan ZiaChristos P. AntonopoulosMuhammad ZubairChristos PappasMuhammd Mudassar YaminChristos RahiotisMuhammed Said BoybayChristos RiziotisMuharfiza MuharfizaChristos S. AntonopoulosMuharrem Kemal ÖzfıratChristos SpandonidisMuhib RahmanChristos TjortjisMuhidul Islam KhanChristos TroussasMujtahid KaavessinaChristos TseliosMukesh KumarChristos VerikoukisMukesh Kumar SriwastvaChristyan Cruz UlloaMukesh Kumar ThakurChrysafis AndreouMukesh PrasadChu MaMukhtar AbdulkadirChuadhry Mujeeb AhmedMulugeta WayuChuan LiMumin CebeChuangchuang SunMumtaz Hussain SoomroChuang-Yuan ChiuMun’delanji C. VestergaardChuanliang LiMuneeb AhmadChuanqi TanMuneesh MaheshwariChuansi GaoMunish Kumar GuptaChuanxing BiMurad KhanChuanyin XiongMurat Kaya YapiciChuan-Zhi DongMurat SimsekChulhong KimMurillo Ferreira Dos SantosChulhun KangMurilo Araujo RomeroChun Hung ChuMurilo Bellezoni LoiolaChun LinMurtadha HssayeniChun Pang-joMurthy ChavaliChun QiMusab HameedChun ZhaoMustafa RajabaliChun-An ChengMustapha JouiadChunchi MaMustapha MaâtoukChunfeng MaMustaqeem MustaqeemChung Horng LungMutaz BarikaChung Ket TheinMuthaiah ShellaiahChung-Chi HuangMuthoni MasindeChung-Chiun LiuMuthu Ram Prabhu ElenchezhianChung-De ChenMuthuchamy NallalChung-Ho E. LauMuzaffar RaoChung-Hsun SunMyeong-Hun JeongChung-Liang ChangMyeounggon LeeChung-Min WuMykola SysynChung-Ping ChangMyounggon KangChunguang HuMyounggyu WonChunho YeomMyriam ZerradChun-Hsiang ChuangMyung Hwan ParkChun-Hsiang (Michael) ChuangMyungjun KimChunhua DongMyung-Ki KimChunhua SuMyung-Sup KimChunhua YangNabamita PalChunhui ZhaoNabeel MohammedChun-Hung LinNabil AshrafChun-Kai WangNabil BachaghaChunlei WuNabila BrihmatChunlei XiaNachappa GopalsamiChun-Ling LinNachiket TapasChunlong FeiNadarajah ManivannanChunming XiaNader NaghaviChun-Ping JenNader ShehataChunrong PengNader Taheri-QazviniChunsheng WuNadezhda YarushkinaChun-Wei TungNadia BeluChunxiao HuNadia DelavarpourChunxiu LiuNadia KanwalChunye XuNadica MiljkovićChun-Yeon LinNadir KapetanovićChun-Yin YehNadir ShahChun-Ying HuangNadjim HorriChunyu LinNadra GuizaniChun-Yuan LinNaeem AyoubChuong NgoNaeem IqbalChuong NguyenNafati AboserwalChuong V. NguyenNafees MansoorChuyi ChenNag AnindyaChyan-Long JanNagaraj YamanakkanavarCiavoi GabrielaNageswara LalamCicerone Laurentiu PopaNagham SaeedCiesielski ArkadiuszNaghmeh MahmoodianCigdem TuranNagy LajosCihan BayindirNahid MajdCiming ZhouNahina IslamCintia Micaela Chamorro PetronacciNaiara DemnitzCiprian ComsaNaim MitreCiprian DobreNajib KacemCiprian GiurcaneanuNajmeh KarimianCiprian LapusanNakju DohCiprian LupuNakul PandeCiprian NemesNalika UlapaneCiprian OrheiNallapaneni Manoj KumarCiprian PocolNallappan GunasekaranCiprian RezusNam HuynhCiro GioiaNam KimCise MidogluNam Tuan LeClaas FalldorfNamhoon KimClaire GoursaudNamhyuk HamClaire WaltonNam-Yong LeeClara Simon De BlasNan JiangClaude ChaudetNan LiClaudia CanaliNan QiClaudia ContiNan WuClaudia DaffaraNan XuClaudia GirjobNan YangClaudia Gonzalez ViejoNan YuClaudia KrullNan ZhaoClaudia LermaNan ZhouCláudia LopesNancy OstiguyClaudia MarziNane KratzkeClaudia PacurarNanjing HaoClaudia SciutoNannan LiClaudia ZoppettiNannan WangClaudine IrlesNaoki FukutaClaudine KraanNaoki ShinoaraClaudinei Rodrigues De AguiarNaoki WakamiyaClaudio Arancibia-IbarraNaomi CaselliClaudio BruschiniNaomi KakoschkeClaudio EstaticoNaoro TanakaClaudio M. De FariasNaoshi KanekoClaudio MarcheNaotaka NittaClaudio MirarchiNaoya AdachiClaudio OggeriNaoya KasaiClaudio PellegriniNaoya TsunodaClaudio SavaglioNaoyuki KataokaClaudio VasapolloNarayan Chandra Deb NathClaus MilkereitNarcisa Roxana MosteanuClaus PahlNaresh Kumar DarimireddyCleber A. AmorimNaresh Kumar RavichandranClélia Christina Mello-SilvaNarottam DasClemens RumpfNarrendar RaviChandranClement LorkNaser MostashiriClemente Ibarra-CastanedoNaser Ojaroudi ParchinCleonilson Protasio De SouzaNasim HajariCleopatra BardakiNasim NezamoddiniCliff LissendenNasro Min-allahClint HansenNasser Abu-ZeidCodruta VarodiNasser RezzougColin DrummondNatalia A. GouskovaCollodi GiovanniNatalia KamaninaColton GrasmickNatalia ResendeComan CristianNatalia ZhukovaConcepcion Luján-ÁlvarezNatalie BaddourCong PuNataliia LotoshynskaCong YangNatalija LepkovaCongcong MaNataliya V. BakhmetievaCongo Tak Shing ChingNataliya V. YurkevichCongzheng HanNatalya KondratyevaConrad RizalNatalya RyabovaConstantin ApetreiNatalya ShaburovaConstantin IlieNatan KopeikaConstantin NistorNataša NáprstkováConstantin OrasanNatasa NordConstantin PaleologuNataša SamardžićConstantin VertanNatasa TosanovicConstantin VolosencuNatasa ZivicConstantine KotropoulosNatassya SilvaConstantine MichailidesNathalie SauerConstantino Carlos Reyes-AldasoroNathan LauConstantinos K. ZacharisNathan LazarusConstantinos PatsakisNathan SalowitzConstantinos ValagiannopoulosNathaniel HuntCord M. BrundageNathir A. RawashdehCordos NicolaeNauman Khalid QureshiCorina DrapacaNavid MojtabaiCorina J. BîrleanuNavid ReyhanianCorina NüeschNawab Muhammad Faseeh QureshiCorinna HarmeningNawaf HamadnehCorinna SchmittNawal ElfishawyCorinne CorbauNawar KadiCornel BalintNayden NenkovCornel CobianuNazakat AliCornel IoanaNazanin NeshatvarCornelia MăireanNazarena BrunoCornelio Yáñez-MárquezNazek El-AtabCorneliu BurileanuNazia HameedCorneliu MunteanuNazmul BegCorrado BoragnoNebahat Şule ÜstünCorrado CostaNebojsa DoncovCorrado LaneraNebojša MaleševićCorrado RindoneNeda Haj-HosseiniCortino SukotjoNediljka Gaurina-MeđimurecCory DixonNédra MellouliCory M. SmithNeeraj BokdeCosimo GentileNefta-Eleftheria VotsiCosimo IeracitanoNeil BoonhamCosimo TronoNeil CollingsCoskun Joe DizmenNeil GlossopCoskun TekesNeil GriggCosmin AncutiNeil I. FoxCostantino BalestraNekane SainzCostas ChaikalisNel SamamaCostas LambrinoudakisNelcio Antonio Tonizza De CarvalhoCostas PanagiotakisNélia AlbertoCourtney Elaine StewartNelly LeligouCourtney RoperNelson BatistaCourtney ShellNemai Chandra KarmakarCreed JonesNenad GligoricCrhistian Raffaelo BaldoNengchao LyuCrispin MutshindaNengfang ChaoCristhian DuranNerija ZurauskieneCristian AndrieseiNerijus MorkeviciusCristian AxenieNestor Arana ArexolaleibaCristian BarzNestor Cabrera-MunozCristian BoldisorNéstor José Jarque BouCristian ChilipireaNestor Montalvan-BurbanoCristian Davalos-SaucedoNeven VrčekCristian De SantisNewcomb RobertCristian E. SimionNgoc-Huynh HoCristian FosalauNguyen Dinh DungCristian Marian MihaescuNguyen Ngoc-PhiCristian MateosNguyen Quang VinhCristian MoralNguyen Quoc Khanh LeCristian MușuroiNguyen Thanh-VinhCristian OleaNguyen Thi Phuoc VanCristian PasluostaNguyen TruongCristian RotariuNhat-Duc HoangCristian SimionNiall HigginsCristian TorresNiall O’MahonyCristian Tudor MateaNiamat HussainCristian Vacacela GomezNianyin ZengCristian VasarNibaldo RodriguezCristian VendittozziNiccolo BaldanziniCristian ViespeNiccolò DematteisCristian ZetNicholas A. GiudiceCristiana TudorNicholas CaporussoCristian-Cezar PostelnicuNicholas ChilesheCristian-Dragoş DumitruNicholas J. KinarCristiano CordeiroNicholas J. LongCristiano CostaNicholas JonssonCristiano De MarchisNicholas OeschCristiano Maria VerrelliNicholas RotellaCristiano PaggettiNicholas SamarasCristiano PalegoNicholas Vincent ApolloCristiano PremebidaNicholas VretosCristina Anna Maria Lo IaconoNicholas X. WilliamsCristina BaglivoNick Andrei IvǎnescuCristina Bianca PopNick DonaldsonCristina BignardiNick DraperCristina Caramelo GomesNick PitropakisCristina CaridadeNicola A MorleyCristina CastejonNicola BellomoCristina Cejudo BastanteNicola GiaquintoCristina ComanNicola LiberatoreCristina CortiNicola LopomoCristina De CastroNicola LudwigCristina Gonzalez-MartinNicola MarottaCristina Gutierrez-SanchezNicola MassariCristina Maria Ribeiro CaridadeNicola OrzalesiCristina Mihaela CampianNicola PellegriniCristina Mihaela NicolescuNicola SchulzCristina MuresanNicola TestoniCristina NuzziNicola ZemaCristina PronelloNicolae ConstantinescuCristina Rodriguez-SanchezNicolae GogaCristina Stolojescu-CrisanNicolae HerisanuCristina TortiaNicolae PopCristina-Laura Stolojescu-CrişanNicolae TudoroiuCristina-Sorina StangaciuNicolae–Doru StănescuCristine AgrestaNicolai SpicherCristóbal Barba-GonzálezNicolas Alexander TatlasCristóbal García ParienteNicolas AuclairCristoforo MarzoccaNicolas DalmedicoCsaba AntonyaNicolas Garcia-AracilCsaba Dobo-NagyNicolás Laguarda-MiróCsaba SzászNicolas Le SommerCuauhtémoc Sanchez RamírezNicolas P. AvdelidisCuma TyszkiewiczNicolas RoehriCun JiNicolas SpinelliCyril FischerNicolas StoulsCyrille BergerNicolas VerrierCyrille MigniotNicole Jaffrezic-RenaultCzeslaw SuchockiNicole W. XuD. Hudson SmithNicole ZahradkaD. Lansing TaylorNicoleta EnescuDabetwar ShwetaNicoleta GillichDac-Binh HaNicoleta IliesDachuan ShiNicoletta NicolaouDadmehr RahbariNicolo BeveriniDaehee KimNicolò MaccaferriDae-Hyun LeeNicolo MarconatoDae-Ki KangNicos MaglaverasDafna SussmanNicu Constantin TudoseDag Roar HjelmeNihan AydemirDagmar PavluNihanth W. CherukuruDagmara IwańskaNijolė BatarlienėDahai XiaNikhil BhallaDaiana Priscila Rodrigues-de-SouzaNikita AndriyanovDaifa WangNikita MinaevDaifeng PengNikita ShepelinDaisuke FujimotoNikitas NikitakosDaisuke MatsuuraNiko LukačDaiva MakutėnienėNikola AnđelićDajun WuNikola BanicDakun LaiNikola BaranDalbert Matos MascarenhasNikola GradojevicDalei HaoNikola IvkovićDalia De SantisNikola KranjčićDalia StreimikieneNikola LopacDalibor BartoněkNikola SakačDalibor CiprianNikola ZogovićDalibor ŠtysNikolai KornevDalius JuciusNikolai PerovDallan ByrneNikolai StoianovDalton Pessôa FilhoNikolai TsvetkovDalton SnyderNikolai UshakovDaluwathu Mulla Gamage PreethichandraNikolaj GoraninDalvan GrieblerNikolaos A. AndroutsosDamian DerlukiewiczNikolaos Athanasios AnagnostopoulosDamian GrzechcaNikolaos C. KokkinosDamian HudziakNikolaos DimitriouDamian KordosNikolaos DoulamisDamian KrawczykowskiNikolaos GiannakeasDamian VallesNikolaos K. LyrasDamiano CarusoNikolaos KantartzisDamiano PerriNikolaos KarathanasopoulosDamianos ChatzievangelouNikolaos MavridisDamien KinetNikolaos MitianoudisDamien LeducNikolaos NikolakisDa-Ming YangNikolaos NomikosDamir FilkoNikolaos PitropakisDamjan VlajNikolaos T. MilasDamon Shing-Min LiuNikolaos TapoglouDan ChiceaNikolaos TsalisDan CojocNikolaos V. KantartzisDan Cristian PopaNikolaos VitzilaiosDan DobrotaNikolaos-Alexandros TatlasDan FeldmanNikolas PontikosDan Garcia CarrilloNikolay BorodinovDan GrobnicNikolay KakanakovDan LiNikolay KazanskiyDan LuoNikolay MukhinDan Mircea SuciuNikolay ShilovDan MoldovanuNikolay SmaginDan NecsulescuNikolay Todorov AtanasovDan NeculoiuNikolina JankovicDan PescaruNikos A ChaniotakisDan SelisteanuNikos AntonopoulosDan SongNikos DimitriouDan WuNikos Dimitris FakotakisDan ZengNikos KonofaosDan ZhangNikos PetrellisDana BadauNikos SvigkasDana PetcuNikunj A. BhagatDana SimianNili KrauszDana StoianNils Tångefjord BasseDana Vasiljević-RadovićNils Von NeuhoffDan-Adrian MocanuNima JavanbakhtDana-Mihaela PetrosanuNima KarimianDandan MaNima MovahediD’Andrea LorenzoNimrod CarmonDane WesterdahlNimsiri AbhayasingheDanfeng HongNina DanilinaDang Khoa NguyenNina RizunDangdang ShaoNina SinatraDangirutis NavikasNinad MehendaleDani TostNing HuDaniel A. R. ChavesNing LiDaniel AlbuquerqueNing LiuDaniel AmoNing SunDaniel AntónNing WangDaniel ArvidssonNing XiDaniel BásconesNing ZhaoDaniel Basso FerreiraNingbo YuDaniel BaumgartenNing-de JinDaniel BenedikovicNingning HanDaniel CamposNingqi LuoDaniel Carnieto TozadoreNino F. LäubliDaniel CarruthNinoslav MajurecDaniel Chagas Do NascimentoNirav JoshiDaniel ChowNiroshinie FernandoDaniel ConduracheNirvana PopescuDaniel Costa RamosNirvedh MeshramDaniel CremonsNishant TripathiDaniel DiaconuNishanth Venugopal MenonDaniel DoyleNishikawa YuichiDaniel FiczereNita YodoDaniel Filipe AlbuquerqueNitish KatochDaniel FischerNitish ThakorDaniel Flores TapiaNitu SyedDaniel FodoreanNivaldo T. SchieflerDaniel G. CostaNivedita NiveditaDaniel GahremanNizam Uddin AhamedDaniel GaidaNizar JaberDaniel Gutiérrez ReinaNoa GarciaDaniel HeinNoé Pérez-HiguerasDaniel Hernández-SosaNoel BristowDaniel HimrNoelia Zagalaz AnulaDaniel HoffmannNojan AliahmadDaniel HomocianuNolwenn LapierreDaniel Ivan PinedaNoman NaseerDaniel J. SchadNoor Zaman JhanjhiDaniel J. SousaNoora IsoahoDaniel Jáuregui-VázquezNor Shahida Mohd ShahDaniel Jerez-MayorgaNóra BaranyaiDaniel (Jian) SunNoramaliza Mohd NoorDaniel KacikNorbert Chamier-GliszczyńskiDaniel KoningsNorbert LindleinDaniel KostrzewaNorbert PalkaDaniel LeightleyNorbert SchwesingerDaniel LisakNorbert WehnDaniel M. De LeonNordin RosdiadeeDaniel Martinez-MarquezNorihiro KamamichiDaniel MayerNoriko SaitoDaniel McGrathNorio MiyoshiDaniel MigliozziNorio TagawaDaniel Mihai TomaNoriyuki KawarazakiDaniel MolterNoriyuki KimuraDaniel Morinigo-SoteloNorma-Aurea Rangel-VázquezDaniel Octavian MelinteNorman AdamsDaniel PaternainNoshin FatimaDaniel PinardiNourdine AlianeDaniel PoenarNoushin NasiriDaniel RamosNozhan BayatDaniel Rodríguez PradoNozomi NishizawaDaniel RosnerNuggehalli M. RavindraDaniel SaramakNuno Alexandre Ribeiro CostaDaniel ŚlęzakNuno CruzDaniel SolaNuno GonçalvesDaniel StichNuno MendesDaniel SweeneyNuno Miguel Fonseca FerreiraDaniel TonelliNuno MoraisDaniel Ulises Campos-DelgadoNuno Pessanha SantosDaniel UrbánNuno RodriguesDaniela B. ClaroNunzio TorrisiDaniela C. VazNuria DuffoDaniela De BartoloNúria GavaraDaniela De VenutoNuria Novas CastellanoDaniela Díaz-AlonsoNuria Teigell BenéitezDaniela FogliNurul SarkarDaniela IsidoriNusret DemirDaniela JumancaNuvia M. SaucedoDaniela LupiOana GemanDaniela MaffiodoOana HosuDaniela MontiOana MitruțDaniela Pia Rosaria ChieffoObada Al ZoubiDaniela PöhnOctavia DobreDaniela TarnitaOctavian DospinescuDaniela TesařováOctavian DuliuDaniela-Elena PopescuOctavian FratuDaniele AncoraOctavian PostolacheDaniele BarettinOctavio Gutierrez FriasDaniele BorzelliOday Ibraheem AbdullahDaniele CafollaOded LiranDaniele CoccoOdongo Steven EyobuDaniele De MartiniOfer BarneaDaniele Di MitriOgnjen ArandjelovićDaniele EspositoOgnjen OrozovicDaniele FarnesiOktay YilmazogluDaniele FerdaniOkur SalihDaniele GiansantiOkuwobi Idowu PaulDaniele GiordanOkyay AltayDaniele GiustoOlaf GefellerDanièle HauserOlaf UeberschärDaniele LosannoOláh JuditDaniele MarinelliOlamide Timothy TawoseDaniele MartiniOlatunji Mumini OmisoreDaniele MasaroneOlatunji.Abdul ShobandeDaniele MerliOlav EgelandDaniele PannoneOle BangDaniele PiscitelliOleg A. MarkelovDaniele SofiaOleg A. StepanovDaniele SpoladoreOleg GolovninDaniele TosiOleg I LyaminDaniella Jorge De MouraOleg IlliashenkoDaniil N. BratashovOleg K. GolovninDanijela Ristic-DurrantOleg M. GradovDanil IvanovOleg MorozovDanil PimenovOleg MyakininDanilo AvolaOleg Olegovich EvsutinDanilo ComminielloOleg SergiyenkoDanilo GligoroskiOleg ShiryayevDanilo S. CatelliOleg StukachDanish FarooqOleg UdalovDaniyal HaiderOleg V. ButovDanling WangOleg V. IvanovDan-Marius DobreaOleg V. ZakharovDanny K. Y. WongOleksandr MakeyevDansheng WangOleksandr MalyuskinDante Mújica VargasOleksandr MolnarDanuta UmiastowskaOleksiy SydorukDanyang QinOlga BibikovaDanyil KovalovOlga BoytsovaDaodao HuOlga CherkasovaDaoliang LiOlga E. GlukhovaDaolin MaOlga FragouDaphne BazopoulouOlga KorostynskaDaping HeOlga KurasovaDarci OdloakOlga SergeevaDaria WotzkaOlga SokolovaDarija LemicOlga ZinovievaDarija Lušić VukićOlga-Joan KtenidouDario AntonelliOlimpiu StoicuţăDario AssanteOliver C. SaavedraDario BallariniOliver CuateDario CazzolaOliver FaustDario SabellaOliver MichlerDario SpillerOliver OziokoDario StelitanoOlivér TörőDario TagliaferriOlivia CurzioDario VieiraOlivier DalvernyDarius AndriukaitisOlivier DebeirDarius EidukynasOlivier J.N. BertrandDarius JegelevičiusOlivier MarcelotDarius PlonisOlivier SchalmDarius PopovasOlli JokinenDarius RudinskasOludayo OlugbaraDariush EbrahimiOlumide OluyisolaDariusz BismorOluwakayode OniretiDariusz BuchczikOluwatosin OluwadareDariusz FydrychOm Jee PandeyDariusz GozdowskiOmar Abdul WahabDariusz GrzybekOmar AguilarDariusz HorlaOmar DurrahDariusz KardaśOmar HamzaDariusz LitwinOmar JanehDariusz MakowskiOmar Longoria-GandaraDariusz MikaOmar NiboucheDariusz PazderskiÖmer Muhammet SoysalDariusz PuchalaOmid AkhavanDariusz SawickiOmid ZargarDariusz ŚwietlikOmneya AttallahDariusz TomaszewskiOmprakash KaiwartyaDariusz TomkiewiczOna LukoševičienėDariusz UlbrichOndrej FišerDariusz WięcekOndrej HanušovskýDariusz WójcikOndrej KrejcarDarja TopolšekOndrej StopkaDarko HuljenićOndřej StrakaDarko StipanicevOndřej VondroušDarko VasicOndřej ŽivotskýDarko VušakOnel Luis Alcaraz LópezDarryl BassettOnur AlevDarya NemovaOnur TokerDarya NovopashinaOonagh M GigginsDarya TishkevichOrazio AielloDas Avik KumarOrcan AlparDat NgoOrestis GeorgiouDatong LiuOrhan OzgunerDatong WuOriel FrigoDave GilmoreØrjan G. MartinsenDavid A. BenfieldOrlando Camargo RodríguezDavid A. ConstableOrsolya KissDavid Ahmedt-AristizabalOrtner MichaelDavid Baeza MoyanoOscar Adamuz-HinojosaDavid BarrettOscar Andres VivasDavid BerbrayerÓscar Barquero-PérezDavid BerryÓscar Belmonte FernándezDavid BerthebaudOscar BorlaDavid BlancoÓscar CarranzaDavid BlazevicOscar CasasDavid BoansiOscar CastilloDavid BolandÓscar De Francisco OrtizDavid BrčićÓscar DelCastillo-AndrésDavid BrusOscar DuqueDavid BuschOscar Ernesto-CasanovaDavid CabaleiroOscar Gutierrez-BlancoDavid Camarena-MartinezOscar Herrera-AlcántaraDavid C.C. LamOscar KjellDavid CharlesOscar NunezDavid ChenelerOscar ReinosoDavid Ching-Fang ShihOskar MarkoDavid Chunhu LiOskar SterleDavid ClunieOskar SzumskiDavid DobrockyOsvaldo GervasiDavid DominguezOswaldo BaffaDavid DubucOswaldo Hideo Ando JuniorDavid EagerOswaldo Lopez-SantosDavid Escobar-CastillejosOswaldo Morales MatamorosDavid EstradaOswaldo Ulises Juarez-SandovalDavid G. GobbiOtakar HorejšDavid Garzón RamosOtar AkanyetiDavid GauthierOtilia BobisDavid Gómez-AndrésOtman MrabetDavid Gómez-OrtizOtniel Portillo RodríguezDavid GosztolaOto HaffnerDavid Granados-LiebermanOtoniel Mario López GranadoDavid Guijo-RubioOttavia GiuffreDavid GuiraudOtto KauffmanDavid HästbackaOuk ChoiDavid HelyOuyang XiaoyingDavid Herrero PérezOveis Amir HoseinDavid HuangOvidio SalvettiDavid Jaime Cocovi SolbergOvidiu BordeanDavid Joon Huang ChuahOvidiu IvanovDavid L. CedeñoOvidiu StanDavid LiOxana VerkholyakDavid Lizcano CasasOyunchimeg ShagdarDavid Luviano-CruzOzgur ErkentDavid M. MackieÖzkan UğurluDavid Mancha-TrigueroOzminkowski RonDavid MasipPaavo NevalainenDavid MelendiPabasara WanniarachchigeDavid MercierPablo A. Garcia-SalaberriDavid Moreno MolinaPablo AngueiraDavid Moreno-HernándezPablo AquevequeDavid Murià-VilaPablo Cano MarchalDavid Naranjo-HernándezPablo CorralDavid O’SullivanPablo EscobedoDavid Pallarés AldeiturriagaPablo Fernandez MiajaDavid Peres Da RosaPablo Giménez-GómezDavid PerpetuiniPablo HorstrandDavid PletsPablo Luis López EspíDavid RempelPablo Manuel Millán-MillanDavid RhigerPablo MartinezDavid Rodríguez SanzPablo MunizDavid RogersPablo OteroDavid ScaradozziPablo Rodriguez GonzalvezDavid SedláčekPablo Rodríguez LuqueDavid StratosPablo VenegasDavid ThielPagger Ernst PeterDavid ThorpePai-Hsun ChenDavid ValientePal VargaDavid ValisPalahin VolodymyrDavid Van HammePalani ElumalaiDavid VetturiPalanivel KandasamyDavid Viejo-RomeroPamela ZontoneDavid ZuecoPanagiota-Kyriaki RevelouDavide AstolfiPanagiotis A. EliopoulosDavide BaccoPanagiotis A. KarkazisDavide BorroniPanagiotis AgrafiotisDavide BrunelliPanagiotis AlevrasDavide C. CritelloPanagiotis BarmpoutisDavide CalandraPanagiotis E. PintelasDavide ComitePanagiotis GeorgakisDavide ContePanagiotis GkonisDavide ForcelliniPanagiotis K. GkonisDavide MarsanoPanagiotis KarkazisDavide MericoPanagiotis Karmiris-ObratańskiDavide MoroniPanagiotis KourtesisDavide NottiPanagiotis KyratsisDavide PaganoPanagiotis PapageorgasDavide PirronePanagiotis PartsinevelosDavide PolesePanagiotis Radoglou-GrammatikisDavide RoccoPanagiotis SarigiannidisDavide Settembre BlundoPanagiotis StavropoulosDavide ValerianiPanagiotis TrakadasDavod PorehPanagiotis TsarabarisDavood KarimiPandian PitchipooDavor GracinPang-Wei TsaiDavor StankoPankaj BhowmikDavorin AmbrušPankaj SinghDavoud DastanPanos AchlioptasDavut IzciPanos FitsilisDavy P. GaillotPantelis FrangoudisDawei GePaola Di PietroDawei WangPaola FerrarioDawen ZengPaola SaccomandiDawid JanasPaola SansoniDawid PołapPaolo BellagenteDawid SzpakPaolo BertoncelloDawid SzurgaczPaolo CasariDawid WajnertPaolo CastaldiDawid WarchołPaolo CastaldoDaxin TianPaolo CastelliniDayakar Naik LavadiyaPaolo CavorettoDayi BianPaolo CrippaDayi ZhangPaolo Di LilloDaylamani-Zad DamonPaolo FacciDayong QiaoPaolo FinocchiaroDayu LiPaolo FormichiDa-Zhi SunPaolo FraccaroDe Xing LioePaolo GastaldoDebabrata MaityPaolo GiammatteoDebadrita PariaPaolo IntiniDebanjana GhoshPaolo Jose Cesare BiselliDebankur SanyalPaolo LinoDebao YuanPaolo MelilloDebbie RheaPaolo MercorelliDebiao LuPaolo MeriggiDebkalpa GoswamiPaolo Motto RosDébora Christina Muchaluat-SaadePaolo NepaDebora GriffinPaolo PascoloDeborah GalpertPaolo PasqualiDechao ChenPaolo PetriniDeclan O’LoughlinPaolo SernaniDedi SetiawanPaolo SommellaDeema AlshammaryPaolo SpagnoloDeepak BalramPaolo TrucilloDeepak Kumar KhajuriaPaolo ViscontiDeepak ThirunavukarasuPaolo ZaffinoDeepan BalakrishnanPaolo ZucconDeepika KoundalParameshachari B DDefu LinParashar DhakalDehai ZhangParastoo FarniaDeise Santana MaiaPari Delir HaghighiDeisy ChavesParia EsmatlooDejan DraganParikshit DuttaDejan DrajicParinaz AbiriDejan NikolicParsa OmidiDejan ŽagarPartarakis NikolaosDejan ZupanParth BhavsarDejin ZhangParthiban PazhamalaiDe-Jiu ChenParthiban RajanDejun GuoParviz SafadelDeliang XiangPascal AccoDelphine ChadefauxPascal AubryDemba DialloPascal DupuisDemetrio IeroPascal HirmerDemetrio LozanoPascal NicolayDemetrios TsesmelisPascal PeterDemi AiPascal SchirmerDe-Ming KongPasquale CianciDemóstenes Zegarra RodríguezPasquale D’AngeloDemosthenes D. VouyioukasPasquale De MeoDeng WangPasquale Di PalmaDeng XiaoPasquale GiungatoDengfeng ChaiPasquale TommasinoDengfeng KuangPatrice ForgetDengfeng PengPatrice MegretDenghui HePatrice SalzensteinDengpan YePatrícia A. PereiraDengyong WangPatricia ArroyoDengzhi LiuPatricia BroderickDeni Librado Torres-RomanPatricia DolezDenis BourgeoisPatricia Jovičević-KlugDenis ButusovPatricia KhashayarDenis ChikurtevPatricia L. Lakin-ThomasDenis Delisle RodriguezPatricia MartinkovaDenis GaroliPatrícia Raquel Vieira SousaDenis GracaninPatricia Sánchez-GonzálezDenis GuilhotPatricia Takako EndoDenis HamadPatricia VazquezDenis HorvathPatricio BulicDenis N. SidorovPatricio CatalanDenis SidorovPatricio ColmegnaDenis V. MakarovPatricio DominguesDenis VinnikPatricio OrdazDenise Paschoal SoaresPatrick AugustinDenise WilsonPatrick CalletDenise-Penelope KontoniPatrick D. KumavorDenise-Penelope N. KontoniPatrick DegenaarDeniz GençağaPatrick Erik BradleyDennis AlveringhPatrick FaircloughDennis EdlerPatrick KwongDennis FlanaganPatrick MounaixDeodato TapetePatrick PaitzDeog-Su ParkPatrick SeelingDeokwoo LeePatrick SteglichDepeng WangPatrick StegmannDequan ZhangPatrick Van TorreDer-Chyuan LouPatrick WongDerek M. HallPatrik FlegnerDerrick M. MottPatrik GrznárDerya BirantPatrik RichnákDes WatsonPatrizia GrifoniDesheng KongPatrizia LivreriDeshi LiPatrizia VizzaDesiderio Cano PorrasPatryk ZiolkowskiDesmond ChowPatxi CasasDetlef LazikPau Climent-PérezDeval MehtaPaul AnnusDevanjan BhattacharyaPaul BogdanDevarshi ShahPaul CelicourtDevrim AkcaPaul DavidssonDewey TaylorPaul E. RappDeyan LinPaul E. SummersDezhi HanPaul FaragoDezun ZhaoPaul GeladiDhammika JayalathPaul GoodrumDhananjay SinghPaul HavigDhanasekaran VikramanPaul HemerenDhanjai DhanjaiPaul KainenDheyaa Jasim KadhimPaul KrauseDhiren K. PradhanPaul Lik Hang LeeDhouha El HoussainiPaul N. WhiteheadDhugal LindsayPaul RobertsDi DongPaul RuffinDi JinPaul SchwarzbachDi PengPaul SestrașDi SunPaul SotiriadisDi WangPaul TavolatoDi WuPaul ThevenonDi YangPaul TucanDiaa AhmedienPaul WatsonDiana BerbecaruPaula BajdorDiana PopescuPaula Costa CastroDianbiao DongPaula LamoDiane GanPaula Ruiz-HincapieDiane M. StyersPaula SampaioDiane PetersPaula VianaDiange YangPauliina SalmiDianyin HuPaulina Latko-DurałekDidi ShePaulina LewińskaDieff L. VitaPaulina TrybekDiego Alexander Tibaduiza BurgosPaulius RuzgysDiego AntolínPaulius SkačkauskasDiego Barreto HaddadPaulo A. Raymundo-PereiraDiego BetancourtPaulo Alexandre S. Armada Da SilvaDiego Casado-MansillaPaulo CaldasDiego Dall’AlbaPaulo CoelhoDiego Di FrancescaPaulo De Mattos NetoDiego DomínguezPaulo DiasDiego Fernández IglesiasPaulo E. CruvinelDiego Gachet PaezPaulo Eduardo TeodoroDiego GalvanPaulo Gomes Da CostaDiego García-GilPaulo GoncalvesDiego Jaén-CarrilloPaulo Jorge PalmaDiego M. Jiménez-BravoPaulo Moisés CostaDiego OlivaPaulo MontezumaDiego PassosPaulo Montezuma-CarvalhoDiego Prieto HerráezPaulo ReisDiego Roberto Colombo DiasPaulo RibeiroDiego RomanoPaulo Ribeiro Lins JúniorDiego Sales-leridaPaulo Victor R. FerreiraDiego SušanjPavan PoudelDiego VergaraPavan TankasalaDieter FrensePavel AtanasoaeDieter SchrammPavel FialaDihan Md. Nuruddin HasanPavel HrnčiříkDijana Petrovska-DelacrétazPavel KarlovskyDikaia E. SaragaPavel KoštialDilek Koc-SanPavel KovářDilpreet BuxiPavel KrasnovDima CheskisPavel LafataDimah DeraPavel LoskotDimas Irion AlvesPavel MelnikovDimitar Nikolov NikolovPavel NeužilDimitra K. ToubanakiPavel OtřísalDimitra TsounidiPavel RaškaDimitri BelliPavel RipkaDimitrios A. ExarchosPavel SegecDimitrios A. VrachatisPavel StefanovičDimitrios BargiotasPavel TofelDimitrios BechtsisPavel TuralchukDimitrios DechouniotisPavel V. MatreninDimitrios DoukasPavel ZemcikDimitrios E. KoulouriotisPavle SkočirDimitrios FotiadisPavlos PapadopoulosDimitrios KaterisPavol HelebrandtDimitrios KatsantonisPavol KajánekDimitrios LoukatosPavol KurdelDimitrios MenychtasPavol LengvarskýDimitrios PanagiotidisPavol LipovskýDimitrios PliatsiosPawan PathakDimitrios PolitikosPawel Andrzej LaskiDimitrios PsychasPaweł BorkowskiDimitrios PyromalisPaweł BurdziakowskiDimitrios RaptisPaweł DymoraDimitrios S. ParaforosPaweł GaćDimitrios SkarlatosPaweł Grzegorz GilewskiDimitrios StratakisPaweł HerbinDimitrios TsaopoulosPaweł J. SwornowskiDimitrios TselentisPaweł KaniewskiDimitrios VlachopoulosPawel KasprowskiDimitrios VogiatzisPawel KleczynskiDimitrios-Lavrentios ProusalisPaweł KomadaDimitris DechouniotisPaweł KostyłaDimitris GiakoumisPaweł KrzaczekDimitris J. PanagopoulosPaweł KudelaDimitris KalogerasPaweł LigęzaDimitris KanellopoulosPawel LinekDimitris KarampatzakisPawel MacekDimitris MourtzisPawel MadejczykDimitris SpathisPawel MarcDimitris VrakasPaweł MazurekDimka I. IvanovaPaweł ModrzyńskiDimos A. TriantisPaweł OlejnikDimos TriantisPawel PawlikDinara SobolaPaweł PawłowskiDinesh Veeran PonnuveluPaweł PiotrowskiDing WangPaweł PiskurDingde XuPaweł PopielskiDingfu ZhouPaweł RomanowiczDingkang WangPaweł RosaDingtian YangPawel RzucidloDinh Hoa NguyenPaweł S. WróbelDinh-Thuan DoPaweł SikoraDinko BegušićPawel SkokowskiDinko OleticPaweł SkruchDinko OsmankovicPaweł SutowskiDino DobrinićPaweł SzabelskiDiogo GomesPawel TysiacDiogo MonteiroPaweł WierzbaDiogo TecelãoPaweł ZalewskiDionisio RodriguezPaweł ZiolkowskiDipankar BeheraPaweł ZmarzłyDipankar MaityPayam Shams GhahfarokhiDipankar MitraPay-Liam LinDirk Hueske-KrausPaz Morer-CamoDirk SöffkerPedro A. MoraisDirkJan VeegerPedro BeirãoDivanizia Do Nascimento SouzaPedro Bertemes-FilhoDivij BhatiaPedro BrancoDiyan Minkov DimitrovPedro C. Junior OliveiraDjaffar Ould AbdeslamPedro CarmonaDjamal GacemiPedro CasauDjedjiga BelfadelPedro Castillejo ParrillaDmitri E. KvasovPedro CruzDmitrii TumakovPedro D. Frazão CorreiaDmitriy A. BukreevPedro Dinis Loureiro SalgueiroDmitriy Aleksandrovich MartyushevPedro E. Lopez-De-TeruelDmitriy KaramovPedro EncarnaçãoDmitriy KritskiyPedro FaiaDmitriy PotapenkoPedro Fernández ÁlvarezDmitriy V. SotnikovPedro FonsecaDmitriy Yu. OzherelkovPedro ForteDmitry A. BykovPedro Henrique Lopes SilvaDmitry BankovPedro J. Sanchez-CuevasDmitry BudkerPedro Javier Gamez MonteroDmitry GavrilovPedro Javier HerreraDmitry IlvovskyPedro M. B. TorresDmitry KarpovPedro Manuel Calas Lopes PachecoDmitry KireevPedro MateusDmitry KorzunPedro MêdaDmitry MurashovPedro MeloDmitry NamiotPedro Mendonça Dos SantosDmitry O. DolmatovPedro Miguel Alves Ribeiro CorreiaDmitry PavlyukPedro Miguel Ortega-CabezasDmitry PetrovPedro Moreno-SanchezDmitry RoshchupkinPedro O. RosselDmitry SkvortsovPedro Oliveira Conceição JuniorDmitry TelyshevPedro Orgeira-CrespoDmitry VlasovPedro P. B. BeakliniDmytro VasylyevPedro Pablo Garrido AbenzaDo Hyeun KimPedro Palos-SánchezDobromir PressyanovPedro PinhoDobrosława KasprowiczPedro PiquerasDochshanov M. AldenPedro RibeiroDockhorn AlexanderPedro Valdivia-MoralDoga BilicanPedro Valero-LaraDohoon KimPeichang ZhangDoina PislaPeichao GaoDomagoj TolicPei-Chen KuanDomen VerberPei-Chi ShaoDomenico BianchiPeifeng MaDomenico CannatàPeiheng ZhouDomenico CiuonzoPei-Huang DiaoDomenico Di MauroPeiliang XuDomenico GuidaPeilong HongDomenico M. DoronzoPeipei LiuDomenico PascarellaPeipei WangDomenico SperanzaPeiyuan ZhouDomenico SurianoPeiyun ZhangDomenico UrsinoPekka PirinenDomin KohPelagia GawronekDomingo MarreroPeng ChenDomingos De Sousa MenesesPeng FangDominic J. WalesPeng FuDominic KristalyPeng HuDominic O’ConnorPeng LiDominic WalesPeng LiuDominik ŁuczakPeng MiaoDominik ObristPeng QiDominik RybarczykPeng ShaoDominik SaulPeng SongDominik SiemonPeng TaoDominik SkokandićPeng Un MakDominique BéginPeng WangDominique FourerPeng WuDominique PagnouxPeng YinDominique RissoloPeng YuDomonkos VargaPeng ZengDon Chathurika AmarathungaPengcheng JiaoDonal G. SinexPengcheng LiuDonald FernandesPengfei LiuDonald HondongwaPengfei TianDonald MuellerPengfei WangDonald R. ReisingPengfei ZhangDonatella DarsenaPenghui WangDonatella GranataPengmin PanDonato ConteducaPengpeng HuDonato Hernández FusilierPengwen XiongDonato Luna MorenoPengxiang ZhaoDonato RomanoPengyu LvDong ChenPengyuan ZhouDong GuanPensieri SaraDong Hyun JeongPentti NieminenDong KimPeppino FazioDong Kun NohPer Håkan LundowDong LiPer NordbladDong LiangPere Vila TalledaDong MengPericle PerazzoDong Min KimPerkowski MarekDong WangPero VidanDong Won KimPetar RadanlievDong YangPeter A LieberzeitDongbao JiaPeter Adam HoeherDongdong MuPeter BeekDon-Gey LiuPeter BiermannDongfeng HePéter BöröczDongheon LeePeter BridaDonghoon KimPeter CentenDongHu NiePeter ChongDonghuan QinPeter CornillonDonghui XiePeter DragicDonghyeok AnPéter FöldesyDonghyun KimPeter GegeDong-il ChoPeter HauserDongil HanPeter HofmannDongjian HePeter HubinskyDong-Joo KimPeter J. GlynnDongkai YangPeter KaššayDongkyu LeePeter KaulDonglai ZhangPeter KazanzidesDongliang XiaoPéter KissDongmei HaoPeter KönigDongming XiaoPeter KostalDongming ZhangPéter KovácsDongping XiaoPeter L. StavinohaDong-Seong KimPeter LearyDongsheng LiPeter MarotiDongsheng ZhangPeter Meerwald-StadlerDong-Shik KimPeter MikuleckýDongshu WangPeter OffermansDong-Soo KwonPeter OttDong-Sop RheePeter PerešíniDongwei RenPeter PoctaDongwon JungPeter RolfeDongxing CaoPeter RösslerDongyan WeiPeter RyanDongyi ChenPeter SarcevicDongyi WangPeter Schulze LammersDong-You ChoiPeter SevenichDongyu ZhangPeter SinčákDongyuan ShiPeter StützDoo Heon SongPeter SuDoo-Hyun ChoiPéter SzolgayDoo-Seung UmPeter TeunissenDora Luz OjedaPeter TrúchlyDorian CojocaruPeter VeltinkDorian J. BurnettePeter WankeDorijan RadočajPeter WilliamsDorin MoldovanPeter WoutersDorota DziurkaPeter ZollikerDorota Iwaszkiewicz-GrzesPeteris Apse-apsitisDorota LewczukPetr BenešDorota Oszutowska-MazurekPetr BoriskovDorota Siwicka-GierobaPetr BouchnerDorota ZawieskaPetr ČervaDörte SollePetr DolezelDoru UrsutiuPetr EliášDoru-Laurean BaldeanPetr HájekDou ZhangPetr HlubinaDoug KurrantPetr HubacekDouglas A. BrooksPetr HurtíkDouglas BarbinPetr KoudelkaDouglas G. GoodinPetr LehnerDouglas O’ShaughnessyPetr MarcianDouglas RutledgePetr MarconDragan DinulovicPetr MlýnekDragan IndjinPetr SedlákDragan PamucarPetr SkládalDragan PerakovicPetr ŠtěpánDragan PerakovičPetr VachataDragana BajicPetr ZhilyaevDragana PerićPetra ĐurovićDrago BračunPetra JansenDrago StrlePetre Daniel MatasaruDragoljub GajicPetre LameskiDragos ArotărițeiPetri RönnholmDragoş Ioan SǎcǎleanuPetrică C. PopDragos IsvoranuPetros BithasDragutin LisjakPetros KoutsovitisDrew BudnerPetros PistofidisDrongelen Stefan VanPetros S. BithasDror MalkaPetru GhenucheDu Hwan KimPetrucio Ricardo Tavares De MedeirosDua OezsoyluPham Dinh BaDuarte Manuel Azevedo FernandesPhani Sankar NidadavoluDuc-Duy HoPhilip BranchDuc-Thinh PhamPhilip ChadwickDuc-Tien NguyenPhilip FuchsDugan UmPhilip J. BasfordDulcinea AvourisPhilip LerouxDumitrescu CatalinPhilip MooreDumitru CristianPhilipp Del HougneDumitru PopescuPhilipp GuldeDumitru ToaderPhilipp M. SchollDumitru VieruPhilipp M. SiebergDung-An WangPhilipp TerhörstDunja SrpakPhilipp WojaczekDunwen WeiPhilippe ChristolDunzhu XiaPhilippe GueguenDuo LiPhilippe RavierDuo ShengPhillip BedggoodDuo Wai Chi WongPhillip MillarDuojin WangPhong B. DaoDuong LePhuc NguyenDusan GleichPhuong T. NguyenDušan KopeckýPi-Chung WangDusan MagaPier Carlo BerriDušan ŠimšíkPier Giuseppe AnselmaDushhyanth RajaramPier Matteo BaroneDustin ShippPier Nicola SergiDuy Le ThaiPierangela SamaratiEberhard LehmannPiercarlo DondiEbrahim GhaderpourPierfrancesco FogliaEbrahim NajafzadehPierluigi BelliEce ÖzkanPierluigi RitrovatoEce UykurPierpaolo LoretiEda MarchettiPierpaolo PalumboEddy KruegerPierre BoulangerEde BodokiPierre ExertierEdelberto Franco SilvaPierre GrangeatEden Morales NarváezPierre LacroixEder Mateus Nunes GonçalvesPierre MarcotteEdgar ArribasPierre MarechalEdgar GutiérrezPierre MerdrignacEdinéia Aparecida Dos Santos GalvaninPierre-Edouard PortierEdison Pignaton De FreitasPierre-jean BouvetEdita Rosana WidasariPierre-Paul VidalEdite FigueirasPierre-Yves Collart-DutilleulEdmanuel CruzPierre-Yves De MüllenheimEdmundas Kazimieras ZavadskasPierre-Yves Le BasEdna Dias CanedoPieter BonteEdna PossanPieter MonsieursEdoardo FiorilloPieter VandervoortEdoardo RellaPietro AricòEdoardo RubinoPietro BoccadoroEdouard IvanjkoPietro BurrascanoEdson Eyji SanoPietro CacialliEdson Jansen MirandaPietro CataniaÉdson Luis BolfePietro MonforteEdson MitishitaPietro NannipieriEduard FiguerasPietro PiazzollaEduard LlobetPietro PicernoEduard MuslimovPi-Guey SuEduard S. SopinPilar Cea MinguezaEduard StoicaPilar García-DíazEduard ZadobrischiPilar JiménezEduarda MendesPilar Manzanares-LopezEduardo ArtalPilar MarinEduardo BarreraPilar Martín-MartínEduardo Cabal-YepezPilar Pérez-RosEduardo Casilari PérezPin LvEduardo CuestaPinaki PalEduardo G. SouzaPing LuEduardo JuarezPing LuoEduardo M. G. RodriguesPing WangEduardo PalermoPing XieEduardo Parente RibeiroPingan PengEduardo QuevedoPing-Hui LeeEduardo ScheerenPingjie HuangEduardo TeixeiraPingwei LiuEduardo TorresPingyi FanEdward BormashenkoPiotr ArtiemjewEdward HoppePiotr BońkowskiEdward Jacek GorzelańczykPiotr BorkowskiEdward JonesPiotr BorowikEdward Kah Wei TanPiotr CheluszkaEdward KozłowskiPiotr ChrostowskiEdward Stanisław OczeretkoPiotr CoftaEdwin OrdoukhanianPiotr CzechEdyta KucharskaPiotr DerugoEdyta Paczos-GrzedaPiotr DrozdowskiEe Jin TeoPiotr DulianEfraim WinocurPiotr DziurzanskiEfrain Mejia-BeltranPiotr FabianEfraín Santiago-RodríguezPiotr GaudenEfstathios KonstantinidisPiotr GawdaEfstratios KarantanellisPiotr GazdaEfthimios KarymbalisPiotr GębaraEfthymios N. LallasPiotr GierlakEftim ZdravevskiPiotr GołębiowskiEftychia PinakoulakiPiotr HerbutEggo U. Thoden van VelzenPiotr J. DurkaEgidio D’AmatoPiotr Jankowski-MihułowiczEgidio LofranoPiotr JaworskiEgor ErshovPiotr KacejkoEhab Mahmoud MohamedPiotr KiedrowskiEhsan KhoramshahiPiotr KiełczyńskiEhsan Kiani OshtorjaniPiotr KolasińskiEhsan NazemiPiotr KorbelEiichi WatanabePiotr KrauzeEiji YoshiharaPiotr KurganEilif HjelsethPiotr LesiakEimuntas ParšeliūnasPiotr LulińskiEirini Eleni TsiropoulouPiotr MajEkaterina BorisovaPiotr MarkowskiEkaterina KutafinaPiotr MichalakEkaterina R. GasilovaPiotr MillerEkaterina V. KunitsynaPiotr MiluskiEkim YurtseverPiotr MitkowskiEkin OzerPiotr MorasiewiczEklas HossainPiotr NapieralskiEl Mezyani TouriaPiotr NawrockiEla TarakciPiotr PałkaElad DentePiotr Piotr LipińskiElbaz AbouelmagdPiotr PorwikElder Alpes De VasconcelosPiotr PoznanskiEleazar Aguirre AnayaPiotr RegulskiEleftherios SdoukopoulosPiotr S. SzczepaniakElena A. GrigorievaPiotr SauerElena A. ZverevaPiotr SawickiElena AmaricaiPiotr SkrzypczyńskiElena BasanPiotr SobotkaElena Carlotta OlivettiPiotr StawickiElena Constanta AmăricăiPiotr TomczukElena DamiáPiotr WisniowskiElena De CosPiotr WodarskiElena DilonardoPiotr WrobelElena DoynikovaPiotr ZielinskiElena GubarPires IvanElena HelereaPiyapong JanmaimoolElena Hernández-PereiraPiyush YadavElena KozlovaPlácido Rogerio PinheiroElena Lopez-AguileraPo-Chang HsuElena LucchiPo-Cheng ChangElena M. Hernández PereiraPo-Chin YangElena MoraPohl LászlóElena MorottiPol HenarejosElena NavarroPolina P. KuzhirElena NechitaPolona SprajcElena Niculina DragoiPolyzois SoumplisElena OlivettiPoopalasingam SivakumarElena PivarciovaPo-Ruey LeiElena PolisadovaPosen LeeElena PotapovaPoushali DasElena PrudnikovaPo-Wen ChiElena Roibás-MillánPrabal BaruaElena SavelovaPrabhuraj D. VenkatramanElena Simona LohanPradeeban KathiraveluElena SizikovaPradeep R. VaradwajElena StefanaPradeep SharmaElena TomsikPradip DasElena TortaPrajowal ManandharElena V. EmelyanovaPrakash DuraisamyElena VillaPrakash GangadaranElena ZaitsevaPramod K. B. RangaiahEleni G. SymeonakiPranav A. BhounsuleEleni GianniPraneel ChandEleni KokinouPrashant PandeyEleni KoustriavaPrashanth Kond GokuldossEleni StrouliaPrashanth Konda GokuldossEleni VerykoukiPrashnna GyawaliEleonora AlfinitoPrasit CholamjiakEleonora MacchiaPrasun Kumar NayakEli FlaxerPrateek M. ShresthaEli ShlizermanPrateek NegiElia GattoPrateek VermaElia GruesoPratik SamantEliana Costa E SilvaPratikkumar DesaiElias DritsasPratima LabrooElías Escobar-GómezPratip RanaElias FakirisPratiti BhadraElias GlytsisPratool BhartiElias HamannPraveen DamacharlaEliete CaldeiraPraveen Kumar DontaEligiusz PawłowskiPrem Chandra PandeyElio RomanoPrem Kumar ChaurasiyaElisa BassoliPremysl HudecElisa Cabana Garceran Del VallPrince WaqasElisa CastagnolaPriscila M. KosakaElisa KallioniemiPritam BoseElisa Mariarosaria FarellaPritish ChakravartyElisa Mejía-MejíaPriyabrata PradhanElisa PaneroPriyadarsi NandaElisa ScalcoPriyanka KarnatiElisabetta CanettaPriyanka SinghElisabetta MartiniProdromos-Vasileios MekikisElisabetta SieniProloy DebElisavet (Ellie) K. StathopoulouProttoy HasanElise ShepleyPrzemyslaw Falkowski-GilskiEliska SedlackovaPrzemysław GłombElizabeth Rendon-MoralesPrzemysław GłowackiEllen CesewskiPrzemyslaw HermanEllen WilliamsPrzemysław KlapaElli PetsaPrzemysław KurasElman ShahverdievPrzemysław LisińskiElmer Alexis Gamboa PeñalozaPrzemysław MazurekEloisa Dezen KempterPrzemysław MiąsikEloisa MacedoPrzemysław PodulkaEloy López MenesesPrzemysław PrzygodzińskiElsa GarmirePrzemyslaw PtakElsa M. MacíasPrzemysław WachulakÉlvio Rúbio GouveiaPui-Chuen HuiElvira Fernandez-AhumadaPukar MaharjanElyse PassmorePuneet MishraElżbieta JasińskaPuran PandeyElżbieta Krawczyk-DembickaPuren OuyangElżbieta KuberaPyke TinElżbieta Łodyga-ChruścińskaPyo-Woong SonElżbieta MacioszekQaisar AbbasElżbieta RoszkowskaQaisar AbbassEmad SamirQaiser RiazEmad Samuel Malki EbeidQammer H. AbbasiEmanuel AldeaQasem Abu Al-HaijaEmanuel GuarigliaQasim AhmedEmanuel PeresQi LiuEmanuel PuschitaQi WangEmanuela NataleQi ZhangEmanuele BarcaQian DuEmanuele CardilloQian FengEmanuele CerrutoQian HuangEmanuele FrontoniQian LiEmanuele Gabriel MargheritaQian SunEmanuele PiuzziQian WangEmanuele SantiQian YangEmanuele TortiQiancheng ZhaoEmerson GalvaniQiang DuanEmi YudaQiang LiuEmil CazacuQiang LvEmil DumicQiang ShenEmil DumićQiang TangEmil Engelund ThybringQi’ang WangEmil J. KhatibQiang WuEmil N. SobolQianguo XingEmil PricopQiangxian HuangEmilia Den BoerQiangyu ZengEmilia MihaylovaQiangyuan ZhuEmilia OgodescuQianhong ChenEmiliano CèQianru JinEmiliano DescroviQiao LiEmiliano TramontanaQibo DengEmiliano ZampettiQibo FengEmilio AndreozziQican ZhangEmilio MartinesQichang MeiEmilio MatriccianiQichong ZhangEmilio SanjurjoQiguo RongEmilio SardiniQihang QiuEmily HaoQiji ZeEmily Sara MatijevichQijin ChenEmir BencaQijin HuangEmma OzanichQijin ZhangEmmanouel T. MichailidisQijing LinEmmanouil BarmpounakisQilin HuaEmmanouil G. SpanakisQimei CuiEmmanouil GagaoudakisQing ChangEmmanouil PsomiadisQing (Ken) YangEmmanuel AguQing LuEmmanuel ConchonQing XueEmmanuel Karlo NyarkoQing YangEmmanuel Le ClézioQing ZhangEmmanuel MaravelakisQingdong OuEmmanuel MarinQingfeng LiuEmmanuel Ojo AdemolaQingguo DuEmmanuel RamassoQingguo LiEmmanuel TopoglidisQinghe ZhengEmmelyn GrahamQinghua HuangEmre SalmanQingjun ZhangEn WangQinglan DingEnea ParimbelliQingmin YuEnes ErdinQingqi PeiEngin CiftyürekQingshan SheEngin ZeydanQingsong WangEnji SunQingtian DengEnnio BilanciniQingwen HanEnnio GambiQingxi ZengEnrica PetrucciQingxu DengEnrico BabilioQingyang WeiEnrico CaloreQingyong LiEnrico ConcaQingyu CuiEnrico CortiQingyun DuEnrico DentiQingzhou MaoEnrico FerrentinoQinjun WangEnrico G. CaianiQinpei ZhaoEnrico Maria StaderiniQiong RanEnrico MeliQiong WangEnrico PerriQiongfeng ShiEnrico PetritoliQipei MeiEnrico PisoniQitao TanEnrico RavagliQiu JunEnrico StaderiniQiuguo ZhuEnrico VeronaQiuliyang YuEnrico VezzettiQiuming ZhuEnrique A. Navarro-CambaQiuping JiangEnrique BarónQiushi FuEnrique BronchaloQiwen QiuEnrique Chirivella-PerezQiyao HuangEnrique CoronadoQizhen SunEnrique GuerreroQizhi XuEnrique Hernández-BalagueraQonita Kurnia AnjaniEnrique Hernández-OralloQuan ChaiEnrique HortalQuan-De WangEnrique J. Herrera-LópezQuang HaEnrique Melgoza-VázquezQuang Hung DoEnrique Nava-BaroQuang Hung TranEnrique PrefasiQuanqin ShaoEnza PanzardiQuanying WuEnzo RizzoQuetzalcoatl Cruz Hernández-EscobedoEoin O’ConnellQun SunEpimitheas GeorgitzikisQunsheng CaoErasmo CadenasQuoc-Anh TranErdenebayar UrtnasanQuoc-Hung PhanErdenebileg BatbaatarQuoc-Thong NguyenErez RibakQutaiba AlasadErfan Shojaei BarjueiQuyuan LuoErhan EkmekciogluR. Chad WebbEric BerksonR. DhayaEric C. HonertR. Jacob BakerEric CampoR. Ranjith PremasiriEric ChappelR. Simon SherrattEric De Souza GilRabab BenotsmaneEric FujiwaraRabie A. RamadanEric HetlandRabih YounesEric J. R. ParteliRaby HamadiEric J. ZhangRachel VitaliEric LuiRachel ZuanonEric (Tae H.) LeeRachid BenouiniEric WadeRachid NedjaiEric WebsterRachid SaadaneErica NocerinoRachna ValvaniErica VoltaRadek ŠmídErick Andres Perez AldayRadek StrnadErick Reyes VeraRadhya SahalEricka Janet Rechy-RamírezRadim BurgetErik EtienRadka NachevaErik HeijneRadoslav TomovićErik KajatiRadoslav VargicErik KučeraRadosław BelkaErik LinsteadRadosław CioćErik VavrinskýRadosław KlimekErika BrattichRadosław MiśkiewiczErika EskinaRadosław PodsiadłyErika ZemkováRadosław PomećkoErlend LarsenRadostina A. AngelovaErmanno PietrosemoliRadovan HolubekErnest CzermańskiRadu CiorapErnest TyburskiRadu ColibanErnesto De La Cruz SánchezRadu ComesErnesto LimitiRadu Gabriel BozomituErnesto Moya-AlborRadu HobincuErnesto SifuentesRadu RadescuErnesto Zambrano-SerranoRadu TarcaErnst NiederleithingerRadu Tudor IonescuErol GelenbeRadu VacareanuEros BorsatoRaed Abd-AlhameedEros MontinRa’ed MalallahErricos-Chaim VentourasRafael Abrantes PenchelErtugrul TacirogluRafael Aguilar-GonzalezErvin K. LenziRafael AlvarezErvin KamenarRafael Asorey-CachedaErving XimendesRafael Berlanga-LlavoriErwin KristenRafael C. CardosoErwin PeinerRafael CasadoErwin WojtczakRafael Cisneros-MagañaEryk WolarzRafael Furlan De OliveiraEsben Søvsø Szocska HansenRafael Gonzalez-LandaetaEshagh Farzaneh JoubanehRafael KunstEsma DervisevicRafael Martínez PeláezEsmeidel LealRafael Messias MartinsEsra AkbasRafael MolinaEssam RashedRafael Morales HerreraEstate (Tato) SokhadzeRafael Pastor VargasEsteban IngaRafael Perazzo Barbosa MotaEsteban MunicioRafael Perez Del RealEsteban Obrero-GaitánRafael Pires De LimaEsteban Peña PitarchRafael RadkowskiEsteban PinoRafael Ravina-RipollEsteban Tlelo-CuautleRafael Raya LópezEsteban VenialgoRafael SantosEstefanía Núñez CarmonaRafael Timóteo De Sousa JúniorEster D’AccardiRafael Vargas-BernalEsther Medrano-SánchezRafal DorozEsther SalamíRafał DreżewskiEstrela Ferreira CruzRafał GołębskiEstrella RausellRafał GrądzkiEthan BayneRafał GrzejdaEthan FetayaRafał J. DoniecEtienne LordRafał KaźmierczakEtienne LossonRafał KowalikEuclides Lourenço ChumaRafal PrzesmyckiEugen LupuRafał SieńkoEugen Mircea AnitasRafał SzukiewiczEugen PfannRafał WojszczykEugene EremchenkoRaffaele CarliEugene PostnikovRaffaele GravinaEugenia Fagadar-CosmaRaffaele MartoranaEugenio AgugliaRaffaele RiccoEugenio CavalloRaffaele SalernoEugenio IvorraRaffaele ScuratiEugenio Marino MerloRaffaele VelottaEugénio RodriguesRaffaele ZinnoEugenio Romanchik KriuchkovaRafik HamzaEugeniusz KodaRagab El SehiemyEugeniusz RosolowskiRahim RahmaniEugin HyunRahman AliEui Chul LeeRahul DattaEulalia BalestrieriRahul GomesEulicio De Oliveira Lobo-JuniorRahul MouryaEun Joo RheeRahul Reddy NadikattuEun Seo ChoiRaihan Ul IslamEunho LeeRaimon CasanovaEunil SeoRaimondas PomarnackiEunjung ChoiRaimondas ŠadzevičiusEunsongyi LeeRaimund KirnerEuripidis GlavasRaimundo Jiménez BallestaEusebio De-la-FuenteRaine ViitalaEusebio Eduardo HernandezRainer FalkEusebiu Ilarian IoneteRainer TutschEva ÁlvarezRaivo SternEva BagyinszkyRaj K. GCEva CernadasRaj KumarEva DokladalovaRaja BalaguruEva H. DulfRaja NagisettyEva Krieghoff-HenningRajagopalan ThiruvengadathanEva M. Santos LópezRajalekshmi C. PillaiEva MaiaRajan KadelEva María CirugedaRajani SinghEva Mihalyko-OrbanRajendra DhakalEva Villegas PorteroRajendra N. BhadaneEva VolnáRajesh KumarEvaggelos KaselourisRajesh Kumar TripathyEva-Maria DölkerRajeshwara Chary SriramadasuEvan O’KeeffeRaji GhawiEvando AraújoRajkumar SainiEvangelia KaragianniRaju AedlaEvangelia KouidiRaju ChettyEvangelia VouvoudiRalf BergmannEvangelina LaraRalf FabianEvangelos HristoforouRalf LübbenEvangelos K. MarkakisRalf MouraEvangelos KeramarisRalf StetterEvangelos MaltezosRalf ZieboldEvangelos MarkakisRalu DivanEvangelos RoussosRam K. MazumderEvdokimos KonstantinidisRam Kumar SelvarajuEvelio Ramirez MiquetRam M. NarayananEvelyne KnappRam NarayananEverardo Inzunza-GonzálezRama Sashank MadhurapantulaEverardo Vargas-RodriguezRamachandran KasuEvgenia AdamopoulouRamanaskanda BraveenthEvgenia NovikovaRamasamy MouliEvgenii I. PonomarevRameez AsifEvgenij Olegovich Ol’HovikRamesh NepalEvgeny A. SemenishchevRamez AbdallahEvgeny BelyaevRami Ahmad El-NabulsiEvgeny KhorovRami CohenEvgeny MirkesRami Mustafa A MohammadEwa GorodkiewiczRami ZewailEwa KorzeniewskaRamin Banan SadeghianEwa PawluszewiczRamin GhiasiEwa PopkoRamin RahmaniEwa RopelewskaRamin YahyapourEwa SobolewskaRamiro S. BarbosaEwa WaszkiewiczRamiro VelázquezEwan ThomasRamon AgüeroEwaryst TkaczRamón Alberto Mollineda CárdenasEzequiel CoscuetaRamón AlcarriaEzio PreatoniRamon Antonio Rodriges ZalipynisEzzaldeen EdwanRamon Aparicio-PardoF. Fernando Jurado-LassoRamón BarberFabbrocino GiovanniRamon CasanellaFabian Andres Lara MolinaRamón De La RosaFabian FriederichRamon Dos Reis FontesFabian HöflingerRamon EritjaFabian LorigRamon FontesFabian MederRamon García HernándezFabian ReuterRamón González-CamarenaFabián RiquelmeRamón MorenoFabian SeguelRamon Parra-MichelFabiano FerrariRamon VillarinoFabiao YuRamona CimpoesuFabien BogardRamona GalatusFabien BuisseretRamūnas ČesnavičiusFabien MieyevilleRan ChenFabien PicaudRan LiFabio AcerbiRan LiaoFabio ArenaRan LiuFábio AzevedoRan Novitsky NofFabio BisegnaRana Zia Ur RehmanFabio BottaRandolph KirkFabio CaraffiniRanesh Kumar NahaFabio CassanoRangeet MitraFabio D’AndreagiovanniRanil GurusingheFabio GarziaRanjit ShresthaFabio Henrique PereiraRanjith LiyanapathiranaFábio Henrique Rojo BaioRanko HeindlFabio La ForestaRanko Zotovic StanisicFabio LecceseRanniery MaiaFabio Luca BonaliRao FuFabio MaggioRaouf Senhadji-NavarroFabio ManfrediniRaoul NigmatullinFabio Mazzariol SanticiolliRaoul ReiserFábio MendonçaRaphael DeltombeFabio MennaRaphael HorvathFabio MottolaRaphaël KhouryFabio NarducciRaphaël TurcotteFabio PareschiRaphael VallonFabio PatroneRaphael ZacconeFabio PrincipatoRaquel Cama-MoncunillFabio RadicioniRaquel CervigónFabio RizzoRaquel ConceiçãoFabio RossiRaquel De Los ReyesFabio SaliceRaquel Leirós-RodríguezFabio SartoriRaquel López HerederoFabio SauliRaquel Martínez EspañaFabio SavoldiRaquel MayordomoFabio TangoRaquel MontesFabio ViolaRaquel SanchisFabio ZobiRasa PauliukaiteFabiola SpolaorRashid AliFabrice RetiereRashmi Sharan SinhaFabrice SthalRasmus RettigFabrizio BalducciRasmus RiviniusFabrizio Di PasqualeRasoul Shalchi AlishahFabrizio FioriRastislav PirnikFabrizio GabrielliRatko GrbićFabrizio GranelliRaúl Alberto Ponce RodríguezFabrizio MarraRaúl Alcaraz MartínezFabrizio PalmaRaul Ceretta NunesFabrizio PiergentiliRaul Cristian RomanFabrizio RoccaforteRaul Fernandez-GarciaFabrizio StesinaRaul FizesanFadi MuheidatRaul Igual CatalanFagui LiuRaúl Jiménez-NaharroFahad Manzoor SiddiquiRaúl Marticorena-SánchezFahimeh MohagheghianRaúl MontoliuFahmi KhalifaRaul OnetFaisal AlfouzanRaúl ParadaFaisal JamilRaúl Parada MedinaFaisal Mohd-YasinRaul RabinoviciFaizan MehmoodRaúl SuárezFakhroddin NazariRaul-Cristian RomanFalin WuRavi Ancil PersadFaming HuangRavi Kumar CheedaralaFan DangRavi Reddy ManumachuFan GaoRavil MuhamedyevFan JiangRaviraj ThakurFan LiRawaz KurdaFan WuRawil FakhrullinFan YeRaymond K. ZhaoFang ChengRaymundo Buenrostro-MariscalFang LiuRayya A. Al-BalushiFangchen FengRayyan ManwarFangfang JiangRay-Yeng YangFangfang ZhangRaza QaziFangjie YuRazaque AbdulFanglue ZhangRazvan BobocFangmin SunRazvan BogdanFangmin XuRazvan CraciunescuFangning HeRazvan D. TamasFangwen YuRăzvan Gabriel BobocFangzhao ZhangRazvan Ioan PacurarFangzhou LiuRăzvan RughinișFania PalanoRazvan SerbuFanli MengRebeca Jiménez-PérezFanny SpagnoloRebeca P. Díaz RedondoFaouzi DerbelRebecca A. StatesFapeng YuRedha TaiarFa-Qiang WangRedmond R. ShamshiriFaranak ShamsafarReed GurchiekFarbod KhameneifarReem HannaFares Al-ShargieRegine FrankFares M’zoughiRehan SiddiquiFarhad PourpanahRehmat UllahFarhan AminReik DonnerFarhan ChowdhuryReinaldo Martinez PalharesFariba GoodarzianReiner JedermannFarid OrudzhevReiner KriestenFarida El-DarsReinhold HerschelFariza SabrinaRekha GuchhaitFarkas AndreaRem CollierFarkhod MakhmudkhujaevRemedios Lopez-LiriaFarman AliRemigiusz BąchorFarman UllahRemigiusz RajewskiFarong GaoRemous-Aris KoutsiamanisFarooq AdnanRemus BradFaroq AwinRemus PravalieFarouk HammamiRen Cheng JinFarshad Moradi KashkooliRen CooperFaruk RicoRenaldas UrnieziusFarzad FereidouniRenata Borovica-GajicFarzad TashtarianRenata ČinčikaitėFarzad Zangeneh-NejadRenata Dondajewska-PielkaFarzan AkbaridoustRenata GržićFarzin PiltanRenata KarpiczFasheng WangRenata Kelly MendesFatemeh AlidoostRenata Lopes RosaFatemeh HassanipourRenata MarkowskaFatemeh RezaieRenata SistoFatih AkkoyunRenato César Dos SantosFatima Amer Jid AlmahriRenato GiacominiFatima SalahdineRenato HenriquesFatima Zohra BenhaloucheRenato MiyagusukuFatimah KhalafRenato Sobral MonteiroFatos XhafaRencheng SongFaucher CyrilRendy ThamrinFausta FiorilloRené M. RossiFaustino WahaiaReneta BarnevaFausto FerreiraRen-Hung HwangFausto FiorilloRenlong HangFausto Orsi MedolaRenxin WangFausto Pedro García MárquezRenyuan QinFausto TucciRenzo CapitaniFavio-Andrés Pavas-MartínezReuben BurchFayez Tarsha KurdiRevannath NikamFazal MuhammadRex TanFazekas AttilaReza ShahbazianFazio RobertoReza ShahidiFederica DegnoReza TafreshiFederica MaiettiRhodri SmithFederica MarcolinRiad MokademFederica PaolicelliRicardo Alexandre Reinaldo De MoraesFederica PiccirilliRicardo Ambrocio Ramirez-MendozaFederica VerdiniRicardo Augusto Rabelo OliveiraFederico BecattiniRicardo Carmona-GalánFederico BolelliRicardo CordobaFederico CugurulloRicardo DalagnolFederico DiosRicardo Escobar-JiménezFederico GalliRicardo FerreiraFederico Grasso ToroRicardo JimenezFederico KaragulianRicardo KamikawachiFederico MorettiRicardo OliveiraFederico MoroRicardo PereraFederico PittinoRicardo PiedrahitaFederico RoggioRicardo RabeloFederico ScurtiRicardo Ron-AngevinFederico TascaRicardo SantosFei DingRicardo VardascaFei FangRicardo VergazFei FeiRicardo VinuesaFei HaoRicardo ZednikFei HuRiccardo BernardiniFei KangRiccardo CarotenutoFei LiRiccardo ColellaFei LiuRiccardo De BenedictisFei LuRiccardo De GaudenziFei QiaoRiccardo GiubilatoFei QinRiccardo MandriotaFei SongRiccardo MisciosciaFei WangRiccardo MonicaFei XingRiccardo OrtaleFei YuanRiccardo PalamàFeifei YangRiccardo ZuccaFeiyan HuRichard C. DowellFeiyun CuiRichard Demo SouzaFeldheiser AarneRichard DeweyFeldshtein EugeneRichard DvořákFelice LorussoRichard FunkFelipe Augusto Pereira de FigueiredoRichard G. TrohmanFelipe CostaRichard Godinez TelloFelipe Da Rocha HenriquesRichard GreenwaldFelipe Gómez-CubaRichard H. SandlerFelipe JiménezRichard HauerFelipe José AidarRichard HopperFelipe OrduñaRichard J SheridanFelipe Orihuela-EspinaRichard KlemmFelipe ToledoRichard SegallFelipe Trujillo-RomeroRichard SouzaFelipe Vannucchi De CamargoRichard W. BohannonFelisberto PereiraRichard WatsonFelismina T.C. MoreiraRick SturdivantFelix AlbuRick Y.C. KwanFelix BiermannRidwan AlamFelix Escalona MoncholiRiëm El TahryFélix Javier Jiménez-JiménezRih Teng WuFélix Marcos TejedorRiheng JiaFelix ScholkmannRiichi MurayamaFelix WeihsRijuta GaneshFemi J. OlorunnijiRikiya TakeuchiFen LinRikke GadeFeng ChenRiko ŠafaričFeng Chi HsuRimas JaneliukstisFeng DingRinkaj GoyalFeng GanRishikesh PandeyFeng GaoRita Bacelar FigueiraFeng HongRita Catalina Aquino CaregnatoFeng JuRita FigueiraFeng KeRita Girão SilvaFeng LiRita M. KissFeng LinRita StagniFeng LingRitesh ChimoriyaFeng LiuRitesh Kumar SinghFeng NiuRitesh VyasFeng QianRitu MalikFeng SunRituparna ChakiFeng WeiRizwan Ahmed KhanFeng XiaoRizwan Ali NaqviFeng YuanRizwan Aslam ButtFeng ZhengRoald M. TiggelaarFengchao XiongRoald OtnesFeng-Cheng ChangRoana De Oliveira HansenFeng-Cheng LinRob F. RemisFengchun TianRob WijnhovenFenggang SunRobert A. CohenFenghe WuRobert A. ConvissarFenghua LiRobert A. MicklerFengming LiRobert BarańskiFengqiang LiRobert BestakFengqiu XuRobert BurdukFeng-Tsun ChienRobert CastillaFengxia SuRobert CatenaFeng-Yih YuRobert ChodorekFengyu LiRobert CrowtherFengyuan LiuRobert CzerwinskiFengzhong QuRobert DemeterFenu GianfrancoRobert DickinsonFerdinando Di MartinoRobert DyjaFerdous SohelRobert FrischholzFereidoun H. PanahiRobert Gabriel LupuFerenc SzodraiRobert HendersonFereydoun AghazadehRobert Konrad SzymczakFermi Guerrero-CastellanosRobert KrewinkelFermín BañónRobert KubinaFernanda FerreiraRobert LiKamWaFernanda RodriguesRobert M. NowakFernando AlmeidaRobert MilewskiFernando Auat-CheeinRobert MingeszFernando BoavidaRobert MroczyńskiFernando Castañ RomeroRobert NewcombFernando CastañoRobert NowakFernando ChouzaRobert NusterFernando De La Prieta PintadoRobert OleniaczFernando De Souza CamposRóbert OlšiakFernando ElyRobert P. JohnsonFernando FeijooRobert PanowiczFernando FerreiraRobert PaskenFernando GarramiolaRobert PhilippFernando Henrique MagalhãesRobert R. SattarovFernando L. TeixeiraRobert RoczniokFernando M. Araujo-MoreiraRobert RusinekFernando Manuel Lourenço MartinsRobert S. BradleyFernando Quesada-PereiraRobert SchmittFernando Ramos MartinsRobert SitnikFernando Rangel De SousaRobert SotoFernando Reinaldo RibeiroRobert SousaFernando Sánchez LasherasRobert SzczepanekFernando Vaquerizo-VillarRobert T. MenziesFernando Vidal-VerdúRobert TomkowskiFerran MartinRobert UlewiczFerreira De Lucena Junior VicenteRobert WatsonFidel Núñez-RamírezRobert WeberFilip HolikRobert WeserFilip MaksimovicRobert ŽupanFilip ŠuligojRoberta CalmoFilipa JoãoRoberta D’AurelioFilipa SeabraRoberta GasparroFilipa SousaRoberta PeregoFilipe AlvesRobert-Alexandru DobreFilipe Arroyo CardosoRobertas DamaševičiusFilipe BarataRoberto Alejo EleuterioFilipe Manuel ClementeRoberto BellasioFilipe NevesRoberto BernasconiFilipe Neves Dos SantosRoberto CannataroFilipe PortelaRoberto Carlos Ambrosio-lazaroFilipe SilvaRoberto Casado-VaraFilippo BastianiniRoberto Celio Limao De OliveiraFilippo BattagliaRoberto ColasantiFilippo BergamascoRoberto CorizzoFilippo BiondiRoberto CorvajaFilippo BrighinaRoberto Di MarcoFilippo CostaRoberto FrancesconFilippo D’IppolitoRoberto G. CitarellaFilippo GambellaRoberto G. ValentiFilippo Giammaria PraticòRoberto Giovanni Ramírez-ChavarríaFilippo MantovaniRoberto Li VotiFilippo PaganelliRoberto López-ValcarceFilippo PisanoRoberto MacalusoFilippo PoltronieriRoberto Merino-MartinezFilippo UbertiniRoberto MiorelliFilippos GiannakasRoberto NebuloniFilippos VallianatosRoberto OpromollaFiliz Bektas BalcikRoberto OroseiFiliz YesilkoyRoberto PaianoFinbarr MurphyRoberto PaolesseFionn MurtaghRoberto PeronFiras HassanRoberto PierdiccaFiras KobeissyRoberto RaffaeliFirdous A. ShahRoberto RomeoFirooz SalamiRoberto S. NettoFisseha M. AlemayehuRoberto SabatiniFitri TrapsilawatiRoberto SaiaFlávia Aparecida GraçaRoberto TagliaferriFlavia CausaRoberto TeghilFlavia DelicatoRoberto TetiFlavien ValensiRoberto ValentinoFlavio BalsamoRoberto Vincenti GattiFlavio BorfecchiaRobin DobosFlavio CanaveroRobin GerzaguetFlavio CirilloRobyn JonesFlavio EscribanoRoc BerenguerFlavio EspositoRoc MeseguerFlávio FigueiraRocco AlaggioFlavio Henrique VasconcelosRocco CitroniFlavio LupiaRocco FazzolariFlavio PiccoliRocco FurferiFlavio ShimizuRocco HaaseFlavio ZabiniRocio B. DominguezFlaviu Mihai Frigura-IliasaRocio Canovas MartinezFlaviu TabaranRocío Josefina Pérez De PradoFlor G. Ortíz-GómezRocio Sanchez-MonteroFlorenc DemroziRodica Elena IonescuFlorence NyssenRodica HolonecFlorent JangalRodica VoicuFlorent PouxRodica-Mariana IonFlorentina-Daniela MunteanuRodolfo HaberFlorian B. PokornyRodolfo MeneguetteFlorian Ion Tiberiu PetrescuRodolfo Miranda PereiraFlorian KirchschlagerRodolfo RedaFlorian KlämpflRodolphe HeydFlorian KölblRodrigo A. A. MunozFlorian LipsmeierRodrigo BragaFlorian MetzgerRodrigo Colnago ContrerasFlorian MichaudRodrigo Filev MaiaFlorian PapeRodrigo Gomes Da RosaFlorian SchaffRodrigo GonzalezFlorian SigenegerRodrigo Martín De San AgustínFlorin AlexaRodrigo OyonarteFlorin CovaciuRodrigo RighiFlorin Doru HutuRodrigo Rocha SilvaFlorin GirbaciaRodrigo SalasFlorin Molnar-MateiRodrigo Sanches MianiFlorin OnigaRodrigo SantosFlorin PopaRoemi FernandezFlorin PostolacheRogelio Bustamante-BelloFlorin SalaRogelio De J. Portillo-VélezFlorin Valentin RuscaRogelio Gónzalez-GónzalezFlorina CojocaruRogelio Hasimoto-BeltranFlorina TeodorescuRoger BilhamFlorina UngureanuRoger C. LoweFlorin-Constantin MihaiRoger M. KrzyżewskiFocke ZiemssenRoger Pissard-GibolletFodor MariettaRoger ResminiFoivos MichelinakisRogerio Atem De CarvalhoFokko WieringaRogério DionísioFolea SilviuRogerio ManicaFortunato C. DualibeRohan NagareFortunato PezzimentiRohan SomanFosco M. VeselyRohit MishraFossataro CarlottaRohit VermaFotios GioulekasRoi MéndezFotis MavromatakisRoja EsmaeeliFow-Sen ChoaRok DolenecFoziyeh SohrabiRok VrabičFranc JagerRoland BejjaniFrance Le BihanRoland BouffanaisFrancesc EstranyRoland ChapuisFrancesc SebeRoland ValckeFrancesc WilhelmiRoland ZügnerFrancesca AntonucciRolando HerreroFrancesca BorghiRolf BeckerFrancesca BugiottiRomain JacobFrancesca CordellaRoman A. ZakoldaevFrancesca CovaRomán Alcides Lara-CuevaFrancesca Dalia FaraciRoman BauerFrancesca LionettoRoman DanelFrancesca LoiaRoman FediukFrancesca LonettiRoman FedorenkoFrancesca MalvanoRoman ForkeFrancesca ManniRoman GrillFrancesca NardelloRoman HolovchakFrancesca PennatiRoman KolcunFrancesca R. BertaniRoman KrzanowskiFrancesca RossiRoman KubackiFrancesca RussoRoman KuscheFrancesca VenneriRoman MajorFrancesca VenturiniRoman MaršálekFrancesco AlettaRoman RegulskiFrancesco Antonio ArchettiRoman RousseauFrancesco BanterleRoman ShultsFrancesco BeritelliRoman SkidanovFrancesco BertocciRoman SotnerFrancesco BianconiRoman SzewczykFrancesco BrandaRoman TrachFrancesco CafaroRoman TrochimczukFrancesco CappuzzelloRoman TsvetkovFrancesco Carlo MorabitoRoman V. KlyuevFrancesco CastellaniRoman ViterFrancesco CauteruccioRoman WyrzykowskiFrancesco CenturelliRoman Y. PishchalnikovFrancesco ChitiRomana SchirhaglFrancesco ClementiRomolo MarcelliFrancesco CoscoRomuald BoucheronFrancesco D’AmatoRomualdas BaušysFrancesco De LeoRomualdas NavickasFrancesco De NataleRomulus CostacheFrancesco De PaceRomulus TerebesFrancesco Ernesto PontieriRonald LeeperFrancesco FacchiniRonald RobertsFrancesco FaitaRonan PaugamFrancesco FerracutiRone Ilídio Da SilvaFrancesco FilippiniRoneel SharanFrancesco FusoRong RanFrancesco Giacinto LavaccaRong YinFrancesco GranataRong-Ho LeeFrancesco GuidiRong-hua HuanFrancesco GuzziRonghua MaFrancesco LamonacaRonghui ZhanFrancesco LelliRongpeng LiFrancesco LongoRongrong LiuFrancesco MaitaRongyong HuangFrancesco MarinelloRonnie O. Serfa JuanFrancesco MartelliRoohallah AlizadehsaniFrancesco P. ChieteraRoope RaisamoFrancesco Paolo LovergineRoozbeh Abedini-NassabFrancesco PascaleRoozbeh AtriFrancesco PotortìRoque A. Osornio RíosFrancesco QuochiRory CooperFrancesco RaffaRory O’SullivanFrancesco RennaRosa Rio‐BelverFrancesco RossellaRosalba GaudiusoFrancesco RuffinoRosalinda InguantaFrancesco SantoniRosalyn L. PutlandFrancesco ScardullaRosalynn NankyaFrancesco StilloRosamaria SalvatoriFrancesco VatalaroRosana Alves DiasFrancesco VerdeRosanna ViglialoroFrancesco VilleccoRosaria RuccoFrancesco VisentinRosario ArjonaFrancine KriefRosario FedeleFrancis BennetRosario García-GiménezFrancis J OsongaRosario OlivaFrancis Kalloor JosephRosario Porras-AguilarFrancis M BolandRosario Schiano Lo MorielloFrancis M. GroverRoshan DsouzaFrancis MulolaniRoshan JosephFrancis WyffelsRoshan KhadkaFrancisco Alburquerque-SendínRosilah HassanFrancisco Alexandre A. SouzaRosolino VaianaFrancisco Alonso SarríaRoss CheritonFrancisco António Bucho CercasRoss ClarkFrancisco AvilaRoss DarnellFrancisco AznarRoss N. GillandersFrancisco Beltran-CarbajalRostam Affendi HamzahFrancisco BerralRoufei ChenFrancisco BulnesRoumen KirovFrancisco CabreraRoussin Lontio FomekongFrancisco CercasRoxana RadvanFrancisco ChinestaRoxana-Mariana BeiuFrancisco ContrerasRoxanne RadpourFrancisco CruzRoy B. V. B. SimorangkirFrancisco Cuevas De La RosaRoya NasimiFrancisco Del Cerro VelázquezRuan Delgado GomesFrancisco Eugenio Potestad-OrdóñezRubén Carlos AcevedoFrancisco Fernández ZacaríasRubén Martín-ClementeFrancisco Fernández-NavarroRuben MedinaFrancisco Gallegos-FunesRuben PauwelsFrancisco GámezRubén Picó VilaFrancisco Garcia-LagosRubén San-SegundoFrancisco Gomez-DonosoRuber Hernández-GarcíaFrancisco GonzálezRucha DeshpandeFrancisco Haces-FernandezRuchire Eranga WijesingheFrancisco J. Aparicio-NavarroRuchire WijesingheFrancisco J. Díaz-OteroRüdiger GerdesFrancisco J. Ferrero MartínRüdiger RuppFrancisco J. González-CastañoRudinei GoularteFrancisco J. MartinezRudnei Dias Da CunhaFrancisco J. MedranoRudnik KatarzynaFrancisco J. NavarroRudolf JanosFrancisco J. Rodriguez-PulidoRudolf UrbanFrancisco Javier Cárcel-CarrrascoRudolf VerdaasdonkFrancisco Javier CardenalRuei-Sung YuFrancisco Javier Gimeno BlanesRueter DirkFrancisco Javier González-CañeteRuey-Ching TwuFrancisco Javier Mesas CarrascosaRuey-Kai SheuFrancisco Javier OtamendiRuggero LanotteFrancisco José García-PeñalvoRuggero ReggianniniFrancisco Jose Mora-GimenoRugved LikhiteFrancisco López-HuertaRuhollah Taghizadeh-MehrjardiFrancisco Luna-PerejónRui AntunesFrancisco M. PadillaRui BaiFrancisco Manuel Arrabal-CamposRui BernardesFrancisco Manuel MelgarejoRui CamposFrancisco Manuel Melgarejo MeseguerRui Costa MartinsFrancisco Manuel Moreno-PinoRui DilãoFrancisco Maroto-MolinaRui DinisFrancisco MarquesRui DongFrancisco Martínez-ÁlvarezRui Fonseca-PintoFrancisco MedinaRui LiFrancisco Miguel Ribeiro Proença BrójoRui LinFrancisco MolinaRui MinFrancisco Molina MartelRui Pedro JuliãoFrancisco MorenoRui Pedro LopesFrancisco Nieto EscámezRui SilvaFrancisco Perez-PinalRui Silva MoreiraFrancisco PizarroRui SunFrancisco RegoRui Vasco SilvaFrancisco Ronay López-EstradaRui YangFrancisco-Javier Moreno-MuroRui YuanFranciszek SeredynskiRui ZhangFranck BadetsRui ZhaoFranco FrattolilloRuifeng LiFranco FurgiueleRuihai DongFranco MeggioRuijie WangFranco PaveseRuijun LiFranco TedeschiRuinian JiangFrancois NicolasRuiqing ShenFrançois OuelletteRuishu F. WrightFrancois VarrayRuisong HanFrank BoochsRuitao YangFrank DeinzerRuixiang BaiFrank LiebischRuiyu LiangFrank MelandsoRujie HeFrank MissellRukhsana RubyFrank P. MissellRuman BanerjeeFrank R. IhmigRune SchlanbuschFrank RaischelRunmin CongFrank SlyneRunqi ChaiFrank T. FergusonRunqiang ZengFrank Van EerdenburgRunze LiFrank WitmerRuocan QianFrank-Michael MatysikRuochen HuangFrank-Peter SchillingRuochen LuFrantisek BrumercikRuopeng SunFrantišek KumhálaRuoxi SunFrantišek LopotRuoxiu XiaoFrantišek PollákRuoyu SuFrantišek UherekRupesh ShresthaFrantišek ZbořilRuriko HatadaFranz Konstantin FussRuriko YoshidaFranz WaldnerRushi LanFreddy A. LucayRushit DaveFrédéric ChapelleRuslan E. AsfinFrédéric CointaultRussell FarrugiaFrédéric DehaisRusso PaoloFrédéric DumurRustam KhabibullinFrédéric GillotRüstem KeçiliFrederic MarinRuszczak BogdanFrederic MelinRūta GerasimaitėFrédéric Nicolas DaussinRutger OsterthunFrédéric VanderhaegenRuth MacKayFrederica VitiRuth ShinarFrederick T. SheldonRuud G. J. MeulenbroekFrederico Miguel SantosRuxandra F. OlimidFrederik BeuthRuxandra StoeanFrédérique SeylerRuxandra TapuFredrik JohanssonRuya XiaoFredrik KarlssonRyad BouzouidjaFredrik OlssonRyad ZemouriFreek ArieseRyan ChapmanFridolin WildRyan GibsonFridon ShubitidzeRyan MattfeldFrieder C. KrafftRyan NiemeierFriedrich HaagRyan RamirezFrits Van Der EerdenRyan T. WhiteFrouke HermensRyan TerrienFu LiRymantas Jonas KažysFuchuan DuanRymarczyk TomaszFuentes-Aguilar RitaRyo NatsuakiFufei PangRyohei MisumiFuhua ChenRyoji KuritaFujian MaRyoma MorigakiFujiang LinRyou OhsawaFu-Kang WangRyou TanakaFulin LuoRyszard JachowiczFumihide KojimaRyszard PawlakFu-Min ChangRyuichi OhtaFumin ZhangRyuji HamamotoFuming SunRyuki SatoFumio NaritaS. Zohreh HomayounfarFumio YamazakiSabato MelloneFumiya IidaSabina LicenFupeng WangSabina MaraffiFusang ZhangSabina MerloFusheng ZhaSabina SzymoniakFutai ZhangSabino MaggiFuwen HuSabita PanickerFuxin DuSabri AllaniFuxing GuSabrina DemarieFuyuan XiaoSabrina Di MasiFysarakis KonstantinosSabrina LeoG. G. Md. Nawaz AliSabrine KherijiGábor KertészSabyasachi MondalGabor KosaSachin KumarGábor MocsárSachin NavaleGabor SoosSachin SharmaGabor SziebigSachit MahajanGábor VértesySadegh SamadiGabriel A. E S. FerrazSadeq SalehGabriel A. LopezSadeque Reza KhanGabriel D. PeckhamSadko MandžukaGabriel Dias RodriguesSadok RezigGabriel Domínguez-MaldonadoSae-Bae NapaGabriel FalcaoSaed MoradiGabriel FedorkoSaeed Hamood AlsamhiGabriel Gonzalez-SernaSaeed KhakiGabriel HancuSaeed M. BamatrafGabriel Lucian RaduSaeed M. QaisarGabriel MurariuSaeed MahmoudpourGabriel NeaguSaeed MouloodiGabriel Nicolae PopaSaeed NosratabadiGabriel OlteanSaeed OlyaeeGabriel Ramos-OrtizSaeed RehmanGabriel Sanchez-PerezSaeed ShavvalpourGabriela CovatariuSaeid NiazmardiGabriela Ioana ProşteanSaeid Pourroostaei ArdakaniGabriela KuncováSagar JinachandranGabriela PaunescuSagi FilinGabriela RutkowskaSagrario Martín-AragónGabriela Statkiewicz-BarabachSahaj SaxenaGabriela Ventura SilvaSahameddin Mahmoudi KurdistaniGabriel-Alexandru ConstantinSahand PirouzpanahGabriele BertagnoliSahraoui DhelimGabriele ButtafuocoSai G.S. PaiGabriele Di BlasioSai HuangGabriele FreniSai Wei YangGabriele MagnaSaid BenissaGabriele Maria LozitoSaid MunirGabriele MeoniSaid QuqaGabriele NavaSaiful BariGabriele PapadiaSaim MemonGabriele S. De BlasioSaima QureshiGabriele ScaliaSaiqi TianGabriella BalestraSajad JamshidiGabriella BognárSajad MohamadzadehGabriella CasalinoSajad SabziGabriella GiganteSajeesh KumarGabriella OlmoSajid NisarGabriella TamburroSajjad HussainGaddi BlumrosenSakdirat KaewunruenGaël KermarrecSakib K. PathanGael SebaldSalah KhanGaelle LissorguesSalaheldin ElkatatnyGaëtan ReySalam Al-SabahGaetano BellancaSalam IbrahimGaetano D. GargiuloSalam KhamasGaetano De LucaSalau BelloGaetano GargiuloSaleh AbujaradGaetano PalumboSaleh AlyGaetano QuattrocchiSaleh MohammadGaetano RanieriSalicone SimonaGaetano VaccaSalik Ram KhanalGaffar HossainSalil KaripottGaia PavoniSalim LamineGaige WangSally McCleanGai-Ge WangSalma ElsayedGail SchofieldSalman Ahmadi-AslGail WhitelawSalman AliGaitan Nicoleta CristinaSalman I. SiddiquiGajanan GhodakeSalua HamazaGajdoš Kljusurić JasenkaSalvador AlcarazGajendra Pratap SinghSalvador Barrera FigueroaGaku ImamuraSalvador Dueñas CarazoGalatanu Catalin DanielSalvador J. JaimeGalien GrosjeanSalvador Mendoza-AcevedoGalina B. SlepchenkoSalvador MorenoGalina BogdanovaSalvatore A. PullanoGalina V. KurlyandskayaSalvatore CartaGalya Georgieva-TsanevaSalvatore DanieleGamini AriyawansaSalvatore Falanga BolognesiGanapati BhatSalvatore GrazianiGanbayar BatchuluunSalvatore MusumeciGandhimathi VelusamySalvatore PonteGanesh KumarSalvatore SessaGanesh Kumar SrinivasanSalvatore SurdoGanesh R. NaikSalvatore TedescoGanesh SarataleSalvina GaglianoGang ChenSalvo MarcuccioGang ChengSam AmiriGang KouSam LoflandGang LiSam ReisenfeldGang MeiSamad SepasgozarGang MengSamadhan Bhaulal PatilGang WeiSaman HedjaziGang XiongSamaneh KouchakiGang XuSamantha DugelayGang YaoSambor GuzeGangil ByunSambuddha GhosalGangqiang ZhaSameer Chakravarthy VedulaGao HuSameera WickramasingheGaoang WangSamer KharroubiGaofeng QuanSamer KhouriGaoge HuSami BarmadaGaoliang PengSami MyllymaaGaoyang ShanSami NikkonenGarbiñe OlabarríaSami ObaidGareth ReesSamir HachourGarry AllisonSamir KhatirGary AtkinsonSamir MalakarGary HooperSamir MustaphaGary M. RothenbergSamira BriongosGary StoreySamira NaghdiGaspare GiovincoSamntha MacchiGaurab DuttaSamo StaničGaurav ChoudharySampsa VanhataloGaurav SharmaSamuel AgbrokoGautham PrasadSamuel Deslauriers-GauthierGavriil George ArsoniadisSamuel Kofi ErskineGbanaibolou JomboSamuel LawmanGe HanSamuel Montejo SánchezGe JinSamuel Morillas GomezGeb W. ThomasSamuel Ortega SarmientoGeetesh K. MishraSamuel SilvaGeetika AggarwalSamuel X. ShiGeir StorvikSamuel Xavier-de-SouzaGelayol GolcarenarenjiSamuele De PetrisGelu OnoseSamuele GelliGema Prats BoludaSamy ShabanGemechu GarumaSana SyedGemine VivoneSana Ullah JanGemma TintiSanath Kumar SathyachandranGen LiSanathanan MuttikulangaraGenggeng LiuSanaz JarolmasjedGeng-Shi JengSanaz KavianpourGengyan ZhaoSanaz PournajafGennady EvtugynSanaz Rahimi MoosaviGennady I. GrigorievSanbo QinGennady ZebrevSandeep K. ChaudhuriGennaro Nicola BifulcoSandeep K. SinghalGennaro VessioSandeep KumarGeoff FinkSandeep SharmaGeorg Christoph BrunauerSandeep SonyGeorg FischerSander Oude ElberinkGeorg RillSandhiran PatchayGeorg RoseSandip DuttaGeorg SchildbachSandor FreyGeorge A. PapakostasSándor SzénásiGeorge AdamSándor ValkaiGeorge C. AlexandropoulosSandra BravoGeorge Cunha CardosoSandra CanoGeorge D. ManolisSandra DrusováGeorge Dan MoisSandra DudleyGeorge E. TsekourasSandra GamaGeorge E. ViolettasSandra García-GallegoGeorge FlorosSandra GrabowskaGeorge GiakosSandra MogoGeorge GrouiosSandra RodriguesGeorge K. VarotsosSandra RogerGeorge LazaroiuSandro Da Silva CamargoGeorge ManisSandro FiorettiGeorge MargetisSandy CochranGeorge MelillosSanford MeekGeorge MiloshevSang Hun LeeGeorge N. ReekeSang Hyun LeeGeorge NikitasSang Joon ParkGeorge OguntalaSang Jun LeeGeorge PapakostasSang M. WonGeorge PonchakSang Won BaeGeorge SuciuSangchoon KimGeorge TsaramirsisSangdae KimGeorges AlquiéSangeet SahaGeorges KaddoumSangGon LeeGeorgia SakellariSangho BokGeorgiana CodinaSanghoon LeeGeorgiana SimionSangita DasGeorgios A. KyriakouSangita PalGeorgios BouloukakisSang-Jae KimGeorgios ChristopoulosSangjin ByunGeorgios CtistisSang-Jin ChoiGeorgios D. PanosSangjo ChoiGeorgios DimitrakopoulosSangkil KimGeorgios DrakopoulosSangman MohGeorgios E. ArnaoutakisSangmin AnGeorgios E. StavroulakisSangmin LeeGeorgios GeorgakisSangrok JinGeorgios GiarmatzisSangsoon LimGeorgios HadjidemetriouSang-Woo SeoGeorgios KaralekasSang-Yoon ChangGeorgios KatsikisSanislav TeodoraGeorgios KavallieratosSanja Mahovic PoljacekGeorgios KostopoulosSanja MartinezGeorgios LeontidisSanja RadosavljevicGeorgios MylonasSanjay KumarGeorgios N. TsigaridasSanjay Kumar BiswashGeorgios P. MastrotheodorosSanjeev ArulampalamGeorgios PriniotakisSanjeev Kumar SagwalGeorgios SamakovitisSanjeev TambeGeorgios SpathoulasSanju GuptaGeorgios TsaramirsisSanket AwateGeorgios TsigaridasSanoj RejinoldGeorgios TsoumanisSansit PatnaikGeorgy FedorovSante Francesco RendeGe-Peng JiSanthakumar SampathGera MeetaSantiago CepedaGerald T. GrantSantiago Felici-CastellGéraldine E. MerleSantiago GutiérrezGeraldo CernicchiaroSantiago PuenteGeraldo Pereira Rocha FilhoSantiago Sánchez-SolanoGérard ChalhoubSanto OrlandoGerard ZamoraSantos Sanchez-CambroneroGerardo Alguacil De La BlancaSantosh KumarGerardo ArangurenSantosh Kumar PaidiGerardo FloresSantosh PitlaGerardo Ulises Diaz-ArangoSantosh ShresthaGerasim Vladimirovich KrivovichevSantosh SubediGerasimos BastasSantoso WibowoGerasimos PagiatakisSaptarshi MukherjeeGerasimos TheotokatosSaptarshi SarkarGerd DobmannSaptarshi SenguptaGerd ReisSara CasacciaGerd SchollSara ComaiGergely LautnerSara Díaz CardellGerhard Aernout SomsenSara FerreiraGerhard LeitnerSara Gonizzi BarsantiGerhard MucheSara JayousiGerhard MullerSara MocciaGerhard PaarSara StančinGerhard PölzlSara StolyarovaGerhard SteinbeckSara TodeschiniGerhard Wilhelm WeberSara TombelliGerhard ZangerlSarah AzimiGermán Carro FernandezSarah HubbardGermano M. VeigaSarah R.B. KingGert KootstraSarah RingerudGhada KhoribaSarah TonelloGhada ZamzmiSaranraj KaruppuswamiGhaith KhalilSarath VijayakumarGhaleb HoblosSarfaraz AdnanGhasem AbdiSarita Keski-SaariGhasem SargaziSarita Mazzini BruschiGhazal RouhafzaySarthak PathakGheorghe N. PopescuSarunas GrigaliunasGheorghe NagitSarunas MeskinisGheorghe NechiforSaša ĆukovićGheorghe Nicusor PopSasa DjuricGheorghe SebestyenSasa MrdovicGheorghe ZahariaSasa SladicGheorghe-Daniel VoineaSascha EichstädtGheorghe-Eugen SubtireluSascha FleerGhiseok KimSascha PreuGholam Hossein RoshaniSaskia Kraft-BermuthGholamreza MohammadpourSasya MadhurantakamGhufran AhmedSatheesh ChandranGhulam HussainSathishkumar SamiappanGia Khanh TranSathiyaraj KandhasamyGiacinto BarresiSathwik S. KasyapGiacomo CabriSathyachandran Sanath KumarGiacomo CapizziSatish Mahadevan SrinivasanGiacomo CapponSatoru TsujiiGiacomo GenoveseSatoshi KuriharaGiacomo MuntoniSatoshi MatsumotoGiacomo ViccioneSatoshi SagaGiada MagniSatoshi TsujiGiamberto CasiniSatoshi UenoGiampaolo PitruzzelloSatoshi YamaneGiampiero ScortichiniSatyabrata AichGian Bartolo PicottoSatyendra Kumar MishraGian Giuseppe SomaSaugat BhattacharyyaGian Luigi GragnaniSaul DelabridaGian Luigi LibertiSaúl E. Pomares HernándezGian Piero GibiinoSaúl Vallejos CalzadaGiancarla AlbertiSaulius BaleviciusGiancarlo OrengoSaulius JuodkazisGiancarlo RufinoSaurabh SahuGiancarlo SorrentinoSaurabh ShuklaGiancarlo SperlìSaurabh SinghGiandomenico CarusoSaurin PatelGiandomenico SpezzanoSaverio LatorrataGianfranco AvitabileSaverio MaiettaGianfranco ForlaniSavio SciancaleporeGianfranco GagliardiSawyer D. CampbellGianfranco MitacchioneSayan GangulyGiang NguyenSayan RoyGianluca CaparraSayed Pouria TalebiGianluca EspositoSaygin AbdikanGianluca GennarelliSazzadur ChowdhuryGianluca GiuffridaScott A. TrammellGianluca PalliScott AdamsGianluca PercocoScott E. BudgeGianluca RealiScott ThomasGianluca RuffatoScott WhiteGianluca SarriSe Dong MinGianluca TorneseSeahwa WonGianluigi De FalcoSean CaseyGianluigi TiberiSean ElvidgeGianmarco DinelliSean McKeownGianni CarioSebania LibertinoGianni CerroSebastia GalmesGianni D’AngeloSebastian BaderGianni PasoliniSebastian BährGiannis GiannoulisSebastian BerhausenGianvito ScaringiSebastian BöttcherGiedre SabaliauskaiteSebastian BudzanGiedre StreckieneSebastian DahleGiedrius JanusasSebastian DudzikGiernacki WojciechSebastián García GalánGihan MendisSebastian GłowińskiGil GonçalvesSebastian GutierrezGil ShalevSebastian HeglerGilbert De MeySebastian HenselGilbert LimSebastian KappGilberto Anzueto-SánchezSebastian KotGilberto CorsoSebastian MüllerGilberto RiveraSebastian RamacherGilbert-Rainer GillichSebastian ScheurerGil-Jin JangSebastian SchnaubeltGilles BourbonSebastian SeidelGilles LabonteSebastian SteinlechnerGilles LemercierSebastian TomczakGilles ParentSebastian TroiaGilles RoySebastian TschaunerGilson A. GiraldiSebastian WolfGilson Adamczuk OliveiraSebastian ZlotnikGiner Alor-HernándezSebastiano MassaroGinés García-MateosSebastiano MilardoGinés Morales MéndezSébastien CombéfisGino DardanelliSebastien GabouryGino PutrinoSebastien GadalGino SorbelloSébastien LallechereGintautas DaunysSébastien MouchetGintautas DzemydaSebastijan MrakGintautas TamulevičiusSedig S. AgiliGinu RajanSedighi SaeidGioacchino TempestaSeehwan YooGiordano TezaSehwan ChoiGiorgia AgrestiSeid KoricGiorgia FioriSeid Miad ZandaviGiorgia GiovanniniSeigo OhnoGiorgia LiguoriSeiya SuzukiGiorgia SollaiSe-Jin LeeGiorgio BiagettiSejin ParkGiorgio BonmassarSejung YangGiorgio FiccoSekchin ChangGiorgio FuggettaSelda OezkanGiorgio PennazzaSelim SolmazGiorgio PolettiSelma RizvičGiorgio Sebastiano MauroSen QiuGiorgio VilardiSen WangGiorgios MallinisSen YanGiorgos AlevizopoulosSenghan YooGiosue CalianoSenol PiskinGiovanna BucciSenthil Kumar KaruppannanGiovanna ConcuSenthilKumar MohanGiovanna OrsiniSeok Jae ChuGiovanna TranfoSeok-Cheol KeeGiovanni AngiulliSeok-Ho RhiGiovanni ArtaleSeok-Hwan JeongGiovanni Battista RossiSeok-Joo KohGiovanni BertoliniSeonah LeeGiovanni BortolanSeong Jung KwonGiovanni BoschettiSeong Soo A. AnGiovanni BottariSeongah JeongGiovanni BreglioSeongchan KimGiovanni BrunoSeong-Cheol KimGiovanni BucciSeong-Eun MoonGiovanni CabassiSeong-eun YooGiovanni CrupiSeong-Heum KimGiovanni De GasperisSeonghun Hoon KimGiovanni Di DomenicoSeong-Ik HanGiovanni DiracoSeongjin LeeGiovanni Francesco SolitroSeongju ChangGiovanni FusinaSeongkeun ParkGiovanni GaleotoSeong-Kyu KimGiovanni GalloSeongmin HeoGiovanni GiganteSeongMin OhGiovanni LeoneSeong-Tae HanGiovanni LeucciSeongwook LeeGiovanni Luca MasalaSepehr AlizadehsalehiGiovanni MagnoSepideh KhoshnevisGiovanni ManfrediSeptimiu LicaGiovanni Maria SardiSeptimiu MischieGiovanni MatareseSerafeim MoustakidisGiovanni MottolaSerafin Lopez-Cuervo MedinaGiovanni NardiniSerafín Moral-GarcíaGiovanni PalmeriniSerban ChivulescuGiovanni ParagliolaSerban Georgica ObrejaGiovanni PauSerban MezaGiovanni PiccinniSerena DattolaGiovanni PintoreSerge TamariGiovanni SotgiuSergei A. SoldatenkoGiovanni VitaleSergei ChernyiGiraudo AlessandroSergei I. BadulinGirolamo CostanzaSergei KomogortsevGisela Müller-PlathSergei KulinichGisela Pujol VazquezSergei PiskunovGiulia AbateSergei PopovGiulia ButtazzoniSergei SavinGiulia CarusoSergejs KodorsGiulia CisottoSergey A. ButenkovGiulia FracastoroSergey A. ShevchikGiulia LiberatiSergey A. Stel’makhGiulia PanebiancoSergey AleshinGiulia PaparellaSergey BezzateevGiulia RonchettiSergey GudoshnikovGiulia SaccoSergey I. FomenkoGiulia SelvoliniSergey K. AityanGiulio BernardiSergey KobtsevGiulio BiondiSergey KonovalovGiulio CaldarelliSergey KornilovGiulio CottoneSergey KucheryavskiyGiulio D’EmiliaSergey N. ShevtsovGiulio RosatiSergey Pavlovich MorozovGiulio VialettoSergey Pavlovich OsipovGiulio VignoliSergey PirogovGiuseppe AndreoniSergey S. KrasilnikovGiuseppe ArrabitoSergey ShevchenkoGiuseppe ArteseSergey ShevtsovGiuseppe AvertaSergey ShoydinGiuseppe BoccignoneSergey SokolovGiuseppe BombinoSergey V. ZavjalovGiuseppe BrunettiSergey Vadimovich AnakhovGiuseppe CampobelloSergey VinogradovGiuseppe CantarellaSergey ZavadskiyGiuseppe CapobiancoSergey ZhironkinGiuseppe CarboneSergieieva Kateryna LeoidivnaGiuseppe CastorinaSergii BabichevGiuseppe CasulaSergii KushchGiuseppe CattaneoSergio A. Araujo-EstradaGiuseppe CiaburroSergio A. DavidGiuseppe CoratellaSergio CalixtoGiuseppe CovielloSergio CamizGiuseppe D’AlessandroSergio CarratoGiuseppe Del CoreSergio De SalvatoreGiuseppe Di CaprioSergio DominguezGiuseppe Di LeoSérgio Duarte CorreiaGiuseppe Di ModicaSergio Gonzalez-SevillaGiuseppe Di StefanoSergio HernandezGiuseppe Domenico ArrabitoSérgio I. LopesGiuseppe EspositoSergio Ibarra EspinosaGiuseppe FerriSergio L. Stevan, Jr.Giuseppe JurmanSergio López-GarcíaGiuseppe LacidognaSergio Luis Suárez GómezGiuseppe LoprencipeSergio M. O. TavaresGiuseppe Maria LuigiSergio Martin-del-CampoGiuseppe MazzarellaSergio MontenegroGiuseppe MessinaSérgio MoroGiuseppe MinerviniSergio RuggieriGiuseppe ModicaSergio Silva RibeiroGiuseppe MusumeciSergio TorresGiuseppe NittiSergio ValeroGiuseppe PalestraSergiusz ŁuczakGiuseppe PucciarelliSergiusz PatelaGiuseppe QueroSerguei PrimakGiuseppe RiccioSerguei StoukatchGiuseppe SarnèSerhii StepenkoGiuseppe Schirripa SpagnoloSerioja Ovidiu TatuGiuseppe ScottiSeth H. PetersonGiuseppe SpilotroSeung LeeGiuseppe TagliaviniSeung YunGiuseppe Trusso SfrazzettoSeung-Bok ChoiGiuseppe TuriniSeungho JungGiuseppe VaroneSeung-Ho LimGiuseppe ZappalàSeung-Hwan BaeGiuseppina GiniSeunghwan LeeGiuseppina PappalardoSeung-Ik LeeGiuseppina PreziosoSeungkyu RyuGiusy MacrinaSeungmo KimGlauco FontgallandSeung-Nam KimGleifer Vaz AlvesSeungryul BaekGlen CooperSeungwon SongGloria Cerasela CrisanSeungyub LeeGloria CosoliSever-Gabriel RaczGM ShafiullahSeverino Munoz-AguirreGo YamakoSeyed Ali CheraghiGo YamamotoSeyed Ali GhahariGobe HobonaSeyed Ali RezvaniGoeran FiedlerSeyed Amir HoseinitabatabaeiGokarna SharmaSeyed Hamidreza Aghay KaboliGokhan MumcuSeyed Mahdi DarroudiGokulnath ThandavarayanSeyed Mohammad GhoreyshiGolovan Andrey AndreevichSeyed Mostafa Mousavi KahakiGómez Aguilar José FranciscoSeyed Saeid MohtasebiGon Sup KimSeyedabdollah MirbozorgiGoncal CostaSeyedali MirjaliliGoncalo JesusSeyed-Ali Sadegh-ZadehGonçalo MarquesSeyedfakhreddin NabaviGonçalo RodriguesSeyedmohammadreza HeibatiGonçalo SantinhaSeyednima KhezrGong ChengShabnam JabariGong ZhangShabnam Sadeghi EsfahlaniGongjin LanShaddrack Yaw NusenuGongping HuangShah Nawaz BurokurGongping WuShah NazirGonzalo AvariaShahab Eddin JozdaniGonzalo César Gutiérrez-TobalShahab HaghayeghGonzalo De Miguel-VelaShahab Heshmati-alamdariGoorak KwonShahab PashaGopi SamudralaShahab TayebGoran B. MarkovicShahid HussainGoran KosShahinur AlamGoran KrstacicShahood Uz ZamanGoran KvaščevShahram HeydariGoran PoparićShahram MoafipoorGoran TurkShahram PayandehGoran VasiljevićShahram SattarGorazd BombekShahzad AshrafGordon TaoShaibal BaruaGorka VuletićShaila AfrojGorkem MemisogluShailabh KumarGornushkin IgorShailendra RajputGottfried LechnerShakti P.C.Gottfried SchwarzShalei SongGotzon AldabaldetrekuShama IslamGourab Dutta BanikShamik TiwariGovindasamy ManiShan JiangGovindhan MaduraiveeranShancheng CaoGowtham SathyanarayananShancheng YanGrace HwangShangfei SongGraciela Šterpin-ValićShangjie RenGrava CristianShangran XieGrazia BarchiShanhai JinGrazia Giuseppina PolitanoShankargouda PatilGraziella ScandurraShanpu ShenGrazina KorvelShanq-jang RuanGrażyna Kiliańska-PrzybyłoShanshan Gu-StoppelGreenhall JohnShanshan WangGreg J. TallentsShanshan WeiGreg VaughanShantanu PalGrégoire LefebvreShantonu BiswasGregor DonajShaocheng LuoGregor KosShaocheng QuGregor SchieleShaochun DongGregor StrleShaofang GongGregorij KurilloShaohua WanGregorio ProcissiShaohua WangGregoriy KaplanShaohui LiuGregory BarshteinShaojie QiaoGregory BurwellShaojing FanGregory DoumenisShaojuan LuoGregory J. GrosickiShao-Ku KaoGregory MillerShaolin ZhangGregory P. CarmanShaolong KuangGregory PrigozhinShaopeng HuGregory ProvanShaopeng WangGregory Q. WallaceShaoqing HuGregory ReisShao-Yi HsiaGregory SimonsShaqfeh MohammadGreice K.B. Da CostaSharief OteafyGreta FaccioSharifu UraGrigor AngjeliuSharnil PandyaGrigor NikaShashi Kant BhatiaGrigoras GheorgheShaukat Ali MazariGrigorios G. AnagnostopoulosShaul OzeriGrigorios NasiosShaun M. SharpeGrigoris KaltsasShaun WestGrigory Ivanovich DolgikhShawana TabassumGrippa TaïsShawn M. BeaudetteGrover ZuritaShayan AngiziGrzegorz BieszczadShazali OsmanGrzegorz BlakiewiczSheeraz AhmedGrzegorz ChladekShehzad Ashraf ChaudhryGrzegorz DombekSheikh AkbarGrzegorz FiloSheikh Mohammad IdreesGrzegorz FusiekSheikh Shanawaz MostafaGrzegorz J. NalepaShekh Md Mahmudul IslamGrzegorz JanczykShelly VishwakarmaGrzegorz JasinskiShen LiuGrzegorz KarońShen YinGrzegorz KomarzyniecShen YingGrzegorz KopeckiSheng FuGrzegorz KowzanSheng Lih YehGrzegorz KrólczykSheng TanGrzegorz LentkaSheng WangGrzegorz LigusSheng XuGrzegorz Marcin WójcikShengbo ChenGrzegorz MazurShengbo ShanGrzegorz MieczkowskiShengchang LanGrzegorz NalepaSheng-Chi LiuGrzegorz PastuszakSheng-Di LinGrzegorz PsujShenghai YuanGrzegorz RedlarskiShengheng LiuGrzegorz SarwasShenghong HeGrzegorz SkrabalakShenghong YangGrzegorz SobotaShengjia WangGrzegorz StepniakShengjun ZhouGrzegorz ŚwitShengkai ZhangGrzegorz TrybekShengkun XieGrzegorz TrzcińskiShenglin MaGrzegorz WojcikShengnian WangGrzegorz WrzesińskiShengqiang WangGrzegorz ZimonSheng-Sheng YuGu Eon KangSheng-Shih WangGu KangShengxiang GeGuadalupe Esteban Vazquez-BecerraShengyang ChenGuan GuiShengyu DuanGuan XuSheraz AslamGuang YangSherief HashimaGuangbo HaoSheroz KhanGuangcai FengShervin HashemiGuangfu WuShervin MinaeeGuanghao SunShervin R. ArashlooGuanghui WenSheryl BrahnamGuangli LiShiba KuanarGuanglong DuShichang XuanGuanglou ZhengShichao DingGuangming DongShichao JinGuangming XieShichao KanGuangming XiongShichao NiuGuangming ZhangShidan WangGuangpen ZhangShideh Kabiri AmeriGuangrong ShenShieh-Kung HuangGuangxin HeShien RiGuangxing WangShien-Fong LinGuangxu ChengShigang LiGuangyan HuangShigeru ToyamaGuangyu QiuShih-Chieh ChenGuangyuan (Clark) LiShih-Chun LinGuanjun LiuShih-Hau FangGuanqiu QiShih-Jeh WuGuanyi MaShih-Kun LiuGuanyi WangShihong XiaGuanyu MaShi-Huang ChenGueesang LeeShih-Wei SunGuennadi SaikoShih-Wei TanGuglielmo ColaShih-Yu LiGui LiuShijie FengGui Yun TianShijing SunGuido BelforteShi-Jinn HorngGuido BorghiShijun LiuGuido EhrmannShikuan YangGuido GentiliShilong SunGuido La RosaShimaa A. Abdel Hakeem AbdelhakeemGuido LinkShiming HeGuido MaielloShimshon BelkinGuido MarsegliaShin HurGuido PanzarasaShin Jun ParkGuifang DongShinae AhnGuifen WangShinfeng LinGuigen LiuShinichi SatoGuijin WangShinichi SobueGuilherme B. LucasShinichi YamagiwaGuilherme DeSouzaShinichiro OkazakiGuilherme Lucio Abelha MotaShinichiro OmachiGuilherme Luiz MoritzShinji KajimotoGuillaume AndrieuxShinkyu Shinkyu ParkGuillaume DardenneShinnazar SeytnazarovGuillaume DebaeneShinpei OgawaGuillaume DucournauShintaro IzumiGuillaume J. BilodeauShintaro SukegawaGuillaume LozenguezShinya IkenoGuillaume PuelShiqiao QinGuillaume RicciardiShirin MalihiGuillaume SandouShirsendu SikdarGuillemette RibayShisheng LinGuillermo Álvarez-NarciandiShishir PriyadarshiGuillermo Cámara-ChávezShisong CaoGuillermo De ArcasShitala PrasadGuillermo Del Campo JimenezShitao ZhuGuillermo GallegoShiuh-Chuan HerGuillermo Garcia-ToralesShiv K. SharmaGuillermo Hernandez-RamirezShivaram ArunachalamGuillermo Morales LunaShivesh KumarGuillermo Moreno-AlcantarShi-Wei LoGuillermo R. IglesiasShiwei MoGuillermo Rodriguez-NavasShiwen MaoGuillermo Ronquillo LomelíShixi YangGuillermo RusShiyang LiGuillermo Salceda-DelgadoShiyang TangGuillermo Valencia-PalomoShiyi TianGuiping WuShiyu XieGuisong YangShi-yuan HanGuiwei LiShiyuan WangGuixiong LiuShizhi ChenGuiyang XinShlomo GreenbergGulam RabbaniShmuel SpringerGun LiShoaib AzamGuna HewaShogo OkamotoGungun LinShohei NaitoGunnar SchuetzShoji KakioGunnstein FrøsethShotaro YoshidaGunter NotniShou-Hu XuanGunther NotniShoujun XuGünther RetscherShoukun WangGunzung KimShoushui WeiGuo JiangShow-Shiow TzengGuo Liang GohShriram S. RangarajanGuo LiuShu GongGuo YahuiShu LeiGuoan YangShu Ting GohGuo-Cheng HanShuai FanGuodong YeShuai GaoGuofa LiShuai LiGuogang ZhangShuai LiuGuohao DaiShuai WangGuohua WeiShuai ZhangGuokun LiuShuai ZhaoGuoliang LuShuaiqi LiuGuoliang ShangShuaiyong LiGuolin SunShuanfeng ZhaoGuolong ZhengShuang DuGuoman HuangShuang LiGuomei ZhangShuang LiuGuoniu ZhuShuang MeiGuoqi QianShuang SongGuoqiang LiShuang ZhangGuoqing LinShuang-Jian JiaoGuoqing ZhaoShuangquan WangGuosheng XuShuangshuang FanGuo-Shiang LinShuangyi WangGuoshuai ZhaoShuangzhuang GuoGuoxiu HeShu-Chuan ChuGuoxuan LianShu-Cing WangGuozhao JiShufeng LiGurjit Singh WaliaShuhua FangGururaj Kudur JayaprakashShuicai WuGustavious Paul WilliamsShuihua JiangGustavo Arroyo-FigueroaShuiliang FangGustavo Chica PedrazaShuilong ShenGustavo GilShuisen ChenGustavo MesiasShuja AnsariGuy Ayissi EyebeShu-Kai S. FanGuy BurroughesShukui LiuGuy MarlairShuming JiaoGuy MélardShu-Ming TsengGuyue ZhangShuming YangGuzel ZiyatdinovaShumit SahaGwan-Hui LeeShun HuGyanendra P. JoshiShun WangGyani Shankar SharmaShunbo LiGyeongsik YangShun-Hsi HsuGyörgy EignerShun-Hsyung ChangGyörgy MolnárShunjun WeiGyörgy NádasyShunki ItaderaGyu Myoung LeeShunmei MengGyu Weon HwangShunping ZhangGyula MesterShun-qing RenH. Peter WhiteShunsuke NagahamaHa Duong NgoShunsuke ShigakiHa Manh DoShunsuke TomitaHa Sul KimShunsuke YoshimotoHaakon Elizabeth LindstadShu-Nung YaoHab. Izabela RejerShunyun ChenHabib RahmanShuo GaoHaci BaykaraShuo LiHada Fongha IeongShuo ShiHadi DelavariShuo WangHadi MoeinShuo YangHadia AwadShuohong WangHady HabibShuo-Tsung ChenHaejoon JungShupeng LiuHaewon ByeonShuping WuHafiz Muhammad AliShurong DongHafiz Suliman MunawarShurui FanHafiz Tayyab RaufShusheng BiHafsa Korri-YoussoufiShushi NambaHai Bin ChenShuting ChenHai DongShuting YangHai LiuShuxiao LiHai Ning LiangShu-Yi TuHaibin WuShuyin XiaHaibo FengShuyuan XiaoHaibo GongShuzeng ZhangHaibo ZhouShu-Zhi LiuHaichao HongShyan-Ming YuanHaider AliShyi-Chy ChengHaider Al-KhateebSibel CetinelHaidi AbdullahSiddharth SaksenaHaidi IbrahimSiddhartha KhareHaidong ShaoSiddheswar MaikapHaidy ElghamrawySidney Bruce ShikiHaifeng ZhangSidney GauthreauxHaigang SuiSidorov Nikolay V.Haihang YeSierra YoungHaihua ChenSifat MuinHaijie TongSigal PortnoyHaijun PengSigitas TamulevičiusHaili SunSigurd EskelandHailiang LiuSihyung LeeHailing XiongSikai ChenHailong HuangSikha BaguiHailong YinSikora WojciechHaim AbramovichSiliang LuHaimei GongSilke Anna Theresa WeberHaiming JinSilvana Gómez-MeireHai-Pang ChiangSilvano DonatiHaipeng DaiSilvano DragonieriHaiping HuangSilvano ValandroHai-Po ChanSilvestro RoattaHaiqiang NiuSilvia ArazuriHaitan XuSilvia BiasottiHaitao YuSilvia CeccacciHaitao ZhengSilvia KrugHaitham Abu GhazalehSilvia Liberata UlloHaixin SunSilvia Martínez SatorresHaixing LiuSilvia Mastrolembo VenturaHaiyan GuanSilvia MiguelHaiyang GaoSilvia MirriHaiyang MaoSilvia MuraHaiyang ZouSilvia OrtínHaiying MaSilvia PiccioliniHaiyong LuoSilvia San Román MataHajime KamebuchiSilvia SangiovanniHajin ChoiSilvia UlloHa-Jin YuSilvie BernatováHak-Seon KimSilvina FaisHaleh NazemiSilvio Assis De Oliveira-JuniorHalil Ersin SökenSilvio CocuzzaHalina TarasiukSilvio LorenzettiHamdi ZurqaniSilvio NoceraHamed HabibiSilvio SabatiniHamed MalekmohammadiSilvio SampaioHamed MohammadbagherpoorSilvio SciortinoHamed YazdanpanahSilvio SimaniHamid R. SayarshadSilviu ButnariuHamid Reza AhmadiSilviu NastacHamid Reza KarimiSima NoghanianHamid Reza MaratebSimas RackauskasHamida HallilSimeone DussoniHamidreza FahhamSimon AnnaheimHammam AlshazlySimon AvrillonHamza AlkhatibSimon GayHamza BaniataSimon HuHamza BoukabacheSimon J. BleikerHamza ShakeelSimon KrašnaHan DingSimon PezzuttoHan Eol LeeSimon SchweidlerHan GaoSimon TaylorHan HuSimon ThibaultHan Lim LeeSimon TomažičHan OuyangSimona BiancoHan Sang IkSimona Di MeoHan ZhangSimona KubíkováHana TomáškováSimona MiclausHanako AsaiSimona MoldovanuHanan HindySimona PetrakievaHanatsu NaganoSimona RamanauskaitėHanbai ParkSimona RiureanHanbin MaSimona TeccoHanbit JinSimona Viorica HalungaHanfei MeiSimonas RamanaviciusHang DingSimone ArenaHang GuoSimone BaldiHang Nga MaiSimone BattistiniHang SuSimone BorriHangil JoeSimone CosoliHanho WangSimone Dal ZilioHanhoon ParkSimone Di DomenicoHanhui LiSimone FerrariHani J. KbashiSimone FioriHani RamadhanSimone GenovesiHanif AdeelaSimone LolliHanjiang LuoSimone MantelliniHan-Joon KimSimone MentastiHanjung SongSimone MorosiHank WhiteSimone SantoroHan-Kyu ChoiSimone SchuerleHann Woei HoSimone SterniniHanna BandarenkaSimone ZanottoHanna JärveläinenSimonetta CaponeHannes SchnieppSina DavidHanno HildmannSinan SousanHannu NieminenSin-Chi KuokHans HartnagelSitakanta SatapathyHans Martin SauerSiti Hajar OthmanHans Martin SchwabSiting LiuHans Peter LangSiting XiongHans-Joachim KrauseSiu Shing ManHans-Peter BischofSiya HuangHan-Wei ChangSiyan MaHany F. AtlamSkanda VivekHao ChengSkibicki JacekHao ChiSky LeeHao JiangSlaven AbdovićHao LiSlavenko StojadinovicHao LuSlavica Malinovic-MilicevicHao QuSlavisa TomicHao TianSlavko RupcicHao WangSlavomír LabantHao XiongSlawomir BlonskiHao YangSławomir CięszczykHao ZhangSlawomir GonkowskiHaocai HuangSławomir GryśHaochen HuaSławomir HausmanHaochong HuangSlawomir JudekHaofei YuSlawomir KlosHaojun AiSławomir KocońHaoqi SunSlawomir SujeckiHaoqing LiSławomir WierzbickiHaoran ChiSlim NaifarHaoran WangSlimane LarabiHaoran WeiSlobodan BabicHaradhan KolyaSlobodan RadojevicHarald MoserS.M. Sohel MahmudHarald RennhoferSmolovik MikhailHarby MostafaSmriti BhattHaret RosuSnježana Firšt RogaleHari PalaniSobhan RoshaniHariharan LalgudiSobhan SoleymaniHarikumar RajaguruSocrates BasbasHarini NarayananSofi Arshavsky-GrahamHarish ChanderSofia Balula DiasHarish DevarajSofia ScatagliniHarish GargSofiane BoucennaHarleen KaurSoha AhmadiHarold I. ZeligerSoheil KeshmiriHaroldo T. HattoriSoheil Sadeghi EshkevariHaroon AmanSohel RanaHaroon MahmoodSohrab FerdowsiHaroon MalikSoi Hoi LamHarri HakulaSokratis TsantisHarsha KalutarageSoldado Cabezuelo Ana BelénHarshad MishraSolmaz S. KiaHarshkumar PatelSomasekhar R. ChinnadayyalaHartmut F. WitteSomayeh B. ShafieiHartmut HinzSomayyeh AsgariHarumi HottaSomdip DeyHaruo KobayashiSomeshwar RoyHasan AbbasSon Thanh NguyenHasan AbdulrahmanSon Trinh-VanHasan Onur KelesSong FengHasan S. MirSong Joo LeeHashim HabiballaSong LiHasim AltanSong ZhouHasmat MalikSonghao ShangHassan AlgadiSonglin ZhangHassan AliSongling HuangHassan ChizariSongrui WeiHassan El AlamiSongtao XueHassan ElahiSonia GouveiaHassan Haes AlhelouSonia GregoHassan Karimi-malehSonia Helena Contreras-OrtizHassan NosratiSonia Ortiz-PeregrinaHassan RabahSónia SimõesHassane HotaitSonja GamsjaegerHayder Al-HraishawiSonja GeorgievskaHayun ChungSoo Young ShinHazi Mohammad AzamathullaSoochang ParkHe FangSook-Ling ChuaHector BecerraSook-Yee ChongHéctor Cardona-ReyesSooman LimHector D. MenendezSoon Hock NgHector Eduardo RomanSoonjae AhnHector ErivesSoon-Jae KweonHéctor Espinós-MoratóSoonmin SeoHector García-MiquelSoonseok SongHéctor Manuel Pérez-MeanaSoonwook KwonHector MontesSoon-Yong ParkHéctor Moreno-RamónSoo-Whan ChungHector SolarSoo-Whang BaekHee Chan KimSooYoung KwakHee Lee-NiiniojaSophia Diana RozarioHeechul JungSorana BolboacaHee-Deok YangSorin BarabasHee-Jun KangSorin CiortanHeejun RohSorin HinteaHeejung KimSorin VlaseHeena RathoreSorinel CăpușneanuHeeryon ChoSossena WoodHeeSeok KimSotirios DiamantasHeesuk SonSotirios KontogiannisHeewon JungSotiris AvgoustiHeeyoung LeeSotiris E. NikoletseasHeghes BogdanSotiris KotsiantisHei LawSoumya MannaHei WongSoundaram Jeevarathinam AnanthakrishnanHeike EbendorffSourabh SolankiHeike HerperSourangsu BanerjiHeilig BalázsSourav RejHeinrich HussmannSourav Sen GuptaHeinrich RuserSouvik GhoshHeinz PerneggerSowon HahnHejun WuSoyeon LeeHelbert Eduardo EspitiaSo-Yeon YoonHélder FonsecaSpandan RoyHelen C. LeligouSpartacus GomarizHelen HarmanSpiridoula V. MargaritiHelena Cristina VasconcelosSpiros A. KostopoulosHelena Fernandez VaroSpiros BezantakosHelena NavasSpiros D. DafnisHelena Rifà-PousSpiros GeorgakopoulosHelena Svobodová KosnáčováSpyridon BlionasHélène De CannièreSpyridon K. ChronopoulosHélène Niculita-HirzelSpyridon KintziosHelene PilletSpyridon VosinakisHelin YangSpyros HirdarisHelinando Pequeno De OliveiraSrećko KrileHelko BorsdorfSreejesh SreedharanHelmut Karl LacknerSreekar Babu MarpuHelmut KlöckerSrete NikolovskiHelon Vicente Hultmann AyalaSridhar ArjunanHemal NaikSrinivas HanasogeHemant PanditSrinivas KotaHemi QuSri-Rajaesekhar KothapalliHeming WeiStamatios AmanatiadisHemraj M YadavStamatis KarlosHendrik Jan Van Der WoerdStan MariusHendrix DemersStanislao GraziosoHeng WangStanislav A. EvlashinHengcai ZhangStanislav HarizanovHengchang BiStanislav KurajicaHengfeng GuStanislav PopelkaHeng-Tung HsuStanislav TrdanHenri AchtenStanislav ZvánovecHenriette HeinrichStanislavas DadeloHenrik KoblauchStanisław BaciorHenrik ZsiborácsStanisław DeniziakHenrique E. M. PeresStanisław DuerHenrique Ferreira Da Silva PauloStanislaw FlagaHenrique MamedeStanisław HożyńHenrique SilvaStanisław SaganowskiHenry GriffithStanisław StopińskiHenryk KrawczykStanisław SzombaraHeoncheol LeeStanley Udochukwu OfoegbuHerbert EnserStavros DimopoulosHerbert KimuraStavros KarkanisHerbert Michael HeiseStavros KoliosHerman C. MyburghStavros KoulouridisHerman L. OfferhausStavros LazarouHerman Van WerkhovenStavros SouravlasHermann GillyStavros VologiannidisHermano VeltenStavroula KordellaHermes GibertiStavroula NtoaHernan GonzalezStavroulakis GeorgiosHernán Morales-NavarreteStef VandermeerenHernando P. BacosaŞtefan BodiHerng-hua ChangStefan DomekHerve AubertStefan FischerHesam BabahosseiniStefan ForstnerHesham MH ZakalyStefan Gheorghe PentiucHeshan LiuStefan GumholdHe-sheg WangStefan K. EhrlichHessam GolmohamadiStefan KnauthHeungjoo ShinStefan KolevHezhen LouStefan KüchemannHiam KhouryStefan LüdtkeHichem MrabetStefan LüthHideaki MurayamaStefan MihaicutaHideaki NozatoStefan NaydenovHideaki UchiyamaStefan PanicHidekazu TakanoStefan PratschnerHideki Hideki TomitaStefan SauermannHideki KomagataStefan SchneiderHideki NaitoStefan TabacuHiep Duc NguyenStefan ThomasHiginio Rubio AlonsoStefan WallnerHikaru AonoStefan WeberHimanshu AgrawalStefan ZauscherHimer Avila-GeorgeStefania CostantiniHinrich GrotheStefania FedericiHira FatimaStefania MarsiliHiram CalvoStefania MontaniHiroaki HayashiStefania RomeoHiroaki KikuchiStefania TamburriniHiroaki KuzeStefania VitiHiroaki MorinoStefano AmatoriHiroaki NishikawaStefano BettatiHiroaki SatohStefano BrusaporciHirofumi NonakaStefano CaputoHirokatsu KataokaStefano CarrinoHirokazu MadokoroStefano Del SordoHiroki AshibaStefano DomekHiroki IshizukaStefano EliaHiroki ManoStefano FornasaroHiromasa ShimizuStefano FortiHirosato MonobeStéfano Frizzo StefenonHiroshi ChureiStefano GuarinoHiroshi DozonoStefano LaiHiroshi HigashiStefano LauretiHiroshi KagemotoStefano LenciHiroshi MatsuoStefano MangioneHiroshi OzawaStefano MarianiHiroshi TanakaStefano MauroHiroyoshi OhtsuStefano PalermiHiroyuki OkamotoStefano ParrinoHiroyuki SatoStefano PastorelliHisham ArafatStefano PerilliHla Myo TunStefano PinardiHo Won JangStefano PrimatestaHo Won LeeStefano QuerHoa Tran-DangStefano RinaldiHoang Long NguyenStefano RossiHoang NguyenStefano SalataHoang-Long CaoStefano SalvatoriHocheon YooStefano SapienzaHocine CherifiStefano SelleriHodjat PendarStefano SerianiHoerner StefanStefano SfarraHoi-Shun LuiStefano StassiHojong ChoiStefano TenninaHojoong KimStefano TomasinHo-Jun SukStefano ZampolliHolger BabinskyStefano ZuccaHolger HillSteffen FinckHolger MauneSteffen WendzelHong BaoSteffen ZeuchHong ChiŠtefica MrveljHong DingStefka AtanassovaHong LiStela-Maria PruneanuHong LinStelian Alexandru BorzHong LiuStelian LupuHong MenStelios IoannouHong PanStelios K. GeorgantzinosHong Ren WuStelios ZimerasHong SongStella HrehováHong TangSten HankeHong WangStepan PapacekHong XieStephan JonasHong ZengStephan KallweitHong ZhangStephan M. JonasHong ZhaoStephan WarnatHongbin LinStephane BarlandHongbin PuStephane GuinardHongbin SunStéphane PerreyHongbin YuStephane RioualHongbing DengStéphane RouxHongbing JiStephanie R. RogersHongbo GaoStephanie ValentinHongbo GuStephen BruderHongbo WangStephen CainHongbo ZhangStephen CanfieldHongchao FanStephen CzarnuchHongchul SohnStephen G. WilsonHongde QinStephen MihailovHongen LiaoStephen MonkHongfeng YuStephen MorganHonggui LiStephen PowersHong-Hao GaoStephen SchultzHongjie JiangStephen U. EgarievweHongju MaoStephen WeisHong-Jun XiangSteve BeebyHongkook KimSteve ChanHonglang LiSteve GlassHongli HuSteve LingHongliang LüSteve MannHongliang RenSteve O’HernHongliang ZhouSteve RothbergHongmin WuSteve SemancikHongming XuSteven ChattertonHongpeng WuSteven HobbsHongpeng ZhangSteven J. JohnstonHongseok AnSteven J. SteinbergHong-Seok ParkSteven LatréHongsheng LiSteven Le CamHongsun GuoSteven LevyHongtak LeeSteven M. PetersonHongtian ChenSteven P. BennettHongwei QuSteven Ross MurrayHongwei WangSteven Van WindenHongwu WangStoyan SlavovHongxin HuangStrahinja DosenHongyin ShiStuart McErlain-NaylorHongyu HuStuart PerryHongyu YuStyliani VerykokouHongyu ZhaoStylianos D. AssimonisHongYu ZhengStylianos MystakidisHongyuan HuoStylianos SiskosHongyue SunSu Man NamHongyun LiuSu ZhangHong-Zen YehSuan LeeHongzhou YangSuat MercanHoon OhSubhamoy MandalHoon SohnSubhash SinghHorácio OliveiraSubhashis HazarikaHoracio PaggiSubhasis ThakurHoratiu StefanieSubhayan MukherjeeHorn-jiunn SheenSubhransu SahooHosam Al-SamarraieSubodh DhakalHosameldin AhmedSubrata ChakrabortyHosang AhnSuchinder K. SharmaHoseon LeeSudarsana Reddy KadiriHosik SeokSudheer Kumar BattulaHoson LamSudipta BanerjeeHossain Md. BellalSuhas SomnathHossam A. GabbarSuhua TangHossam SelimSuh-Yeon DongHossein BagheriSuhyun ParkHossein FotouhiSuixin LiuHossein GhaemiSujat SenHossein HassaniSujita NarayanHossein HondoriSujoy GhoshHossein MardaniSujoy Ghosh HajraHossein SahourSuk Jin LeeHossein Tabatabaei-JafariSukanta Kumer ShillHossein TaheriSukho LeeHosseon RabbaniSuku NairHou-bin LiSulaymon L. EshkabilovHoubing SongSüleyman Fatih ÖzmenHoupu LiSultan MahmudHoussam SalmaneSultan ShoaibHoward MatisSumana BiswasHowuk KimSumin KimHo-Yong AhnSun K. HongHriday BavleSun Kwon KimHridesh Kumar MishraSun Woog KimHristos AnastassiuSunan HuangHrvoje GlavasSuncheol KwonHrvoje JakovacSunday EkpoHrvoje KalinićSung Min ParkHrvoje KarninčićSung YangHrvoje KutnjakSunggu LeeHsi Lien HsiaoSunghae JunHsiang-Chen WangSung-Hak LeeHsiang-Chieh ChenSung-Ho BaeHsiang-Chieh LeeSungho JeonHsiao-Chuan LiuSungho KimHsiao-Chun WuSungho LeeHsiao-Ping TsaiSunghyup Sean HyunHsiao-Ting TsengSungjun KimHsiao-Tung ChangSungkyu Shaun ParkHsieh Bao-YuSung-Liang ChenHsien-Pin HsuSung-Man KimHsien-Tsai WuSung-Min HongHsien-Wei YehSung-Phil KimHsi-Hsir ChouSungtae ShinHsin HsiuSungtek KahngHsin-Han ChiangSung-won ChoHsin-Jang ShiehSungwook ChoHsin-Ta ChiaoSungwook MhinHsinwen TingSungyong ChoiHsin-Yi Kathy ChengSung-Yong SonHsin-yi WenSung-Yun ParkHsiu-Ming WuSunil ChakrapaniHsuan-Ling KaoSun-Joo JangHsuan-Shih LeeSunmean KimHsueh-chun LinSunny RajHsueh-Hsien ChangSunwook KimHsueh-wen ChowSunwoong ChoiHsueh-Wen TsengSuoda ChuHsu-Heng YenSuparerk JanjarasjittHu DingSupriyo BandyopadhyayHu HeSupriyo RayHu SunSuraparb KeawsawasvongHu XiongSuren KumarHua LiSurender Reddy SalkutiHua ShiSuresh NeethirajanHua SunSuryadipta MajumdarHua XuSusan Ann O’ShaughnessyHua ZouSusan M. WernimontHuachun WuSusan ZhouHuachun ZhouSusana CatarinoHuafei DuSusana Dias AmaralHuafeng LiuSusana FernándezHuai-Kuei WuSusana Hornillo MelladoHuaiyu YangSusana PalmaHuajun SongSusana SilvaHuakun HuangSusanna KaiserHualong XieSusanna SpinsanteHuan HeSusanna SummaHuan LiuSusanne M. Van Der VeenHuan NiSushanta MitraHuan PangSushil Kumar SinghHuan WangSushruta SurappaHuan ZhaoSusil BaralHuang LiSusmit ShannigrahiHuangke ChenSusumu IshiharaHuan-Hsuan HsuSusumu OkumuraHuanyu ChengSutanu BhowmickHuanyu (Larry) ChengSutripto MajumderHua-Pin ChenSuyun HamHuaqing CuiSuzana UranHuaqing WangSuzanne E. BlattHuawei JiangSuzy NgomoHuawei LiangSvatopluk MatulaHuaxiang WangSven G. BilénHuaxing JiangSven MayerHubin ZhaoSvetla StoilovaHucheng SunSvetlana BoudkoHuei-Yung LinSvetlana GirsHuey Hoon HngSvetlana KhoninaHuey-Ming TzengSvetlana MarkovaHugh J. ByrneSvetlana N. KhoninaHugh LeeSvetlana Von GratowskiHugo A. LoaicigaSvilen Petrov SabchevskiHugo Almeida FerreiraSvitlana KopylHugo AndréSwaminath VenkateswaranHugo Entradas SilvaSwati J. PatilHugo F. MartinsSyed A. BukhariHugo F. Posada-QuinteroSyed Afa Ali ShahHugo Fernando Posada-QuinteroSyed Agha Hassnain MohsanHugo FerreiraSyed Ali Kalangala Sikkanther BatchaHugo G. EspinosaSyed Aziz ShahHugo J. Avila-ParedesSyed Basit Ali BukhariHugo José Nogueira Pedroza Dias MelloSyed Furqan QadriHugo Rojas-ChávezSyed Khairul BasharHugo SanerSyed Muhammad AnwarHugo SarmentoSyed Muhammad AsadHugues PretrelSyed Muhammad UsamaHuh Tae MyungSyed Muzahir AbbasHui ChenSyed Tahir Hussain RizviHui HuangSyed Tariq ShahHui JiangSyed Thouheed AhmedHui LiSyed Turab RazaHui MaSyeda Adila AfghanHui SunSyeda Fizzah JilaniHui WangSyed-Abdul ShabbirHui YangSylvain BertrandHui ZhangSylvain FeruglioHui ZhengSylvain GirardHuie ZhuSylvain GrenierHuifang LiSylvain LeclerHui-Hsin HsiaoSylvain MagneHuijia LiSylvain MezilHuiling TaiSylvie ChambonHuilong ZhangSylvio Barbon JuniorHuimin LiuSylwester BorowskiHuimin LuSylwia BalutaHuimin ShenSylwia SzczęśniakHuimin ZhangSylwia Szporak-WasilewskaHuixiang WuSylwia Werbińska-WojciechowskaHuixuan FuSynh Viet-Uyen HaHumberto C. GodinezSytze De BruinHumberto Lobato-MoralesSyuichi ItahashiHumberto Martínez-BarberáSzczepan PaszkielHumberto SossaSzilard CsizmadiaHung CaoSzilvia NagyHung NguyenSzücs VeronikaHung Soo KimSzymon ChorazyHung Tuan NguyenSzymon CyfertHung-Chi ChuSzymon ŁośHung-Huan LiuSzymon MalinowskiHung-Jr ShiuSzymon OgonowskiHung-Shing ChenSzymon SiecińskiHung-Vu TranSzymon WójtewiczHung-Yin LinTad BrunyeHung-Yu ChienTad GonsalvesHunter BachmanTadamasa SawadaHuong Thu NguyenTadashi ItoHuseyin KusetogullariTadeusz GłowackiHüseyin PolatTadeusz MikolajczykHusnain SheraziTadeusz PisarkiewiczHussam HusseinTadeusz SondejHuxuan WangTadeusz StepinskiHwajeong SeoTae Joon JunHwang Seung-HoonTae Keun YooHwang YiTae-Eung SungHwang-Cherng ChowTaeho JungHyejeong RyuTaehoon KimHyeon Seok AnTaejoon KimHyeong-U KimTahir KhurshaidHyeon-June KimTahmid LatifHyogon KimTahseen A. AlwattarHyojung HyunTai FeiHyosang YoonTaihong LiuHyoseok YoonTaimur HassanHyoungmin SoTaiping ZengHyowon BanTairan LiuHyoyoung JeongTaisong PanHyuck Jin LeeTaiwen TangHyuk-Jae RohTaiyong LiHyun Cheol KimTakafumi AndoHyun Ho LeeTakahiro ArakawaHyun KwonTakahiro KannoHyunchae ChunTakahiro KuchimaruHyunchul KangTakahiro KunimineHyunchul ShinTakamichi MiyataHyun-Deok KimTakashi ChibaHyung D. BaeTakashi IidaHyung Seok KimTakashi IsezakiHyungchul YoonTakashi OkayasuHyungjong KimTakashiro AkitsuHyung-Joon SeoTakehisa HanawaHyung-Tae KimTakeshi FujinoHyun-Ho ChoiTakeshi Go TsuruHyunjoo Jenny LeeTakeshi HashishinHyunjung LeeTakeshi KamiyaHyunseok SeoTakeshi YanagidaHyunsoo LeeTakeyasu SakaiHyunwoo HwangboTakuma AkimotoIacopo TamellinTakumi AsakuraIain CollingsTakumi OkabeIan A. GroutTakuya KiyokawaIan ClelandTales Cleber PimentaIan CurthoysTalles Marcelo G De A BarbosaIan Daniel LerouxTam NguyenIan DavisTamara BonaciIan McAndrewTamara RadivilovaIan MossTamás BécsiIan WilliamsTamas HaideggerIaroslav GnilitskyiTamas OroszIatrellis OmirosTamás PardyIbrahim AbdulhalimTamás RuppertIbrahim Furkan InceTamas VargaIbrahim YitmenTamio IkehashiIbukun Gabriel AwolusiTan Jen HongIchiro FujitaTan YigitcanlarIchiro YamadaTanesh KumarIckjin KwonTania GarcíaIclia Villordo JimenezTaniguchi NaokazuIc-Pyo HongTanja DudenbostelIdiano D’AdamoTanju YildirimIdiano D’AdamoTanmay KulkarniIdilia BatchkovaTanveer AhmadIdongesit EkereteTanweer AlamIdowu Paul OkuwobiTanweer AliIdris AhmedTanya HutterIeva Meidute-KavaliauskieneTanzilur RahmanIeva PlikusieneTao CaoIevgeniia KuzminykhTao ChenIftekhar KhanTao HongIga Hołyńska-IwanTao HuIga RybickaTao HuangIgnacio BravoTao JiangIgnacio Fernández-HernándezTao LeiIgnacio Javier PérezTao LiIgnacio LijarcioTao LiuIgnacio Llamas-GarroTao LuoIgnacio Rodríguez-RodríguezTao ShaohuaIgnacio RoigTao ShenIgnas BudvytisTao WangIgnas GrigelionisTao WuIgnasi CorbellaTao YangIgor A. KonyakhinTao YinIgor AleinovTao YueIgor BufetovTao ZhangIgor BuzalewiczTao ZhouIgor BychkovTao-Hsing ChenIgor ChunchuzovTara Julia HamiltonIgor FilanovskyȚarălungă Dragoș DanielIgor FürstnerTarek BerghoutIgor GruicTarek DjerafiIgor KorobiichukTarek ElfoulyIgor LobodaTarek GaberIgor OgashawaraTarek YoussefIgor PusnikTareq AssafIgor RužićTareq KhanIgor SaenkoTarig AliIgor SavinTariq AbdullahIgor TomicicTariq AliIgor VujovićTaro UedaIhr Jörg SchäferTaron MakaryanIhsan AliTasadduq ImamIker Pastor-LópezTasneem DarwishIk-Hyun YounTatevik ChalyanIkjune YoonTatiana AtanasovaIkram Ud DinTatiana JaworskaIl Song HanTatiana KováčikováIla MalteseTatiana KuchmenkoIlaria BortoneTatiana LatychevskaiaIlaria CampioniTatiana M. PinhoIlaria De MunariTatjana GricIlaria FerrandoTatsiana MikulchykIlaria MiletiTatsuaki TagamiIldiko MankovaTatsuo YoshinobuIldiko SzantoTatsuro GodaIleana BogdanTatsuya IwataIleana HamburgTatu KumpulainenIleana Nicoleta PopescuTauhidul AlamIleana PirovanoTauseef JamalIl-Gu LeeTawfik KhattabIlia N. IvanovTawfik NajehIlia SafonovTawhidul Islam KhanIlias MavroidisTaylor ChomiakIlias N. LymperopoulosTe HanIlias PolitisTea Duplančić LederIlias TheodorakopoulosTecla BonciIlias TravlosTeddy CraciunescuIlie Valentin MihaiTee Hui TeoI-Lin TsaiTeemu RäsänenIlja CroijmansTeen-Hang MeenIlke Coskun BenlidayiTeerawat KumraiIlkka NissinenTejasvi ParupudiIlona Jacyna-GołdaTe-Jen SuIlona PawełoszekTelmo FernandesIl-Seuk KohTemple GrandinIlshat KhasanshinTeng LiIltai (Isaac) KimTengfei ChangIl-Woo NamTengfei LongIlya N. PavlovTeodor B. IlievIlya TurchinTeodor TothIlya YakavetsTeodorico CaporasoImad RidaTeodoro GeorgiadisImadeddine AzzouzTeodoro MontanaroIman Abaspur KazerouniTeresa Albero-AlberoIman Heidarpour ShahrezaeiTeresa HortonIman IzadgoshasbTeresa NogueiraIman Soltani BozchalooiTeresa SantosImena RexhepiTeresa ZawadzkaImmihan Ceren YasaTeresa ZwierkoImran AshrafTerézia PošivákováImran BaigTerje TjeltaImran Khan NiaziTerrence J. MooreImran MemonTessai HayamaImran NiaziTetiana ManykImran TasadduqTetsuo IguchiImre KissTetsuo SasanoImtiaz AhmedTetsuro YanasekoImtiaz MahmudTetsushi IkedaIm-Yeong LeeTetsuya KawanishiIn Soo LeeThabet KacemInacio YanoThad WalkerInayat Ur RahmanThaís De SáInder GuptaThanasis KoutrasInes DelfinoThanasis LoukopoulosInes KožuhThanh Hai LeInes ObradovićThanh Luan PhanInés Sittón-CandanedoThanh NguyenInga Morkvenaite-VilkoncieneThanh Nho DoInge WiekenkampThanh-Canh HuynhIngmar MüllerThanikanti Sudhakar BabuInhee LeeThar BakerIn-ho RaTharmalingam SivarupanInhong SongThejas Gubbi SadashivaIñigo BarandiaranTheo G. KanterIñigo CuiñasTheodor D. PopescuInma Mohino-HerranzTheodore A. CorcovilosInmaculada AparicioTheodore KotsilierisInmaculada Romero GilTheodore ZahariadisInnokentiy KastalskiyTheodoros KarakasidisInseop NaTheodosios SapounidisInsoo KimTheofilos ChrysikosIntisar Ali SajjadTheofilos PapadopoulosInwook HwangTheoklis NikolaidisInyong KwonThiago M. B. F. OliveiraInż. Bartosz OlejnikThiago PoletoIoan BurdaThiago SequinelIoan NicolaescuThibault LaplaceIoan PeteanThibaut RaharijaonaIoan SileaThien D. NguyenIoan Stefan SacalaThien Huynh-TheIoan SusneaThierry BaccinoIoan TudosaThierry Oscar EdohIoan UngureanThilo SandnerIoana Dana AlexaThomas BretterklieberIoana MarcuThomas CharrettIoana OprisThomas CilloniIoanis NikolaidisThomas GittlerIoanna RoussakiThomas Heide ClausenIoannis ChatzigiannakisThomas HeinzeIoannis D. MoscholiosThomas HowardIoannis FaraslisThomas J. SmithIoannis KaramitsosThomas KletschkowskiIoannis KioutsioukisThomas KonradIoannis KosmasThomas LagkasIoannis KostavelisThomas LangoIoannis P. GravasThomas M. ArrudaIoannis PaliokasThomas M. DesernoIoannis SamarasThomas M. KaiserIoannis T. GeorgiouThomas M. KraftIoannis TsoulosThomas OttIoannis VardiambasisThomas PurfürstIoannis VourganasThomas ScottIole VendittiThomas SheppardIon Dan BorciaThomas SteffenIon Emilian RadoiThomas StewartIon GiosanThomas WiemannIon SanduThomas ZeffererIon VornicuThran Van ThucIonel StaretuThyago NepomucenoIonel ZaganTiago Almeida SilvaIonica OncioiuTiago BresolinIonut Cristi NicuTiago Fernandes TavaresIonut Daniel GeoneaTiago NascimentoIonut Emil IacobTiago VarumIonut SchiopuTian JinIordana AstefanoaeiTian LiIosif SifakakisTian QiuIosif SzeidertTian TianIosif-Vasile NemoianuTian XinIpin ChangTian ZhouIppokratis SartzetakisTiancheng LiIren E. KuznetsovaTianfeng GuIren ValovaTian-Fu LeeIrena FrycTianhong DaiIrena Ištoka OtkovićTianhua XuIrena IvaniševićTianlin WangIrena JekovaTianqi YuIrena M. HlaváčováTianshu QuIrena OrovićTianxing LiIrene DaskalopoulouTianxun GongIrene NutiniTianyiyi HeIrene Torres-SanchezTiberiu Stefan LetiaIreneusz DominikTiberiu-Paul NeaguIreneusz KubiakTibor GuzsvineczIrenilza De Alencar NääsTibor HianikIrfan Ahmad KhanTibor KrenickyIrfan KhanTibor PasinszkiIrina B. VendikTibor SzkaliczkiIrina D. YushinaTie ZhangIrina G. PalchikovaTiebiao ZhaoIrina Georgiana MocanuTiejiong LouIrina MocanuTielin ShiIrina NeagaTieling ZhangIrina OntelTien-Dung NguyenIrina RabaevTien-Lung SunIrina SeverinTien-Thong Nguyen DoIrina VoytyukTien-Yin ChouIrina YatskivTie-Qiang LiIrineo L. López-CruzTieshan LiIrineu De Brito JuniorTill NierhausIris KicoTim A. MajchrzakIrmantas KašalynasTim Claudius StratmannIrwin A. QuintelaTim MoltenoIsaac MacwanTim MorrisIsaac TrigueroTim VerbelenIsabel CastroTim W. C. BrownIsabel JesusTimea IgnatIsabel Perez De Vargas SansalvadorTimofey ShevgunovIsacco GualandiTimothy M. YoungIsaías González PérezTimothy OtimIshan JoshiTimothy PattenIshtiaq AhmadTimothy S. De SmetI-Shyan HwangTimothy SandsIslam AshryTimothy VincentIslam Md Rizwanul FattahTina BobićIslam MosaTina McKayIsmail ButunTina Tičinović KurirIsmail FidanTina VrabecIsmini E. PapageorgiouTing LeiIsrael Casado-HernándezTing LiIsrael David Hinostroza SáenzTing YunIsrael Donizeti De SouzaTing ZouIsrael HinostrozaTing-Chia OuIsrael Leyva-MayorgaTingguang MaIsrael Martin-EscalonaTingna ShiIsrael Miguel AndrésTingrui LiuIsrar UllahTingting LangIstván BudaiTingting SuiIstvan ElekTingting YangIstván GrigorszkyTitan PaulIstvan GyongyTitus SanduIstván PálinkóTiziana C. BondIstvan RajtaTiziana SegretoIstván VassányiTiziano ZarraItalo AtzeniTjin ChuanItay BarelToan DinhItay BarneaToan Duc CaoItshak TkachTobias GüntherItthipon JeerapanTobias HabisreutherItzamá López-YáñezTobias KalischItzel NunezTobias MeisenItziar Alonso GonzálezTobias NilssonItziar CabanesTobias SchrippItzik KleinTobias StorchIulian CiocoiuToby J. EllmersIuliia NovoselovaTodd MoonIurii SemenovTodd RichardsIvailo PandievTodor CooklevIvan A. DenisovTodor GanchevIvan A. ParinovTodor TagarevIvan Adriyanov NikolovTohru SasakiIvan AleksiTom FoulshamIvan BobrinetskiyTom LahmerIvan BrakTom van der SandeIvan BratchenkoTomas BaležentisIvan CasellaTomás Gómez Álvarez-ArenasIvan ChavdarovTomás GonzálezIvan Cruz-AcevesTomas HorvathIvan CundrleTomas JandaIvan CviticTomas SochorIvan CvitićTomáš ZálabskýIvan D. CastroTomas ZikmundIvan DerkachTomasz BakonIvan DunđerTomasz BieniekIvan ForenbacherTomasz BlicharskiIvan GiannoccaroTomasz CholewaIvan GrgićTomasz CieplakIvan GruberTomasz CudejkoIván Herrera-PecoTomasz DysarzIván Herrero-DuráTomasz GóreckiIvan IudiceTomasz GrzesIvan Ivanovich StoikovTomasz HachajIvan IzoninTomasz HalskiIvan KotuliakTomasz KapuscinskiIvan Lim Chen NingTomasz KikIvan LipuzhinTomasz KisielewiczIvan LorencinTomasz KlekielIvan MarićTomasz KlodaIvan MezeiTomasz KryjakIvan Miguel PiresTomasz KulczykIvan Miguel Serrano PiresTomasz LipeckiIvan MiskulinTomasz MarciniakIvan Oropeza-PerezTomasz MościckiIvan PetruninTomasz NiedobaIvan PetryshynetsTomasz Norbert KołtunowiczIvan PiresTomasz OrczykIvan PlaščakTomasz OsuchIvan ProchazkaTomasz OszakoIvan PuchadesTomasz PanderIvan PuenteTomasz RogalskiIvan ShorubalkoTomasz RokickiIvan Simões GasparTomasz RymarczykIvan TomasicTomasz SipkoIvan VaccariTomasz StareckiIvan VirgalaTomasz StochIvan VujaklijaTomasz SzczegielniakIvan ZajačkoTomasz SzymborskiIvana BarisicTomasz TeleszewskiIvana GadjanskiTomasz TemplinIvana Tlak GajgerTomasz TokarekIvanjko EdouardTomasz WandowskiIvanovitch SilvaTomasz WasilewskiIveta VaskováTomasz WegielIvica StančerićTomasz WiniarskiIvo ButtinoniTomasz WolnyIvo DobrevTomaž AmbrožičIvo DraganovTomaz JavornikIvo IlievTomaz KosarIvo StancicTomaž PodobnikarIvor NissenTomislav BašićIwaki AkiyamaTomislav IvanjkoIwona GrabowskaTomislav KeserIwona GrobelnaTomislav KosIwona KochańskaTomislav MaticIwona KomorskaTomislav MedicIwona PaprockaTomislav PetkovićIzaak NevelnTommaso BacciIzabela Barszczewska-RybarekTommaso ErcoliIzabela GołębiowskaTommaso LomonacoIzabela KarszniaTommaso PecorellaIzabela SzarmachTomoaki KunugiIzabella LeckaTomohiro HayashiIzadyar TamadonTomohiro MashitaIzidor MlakarTomohisa SekiJ. A. De Jesús Osuna-CoutiñoTomohito SekineJ. Apolinar Muñoz RodriguezTomokazu HattoriJ. Ernesto SolanesTomotsumi FujisawaJ. Gabriel Ortega-MendozaTomoya TakadaJ. Gaxiola-CamachoTonći ModrićJ Randall MoormanTongwen LiJaakko HakulinenTong-Xing ZhengJaan OjarandTonino TrainiJaana ParviainenTony LindebergJaap VelthuisTony LuczakJabar H. YousifTony WaterhouseJabed ChowdhuryTony ZhangJacek CabanToomas RangJacek DawidowiczToomas VaimannJacek GebickiTorbjørn SkramstadJacek GórkaToru KuriharaJacek KesikToru WadaJacek Łukasz Wilk-JakubowskiToshiaki HattoriJacek MarciniakToshiaki OmoriJacek MarczewskiToshiaki TsujiJacek MatysToshihiko YoshinoJacek MisiurewiczToshihiro ShimadaJacek NowakowskiToshikazu ShinbaJacek OskarbskiToshiki KanamotoJacek PietraszekToshiya ArakawaJacek PolechońskiTouqeer AhmadJacek StepienTracy Anne HammondJacek TomkówTraianos V. YioultsisJacek WojtanowskiTrent BiggsJacek WojtasTrevor BensonJacek WoźniakTri Dev AcharyaJacek WytrębowiczTribikram KunduJacinto Santamaría-PeñaTriet Nguyen-VanJack BishopTrifol JonJack MerrinTrung Thanh NgoJack WangTruong Xuan DoJackie ReznikTsang-Long PaoJacky LooTsan-Pin WangJacob GrazerTsimpiris AlkiviadisJacob Hinkel-LipskerTsubasa MaruyamaJacob SosnoffTsubasa MinematsuJacopo AguzziTsukuru MinamikiJacopo CataniTsuneo HoriguchiJacopo LamannaTsung-Han TsaiJacopo PavanTsung-Hung LinJacque KellyTsung-Lin ChenJacqueline WalkerTsung-Rong KuoJacques PendersTsung-Yu ChouJader CabralTsutomu TsuboiJae Hee KimTu Le ManhJae Hong ShimTuan Anh NguyenJae Hoon LeeTuan DangJae Joon KimTuan-Minh PhamJae Min LeeTuan-Tu HuynhJae Woong LeeTuanwei XuJae Youn HwangTuba YilmazJaehan KohTudor DeaconescuJae-Han ParkTudor IonescuJaehong KimTudor PaladeJae-Hoon KimTugba KilicJaehwa ParkTuğrul OktayJaehyeok DohTu-Liang LinJaehyuk ChoiTung-Ching SuJaehyun JinTung-Hsun HsiehJae-Hyun KimTural KhudiyevJaehyun ParkTurker DagdelenJaehyung YuTushar Kanti RoyJaejong ParkTutomu MuraseJae-Myung RyuTyler N. TallmanJaepil KoTymoteusz HorbińskiJaerock KwonTzirakis PanagiotisJaesoon ChoiTzu-Chieh ChouJaesun LeeTzu-En LinJaesung ParkTzu-Hsien SangJaewon YangTzung-Shi ChenJaewoo LeeUchenna Daniel AniJaeyong KangUdo FreseJaeyoung LeeUdo NetzelmannJaeyoung ShinUgur AlganciJaeyul LeeUikyum KimJafrancesco DavidUlf JensenJagajjit SahuUlises OrozcoJagannath AryalUlises Páramo-GarcíaJagannathan SarangapaniUlises Pineda RicoJaime Alvarez-QuintanaUllah AminJaime Andres GarciaUlrich BalssJaime Duque DomingoUlrich GuthJaime LloretUlrich SchreiberJaime Martinez-CastilloUlrike KluehJaime MorenoUlrike LechnerJaime Muñoz-ArteagaUma ShahaniJaime OrtegonUmar IqbalJaime Ramis SorianoUmar Zakir Abdul HamidJaime VillateUmberto AmatoJain-Shing LiuUmberto AndrioloJair CervantesUmberto PapaJaka SodnikUmberto RiccardiJakub CiazelaUmer JavedJakub HorákUmer MehmoodJakub KosteleckýUmit OgrasJakub KrejčíUn JiJakub LengiewiczUnal DevrimJakub MarkiewiczUnal Zak SakogluJakub NalepaUpul GunawardanaJakub NedbalUroš KlanšekJakub RydzewskiUrs U. GrafJakub SorockiUrszula MarmolJakub SuchanekUrtė BubnienėJakub SwachaUsman HabibJakub TaradajUsman RashidJakub TrojanUsman Saeed TariqJakub Z. KosickiUte RabeJakub Zbigniew KalitaUtkarsh KohliJakubczyk EwaUwe BrandJa-ling WuUwe HampelJamel AliUwe PliquettJames AveryUwe SerdültJames C. HaUznir UjangJames C. MacnaeVacius JusasJames ConnollyVaclav BuncJames CovingtonVaclav HlavacJames DoutchVaclav KrivanekJames E. Hubbard, Jr.Vaclav KubecekJames GarsideVáclav NavrátilJames LangVaclav PrajzlerJames P. ConnollyVáclav RancJames PennebakerVaclava PioreckaJames RobbinsVadim A. ParfenovJames RodwayVadim AgafonovJamie A. WardVadim GrubovJamil HussainVadim ParfenovJamil Y. KhanVadim S. TynchenkoJamila BoudadenVadim ZhmudJamshaid RahmanVageeswar RajaramJan AwrejcewiczVahab Youssof ZadehJan BaeyensVahid AbolghasemiJan BalzerVahid YaghoubiJan BauerVaibhav ShahJan BroggerVaidas LukoševičiusJan BrusVaidotas MarozasJan CornelisVálber César Cavalcanti RozaJan DizoValderi Reis Quietinho LeithardtJan EggerValdson J. SilvaJan FarlikValentín BarralJan FeslValentin BégelJán GamecValentin BuiculescuJan GrymValentin Cardeñoso-PayoJan GunscheraValentin Gomez-JaureguiJan H SchmidtValentin MateevJan HalámekValentín Osuna-EncisoJan HansmannValentina Alena GirelliJan HavlíkValentina BucciarelliJan HesselbarthValentina De SimoneJan HofsteeValentina FranzoniJan HošekValentina GrippoJán JanošovskýValentina LongoJan KazakValentina YakovlevaJan KelnerValentino MeacciJan KoltermannValentyn KorobiichukJan KomarekValeri I. KovalevJan KonarskiValeria BelluscioJan KoubaValeria LoscriJan KřížValeria RodionovaJan L. CieślińskiValeria RossoJán LabudaValeria SpizzichinoJán LabunValeria-Ersilia OnigaJan LatalValério Antonio Pamplona SalomonJan MandelValerio BiancalanaJan MaresValerio FerroniJan MasajadaValerio ScordamagliaJan MietznerValeriu Manuel IonescuJan MrazekValeriy KrivetskiyJan MućkoValery TsaplevJan MuzikValéry U. ZavorotnyJan NikodemValery V. KalinchukJan NissinenValner BrusamarelloJán PapajVan Dung NguyenJan PennekampVan-An DuongJan PetersenVan-Doan NguyenJán PiteľVanessa CamilleriJan SaligaVangelis OikonomouJan SchmittVangelis SakkalisJan SchürVanlin SathyaJan SikoraVan-Thai TranJan StroblVaradraj Prabhu GurupurJan StudzińskiVarghese KurianJan SuchaniczVarnakavi NareshJan SzatyłowiczVarun Lingaiah KopparthyJan TomastikVarun VyasJan TrmalVasco PereiraJan TuranVasco SoaresJán VachálekVasile A. PopescuJan VilhelmVasile ChisJan VrbaVasile Daniel PavaloaiaJan WajsVasile Gheorghita GaitanJan WarczekVasile MarincaJan WendelVasile PaladeJan WietrzykowskiVasile Paul BresfeleanJana AntalíkováVasile PredaJana HouserVasile SimaJana Kalbacova VejpravováVasile-Gabriel IanaJana StriovaVasileios KalogirouJanaina Antonino PintoVasileios KaryotisJanaka EkanayakeVasileios KomianosJane CourtneyVasileios VlachosJane Jean KiamVasiliki VitaJanet LightVasilios LiordosJanez PeršVasilis ChristofilakisJanez TronteljVasilis K. DertimanisJanghoon YangVasilis KanakoudisJangPyo BaeVasiliy ElaginJanine MatschekVasiliy OsipovJanis BraunfeldsVasko JovanovskiJanis TiemannVassiliki KontargyriJanka SaderovaVassilios C. MoussasJanko SlavičVassilis C. MoulianitisJannes WübbenaVassilis G. KaburlasosJan-Nico ZaechVassilis George AschonitisJános A. OsánVassilis MoulianitisJanos AbonyiVassilis S. KodogiannisJanos BotzheimVedhus HoskereJanos JuhaszVedran MrzljakJános LukácsVedrana Jerkovic StilJan-Peter MullerVedyagin AlekseyJanus Nørtoft JensenVelislava LyubenovaJanusz ĆwiklakVenkata Krishna Karthik TangiralaJanusz DudczykVenkata ManthinaJanusz Marian BȩdkowskiVenkata Prasanth YanambakaJanusz NarkiewiczVenkata Prashant ModekurthyJanusz RajdaVenkata PuliJanusz RusekVenkatakrishnan RengarajanJanusz SmulkoVenkateswaran NarayanaswamyJanusz SzczepanskiVenkateswaran VivekananthanJanusz WaloVera GramignaJaouhar FattahiVeraldo LiesenbergJarek HarezlakVeronica MaierJarilyn M. Hernández JiménezVerónica Montes GarcíaJarmo AlanderVeronica PazziJaromír MoravecVeronica Perez-CabezasJaromír VrbkaVeronica RossanoJaroslav KováčVeronica SberveglieriJaroslav ReichelVerónica SerafínJaroslav VrchotaVeronika SzucsJarosław BrodnyVerrier IsabelleJaroslaw DominVeselin N. IvanovicJarosław FigwerVeselin RakocevicJaroslaw JacakVeselina BurevaJarosław Jaszczur-NowickiVesna BabićJaroslaw KrygierVessela KrastevaJaroslaw KrzywanskiVicente Duran-BoschJarosław MagieraVicente Faus-MatosesJarosław PrzybyłVicente FeliuJarosław PytkaVicente González RuizJarosław SzrekVicente J.P. AmorimJaroslaw TegowskiVicente LucenaJarosław WajsVicente Romo PérezJaroslaw WatrobskiVictor BlochJarosław ZalewskiVictor Bocos-BintintanJarosław ZubrzyckiVictor BolbotJaroslaw ZygarlickiVictor ChongJascha KolbergVíctor Corcoba MagañaJasenka Gajdoš KljusurićVictor Farm-Guoo TsengJasmina LukinacVíctor Gómez‐MayordomoJasmina VidicVictor GuerraJason BarkerVictor H. BenitezJason BrownVictor Hugo Rohden PrudenteJason FriedmanVictor IlisieJason G. ZeibelVictor JoosJason J. GormanVictor KallenJason M. HiteVictor Manuel González SuárezJason M. WhyteVíctor Manuel Rivas SantosJason PoulosVictor PetrovJason RambachVictor RamosJasper ReenaldaVíctor Ruiz-Valdepeñas MontielJasreman SinghVictor SanchezJasvir DalalVictor VlădăreanuJaume AngueraVictor ZhuravlevJaume BacarditVictoria HodgeJaume Coll-FontVictoria Ramos GonzálezJaume Francisco-PascualVidal MorenoJaume VerdVignesh ShanbhagJavad RostamiVijay AnandJavad ZarrinVijay KakaniJavier A. De La TejeraVijay Kumar SharmaJavier BurguésVijayakumar AnandJavier Díez-GonzálezVijaykumar B. VarmaJavier E. Contreras-ReyesVijaykumar RajasekaranJavier García CastellanoVikas KumarJavier Garcia-CasadoVikas SharmaJavier Garcia-GuzmanVikrant BhatejaJavier Gomez-PilarViksit KumarJavier González-RochaViktor MedvedevJavier López-RandulfeViktor SkrickijJavier Martínez TorresViktor VabsonJavier Medina QueroVilfredo De PascalisJavier MonroyVille KaajakariJavier Moreno-ValenzuelaVille PulkkiJavier NunoVilma RatautaiteJavier Pereira LoureiroVinaya BasavarajappaJavier Pérez FernándezVinayakumar RaviJavier Ramos-OrtegaVincent E. LambertiJavier Reina-TosinaVincent Pey ChungJavier RiedemannVincent SiebenJavier RoalesVincenzo BonaiutoJavier RocherVincenzo DentamaroJavier Rodrigo-IlarriVincenzo DeufemiaJavier RosVincenzo EramoJavier Rubio LoyolaVincenzo GallelliJavier Sanchez GalanVincenzo GrassiaJavier SilvestreVincenzo LoschiavoJavier Silvestre-BlanesVincenzo MallardoJavier ToledoVincenzo Manuel MarzulloJavier UrracaVincenzo Mariano MastronardiJavier VázquezVincenzo Pasquale GiofrèJavier Vázquez-CastilloVincenzo QuinziJavier VillalbaVincenzo RussoJawad AhmadVincenzo SerlengaJawed QureshiVineet VajpayeeJay A. WeitzenVinh NguyenJay L. NadeauVinicius KartnallerJay WadhawanVinícius MaranJayachandran VenkatesanVinicius RofattoJaydip DesaiVinod BelwanshiJayme Garcia Arnal BarbedoVinod KumarJayne WuVioleta LazicJe Hyung JungVioleta PoenaruJea Soo KimVioleta SimionJea Uk LeeViorel MinzuJean Claude De MauroyViorel PaleuJean D. Hallewell HaslwanterViraj MuthugalaJean Jacques LoiseauVirgil DobrotaJean Michel LétangVirgil DumbravaJean SequeiraVirgil OptasanuJean-Baptiste RenardVirgil-Florin DumaJean-Baptiste ThomasVirgínia Fernandes MotaJean-Charles BeugnotVisar FarhangiJean-Charles BolomeyVishal SinghJean-Claude CharrVisnja StepanicJean-Francois VuillaumeVitali F. NesterenkoJean-Jacques PoncianoVitalii A PavlovJean-Luc SeguinVitaly LevashenkoJean-Marc RibeiroVito ErricoJean-Marc TullianiVito FernicolaJean-Marc ZinggVito ImbrendaJean-Marie SleewaegenVito MonacoJean-Michel FriedtVitoantonio BevilacquaJean-Michel MartinezVitor CaldeirinhaJean-Michel RomanoVítor João Pereira Domingues MartinhoJean-Paul CalbimonteVitor N. CoelhoJean-Paul LinnartzVítor ViegasJean-Philippe AnsermetVittoria BruniJean-Philippe DidonVittoria PerrottiJean-Philippe RobergeVittorio B. LippiJean-Philippe TarelVittorio Di FedericoJean-Pierre BarriotVittorio MemmoloJean-Pierre CorriouVittorio MerloJean-Pierre HuignardVittorio RampaJean-Pierre JesselVittorio SolinaJean-Pierre RaulinVivek KumarJee Woong ChoiVivek T. RathodJeeyoung LimVivi AnggrainiJefferson AraujoVivian LoftnessJefferson Santos De GoisViviana MulloniJeffrey A. TuhtanViviane PillaJeffrey BuckleyVlad Laurentiu DavidJeffrey C. F. HoVlad PopescuJeffrey KilbyVladan PapicJeffrey S. HallamVladana GrabezJeffrey S. StevensonVladeanu CalinJehad AliVladimir A. SreckovicJehan-Antoine VayssadeVladimir AnfinogentovJehwan HwangVladimir BochenkovJelena Kilić PamukovićVladimír BulejJelena MuncanVladimir D. KuptsovJen-Chieh LiuVladimir HeiskanenJen-Chuan TungVladimir I. ZverevJeng-Ren DuannVladimir Klimentievich KachanovJeng-Shin SheuVladimir KnyazJennifer DaviesVladimir KodkinJennifer GubitosaVladimir KolesovJennifer HowcroftVladimir KrishtopJennifer M. VojtechVladimir KrivovichevJennifer SymonsVladimir KukushkinJenn-Kaie LainVladimir KutsJenny Benois-PineauVladimir KutuzovJenny HuangVladimir MedvedJenny PaggeVladimir MirskyJens Christian AndersenVladimir Nikolaevich KostinJens HartmannVladimir Nikolayevich SolovievJens HillerVladimir PitschmannJens JensenVladimir PopokJens KirchnerVladimir PopovJens M. HovemVladimir PoulkovJens MuehlsteffVladimir RajsJens NeuVladimir RogalewiczJens V. FischerVladimir SergeyevJenu ChackoVladimir ShakhovJeon LeeVladimir ShepelevJeong Min KangVladimir SkripnyakJeong Ryeol ChoiVladimir SobeslavJeong Su LeeVladimir SolovievJeongguk KimVladimir StankovicJeonghoon KwakVladimir SukhovJeonghyun KimVladimir TadicJeong-Jung KimVladimir TadićJeongmoo HuhVladimir V. AndreevJeongsik ChoiVladimir VavilovJeongyeup PaekVladimir VillarrealJeremie HoussineauVladimíra BiňasováJeremie SublimeVladislav DemyanovJeremy AdlerVladislav KalinnikovJérémy VézinetVlastimil MasekJeroen BergmannVlastimil MatejecJeroen DegerickxVojtěch PetruchaJerome AntoniVolker CimallaJérôme CosteVolker DworakJérôme De MirasVolker RodehorstJérôme IdierVolkov PetrJérome LucVolodymyr GurskiJérôme MolimardVolodymyr PonomaryovJérôme RossignolVrchota JaroslavJérôme Tchoufang TchuindjangVuslat B. JuskaJérôme TissierVytautas BučinskasJerry Jialie ShenWaad SubberJerzy A. SładekWacław DziurzńskiJerzy BakunowiczWael Abdullah AhmadJerzy BalickiWafa M. BataynehJerzy HapanowiczWagner Coelho De Albuquerque PereiraJerzy J. JasielecWagner Tanaka BotelhoJerzy J. LangerWahyu CaesarendraJerzy JozwikWai Lok WooJerzy KonorskiWai-keung FungJerzy MajkaWaldemar DołęgaJerzy MikulskiWaldemar JendernalikJerzy PotenckiWaldemar KociubaJerzy SiuzdakWaldemar OdziemczykJerzy ŚwiątekWaldemar RączkaJerzy WinczekWaldemar ŚwiderskiJerzy WtorekWalter GrondzikJesper RasmussenWalter J. SalcedoJesse Yoe Rumbo MoralesWanda ForczekJessica Ramella-RomanWanfeng DouJéssica Sena De SouzaWang JianjiangJessie T. ChildsWang TianJesus Cruz-GarzaWanguo JiaoJesús De Vicente Y OlivaWanjung ChangJesús Donaire-AvilaWanke LiuJesus E. Velazquez-PerezWankun XieJesus Enrique SierraWanli LiuJesús Fernández-HernándezWann-Yun ShiehJesus G. Cruz-GarzaWansoo NahJesús Grajal De La FuenteWanxin WenJesús López BelmonteWaqar AhmadJesus LozanoWaqas AhmedJesús M. Gómez-de-GabrielWard Van Der TempelJesus Manuel Gomez-de-GabrielWassim AlexanJesús Manuel Nieto GarcíaWataru SatoJesus Mora-RodriguezWei GaoJesús Oliva-Pascual-VacaWei GuanJesús OrtizWei HuJesús PastorWei JingJesús Pérez-RíosWei LiJesus Roberto Millan AlmarazWei LinJesús Salido TerceroWei LiuJesus SalinasWei MaJesus Sanchez-GomezWei OuyangJesús Sergio Artal-SevilWei PuJesus TejadaWei QiuJesús-Ignacio PrietoWei ShaoJetmir HaxhibeqiriWei ShenJevgenijus ToldinasWei SongJeyeon KimWei TangJH ChoWei TaoJhantu Kumar SahaWei WangJhe-Syuan LaiWei WuJhon Castaño-OsorioWei YanJhon Silva-CastroWei YangJi Bo WenWei YaoJi FanWei YiJi HanWei ZhangJia JinWei ZhaoJia LiWei ZhengJia RongWeibin LiJia ShiWeibing GuJia UddinWeibing ZhongJia WuWeibo LiuJia XuWeichao ZhuangJiabao YuWei-Che ChienJiachao ZhangWeicheng CuiJiachen YangWei-Chiang HongJiachou WangWei-Chiang WuJiafeng GuWeidang LuJiafeng XieWeidong YangJiafeng YaoWeifeng PanJiahao YanWeiguang ZhangJiahao ZhongWeiguo FangJiahong ChenWei-Han ChenJiahuan JiangWeihe XuJia-Jung WangWei-hsiung YangJiakai LiWei-Hsun LeeJiali YinWeihua DongJialun LiuWei-Hua HuJiamin SunWeihua LiuJiaming LiangWeihua PeiJiaming NaWeijie FuJian GaoWeijie LiJian GeWeijie LiuJian HuangWei-Jiun SuJian JiaoWeili DengJian KangWeilin LuoJian MaWeilin YeJian SunWei-Lung TsengJian WanWeiping LiJian WangWeiping ZhuJian Wei TayWeiqi HuaJian WengWeiqiang MaJian XuWei-Rong LiuJian XueWeisheng ChiuJian YangWei-Ting LinJian ZhangWeiting LiuJian ZhouWeiwei LiuJianbin GaoWeiwei SunJianbin QiuWeiwen ZhangJianbin XinWeixi GuJianbing XieWeiyan HouJianbo QiWeizhi MengJianbo WuWellington Pinheiro Dos SantosJiancheng TaoWen Chang HuangJiande SunWen LiuJiandong RenWen SunJianfa ZhangWen XiaoJianfeng LiWen ZhangJianfeng PingWenbin YuJianfeng RenWen-Chang TsaiJianfeng WuWen-Chung TsaiJianfeng ZhaoWenfang LuJiang LiuWengang WuJiang ZhaoWengang ZhangJiangbo PuWen-Hsi ChengJiangbo XiWenjian HuaJiangcheng LiWenjin WangJianghai XiaWenjin WuJianghong MaoWen-Jong ChenJianglei DiWenjuan SunJiangsheng GuiWenjun (Chris) ZhangJiangtao XuWenkai LiJianguo ChenWenlin GongJianguo DingWenming ChenJianguo LiuWenpeng LiJianguo LuWenqi RenJiangwei TianWenqiang ZhangJianhai ZhangWen-Sheng ZhaoJianhua LiuWensong JiangJianhua WuWentao LiJianing LiWentao ZhangJia-Ning LuoWenxin DaiJian-Jang HuangWenxin TianJianjia WangWen-Ya LeeJianjun MaWenyi WangJiankang WuWenyu CaiJiankun WangWenyuan CuiJianlai ChenWenyuan YangJianlin GuoWenzeng ZhangJianming WenWenzhuo WuJianming ZhangWerner HaselmayrJianping LiWerner LienhartJianqi WangWerner MänteleJianqiang QianWeslania Viviane NascimentoJianqing WuWesley BakerJiantao YaoWesley T. HoneycuttJianwu FangWiktoria WojniczJianwu ZhangWilfried GappmairJianxi LiuWilfried UhringJianxin JiaWilhelm Robert GroszJianxin YiWilliam C. WilsonJianxing LiuWilliam D.G. BrittainJianxiong ZhuWilliam HeadleyJianyi LiuWilliam HurstJianyu FuWilliam L. RaffeJian-Zhang ChenWilliam RomineJianzhen OuWilliam StewartJianzhong LiWilliam Y. Y. ChengJianzhong ZhangWilliam ZimmermanJiaqi HanWim EctorsJiaqi YangWinston PercybrooksJiaqing XiongWioletta Ochędzan-SiodłakJiarui HuangWisit CheungpasitpornJiarui LinWitold CzajewskiJia-Shiun ChenWitold KazimierskiJiawang ChenWładysław SkarbekJiawei ChenWłodzimierz KasprzakJiawei HuangWojciech DawidowskiJiawei XiangWojciech FabianowskiJiawen XuWojciech JadwicienczakJiaxin LiuWojciech KaczmarekJiaxing CheWojciech KnapJiayi MaWojciech SałabunJiayong YuWojciech SasJiayu LiWojciech SkieruchaJiayuan HeWojciech SkrzeczanowskiJiba DahalWojciech SteczJibin ZhengWojciech SumelkaJibran KhaliqWojciech SzelągJichao HongWojciech TutakJichiang TsaiWojciech WitkowskiJidong YangWojciech WolanśkiJie CaoWojciech ZabierowskiJie ChengWolfgang BacsaJie CuiWolfgang CaliebeJie DengWolfgang FuhlJie HuWolfgang HübnerJie HuaWolfgang LeisterJie HuangWolfgang PotthastJie LiWolfgang SchreinerJie LingWolfram LutherJie QiWolfram VolkJie YangWon-Du ChangJie ZhangWon-Jae RyuJiejian DaiWonjin NaJiewu LengWonjun KimJieyun WuWonseok LeeJifang TaoWonzoo ChungJifeng LiuWoo June ChoiJihong KimWoo-Kyung SunJihoon KimWoonghee LeeJihoon LeeWoo-Suck HanJihoon MoonWooyeol ChoiJi-Hoon YunWoubishet Zewdu TaffeseJihun KimWouter Van VerreJihyoung ChaWu DengJihyun BaeWu XiaoJijun RenWu-Cheng ChiJiliang ZhangWujun SiJimin MaengWuyi MingJimmie MillerWuyi WanJimmy OlssonWycislik LukaszJims MarchangWynand JvdM SteynJin ChenX P V MaldagueJin HeXabiel García PañedaJin Kyu GahmXanthoula Eirini PantaziJin LiXavier BoddaertJin LiangXavier FernandoJin NingXavier GasparuttoJin QiXavier VilanovaJin Sik KimXénia LatypovaJin Su JeongXenofon StrakosasJin TaoXi GuJin WangXi PengJin WenXi QiaoJin YanXi ShiJin YangXi YangJin Young LeeXia MinJin ZhangXiadong WangJinao ZhangXian JianJinbo WuXian LiuJinchao ChenXian SunJinchao TongXian TaoJinchen JiXianbo WangJincheng LeiXianfeng YuanJindong SongXiang ChenJindřich LibovickýXiang ChengJindrich LiskaXiang LiJinfeng LiXiang WuJinfu LiuXiang XieJing ChenXiang XuJing FanXiang ZhangJing JinXiangang LuoJing LiXiangbo KongJing LiuXiangcheng ChenJing Selena HeXianghong WangJing TengXiang-Jun ZouJing TianXiangping ChuJing WangXiang-Tian KongJing WeiXiangxin LiJing XuXiangxiong KongJing YanXiangyang JuJing YangXiangyang XuJing YueXiangyin ZhangJing ZhangXiangyu XuJing ZhouXianjian JinJingang ZhongXianju LiJingbiao ChenXianjun HaoJingbo ZhaoXianta JiangJingchen WangXianyi ZengJingcheng ZhouXianzheng ZongJing-Chie LinXianzhi WangJingfei LiuXianzhong ChenJingfeng HuangXiao Dong ZhouJing-Hui QiuXiao FuJingjing HeXiao LiJingjing LuoXiao LiangJingjing XuXiao LiuJinglei TangXiao WangJinglin ZhangXiao WuJinglong ChenXiao YongJingru YiXiao ZhouJingshui HuangXiaobing DaiJingsong LiXiaobing LiJinguang CaiXiaobo RuiJingya LiXiaochao DangJingyu WangXiaochen ZhangJingyu YangXiaochen ZhengJingZheng HuangXiaodan XiJin-Hak YiXiaodong LuJinho YoonXiaodong YangJinhong GuoXiaofei DengJinhua SunXiaofei LiJinhua YuXiaofeng GaoJinhyeong KwonXiaofeng LiJinjin YanXiaofeng LuJinkui ChuXiaofeng WangJinlian HuXiaofeng YuanJin-Liang HeXiaogang DengJinling ZhaoXiaoguang DongJinlong FanXiaohong SuiJinming LiuXiaohong ZhouJinnan ZhangXiaohua HaoJinn-Min YangXiaohui HanJinping SunXiaohui YangJinquan LiuXiao-hui YuanJinsheng YangXiaojing MuJinsil LeeXiaojun TangJinsong WuXiaojun YuJinsuk BaekXiaokai WangJinwei SunXiaolei YangJinwen MaXiaoli XiJinwoo JangXiaoliang GuoJin-Woo JeongXiaoliang MengJinwoo ShinXiaoliang ZhuJinwook KimXiaolin ChangJinwu KangXiaolin MaJinxi GuoXiaoling ZhangJinxin HeXiaoman LuJinxin LiuXiaoming GaoJinxing QiuXiaoming LiuJinyang DuXiaoming WangJinyang LiangXiaoming WuJinyang ZhangXiaoning JiangJinyi LiXiaopeng LiJin-Yuan WangXiaoping LiaoJinzu JiXiaoping LiuJiri BarekXiaoqiang HuaJiři ErhartXiaoqiang LiJiří JandaXiaoqing BaiJiří KrejsaXiaoqing GaoJiri PetrzelaXiaoqing TianJiří PflegerXiaorong ZhuJiri PlasekXiaoshan BaiJiří PřibilXiaosheng ZhangJiri TesarXiaoshuang ChenJiří VackářXiao-Song ZhuJiseok LimXiaotong ZhangJitae ShinXiaowei LiuJitang FanXiaowen XuJitka KumhálováXiaoxing HeJiuhou LeiXiaoyang Rebecca LiJiuk JangXiaoyi BaoJiulu GongXiaoying GuoJiun-in GuoXiaoying ZhuangJiun-Jian LiawXiaoyong ChenJiun-Yu SungXiaoyu ChengJiushuai XuXiaoyu WanJivitesh SharmaXiaoyuan WangJiwei HuangXiaoyuan ZhuJiwen CuiXiaozhen ZhouJi-Won SeoXi-chuan LiuJiwoo KangXignpo MaJi-Wook YoonXimeng ChengJiyong HuXimeng LiuJiyoung JungXin ChengJize ZhangXin DuJizhong SongXin GaoJo VerhaevertXin LiJoab R. WinklerXin LinJoachim ClemensXin LiuJoachim EndersXin NingJoachim IngwersenXin ShanJoachim JonuscheitXin WangJoachim WiestXin ZhangJoan BasXin ZhongJoan BordoyXin ZhuangJoan CabestanyXinbin LiJoan Claudi SocoróXincong YangJoan Leal-BlanquetXing FuJoan Navarro MartínXing MaJoan Pons-LlinaresXingchen JiJoan Ramon CasasXingchen MaJoan RossellóXingchen YangJoana CostaXinge YuJoana FigueiredoXingfu ZhangJoana GuedesXinghua LiJoana M. WarneckeXinghua YangJoan-Francesc Fondevila-GascónXingjian LiuJoanna AdamczykXingliang HuoJoanna BorowiecXingling ShaoJoanna CabajXinglong JuJoanna DipnallXing-Ming GuoJoanna GrygierekXingming LiangJoanna IwaniecXingquan CaiJoanna JanickaXingran CuiJoanna Jaworek-KorjakowskaXingyu GuJoanna KonopińskaXingyu LinJoanna Kos-ŁabędowiczXinheng WangJoanna LiszkowskaXinhua MaoJoanna Maria DulińskaXinke LiJoanna OrtylXinli DuJoanna Paciorek-SadowskaXinming LiJoanna Przeździecka-DołykXinMing ZhangJoanna Sekulska NalewajkoXinqiang ChenJoanna SobiakXinqin LiaoJoanna ZajdaXinqun ZhuJoao A. LorenzzettiXinrui DingJoão AbrantesXinyu GuJoão AlmeidaXinyu LiJoão BarataXinyu ZhaoJoão C. NevesXinyuan WeiJoão CaladoXinyue ShenJoao Carlos Amaro FerreiraXinzhong LiJoão Carlos FerreiraXiong YangJoão CastelhanoXiongbo DuanJoão CoelhoXiping HeJoão FelícioXiqian JiangJoao FerreiraXiu LiJoão Filipe Fernandes Lindo SimõesXiuhong LiJoão Flávio Da Silveira PetruciXiujuan ChaiJoão KleinschmidtXiukun WeiJoão LeitãoXiuping LiuJoão M. F. CaladoXiuwen FuJoão M. L. P. CaldeiraXiuyou HanJoão Miguel da Costa SousaXiyu ShiJoao Miguel SousaXoan Xosé Fernández Sánchez-RomateJoao MoreiraXosé Antón Vila SobrinoJoão MouroXosé Luís Deán BenJoao Nuno MatosXose M. Lopez-FernandezJoão Nuno MatosXu An WangJoao PalottiXu BaiJoão PatrícioXu DingJoão Paulo BarracaXu QiaoJoão Paulo CarmoXu TangJoão Paulo Carvalho Lustosa Da CostaXuan SunJoao Paulo FloresXuan ZhuJoão PereiraXuchun GuiJoão Pinto CoelhoXudong JiaJoão ReisXudong LiJoão Ribeiro PintoXudong ZhangJoão Rocha VazXudong ZouJoao Ruivo PauloXue HanJoão Silva SequeiraXuebin RenJoão Vilas-BoasXue-Bo JinJoaquim AzevedoXuedong ZhangJoaquim C.G. Esteves Da SilvaXuefeng HuJoaquim ChalerXuefeng ZhangJoaquim De MouraXuejian WuJoaquim FerreiraXuejin LiJoaquim Miguel MaiaXuejing WangJoaquim Minguella-CanelaXuejun ZhangJoaquim SousaXuemei ZhouJoaquim TinocoXuemin ChenJoaquin ArandaXuesong YanJoaquín Gayoso-CabadaXuewen WangJoaquin GonzalezXueyi ShangJoaquin L. HerraizXuezhi WangJoaquín LuqueXuezhi WuJoaquin MartinezXuguang LanJoaquin Paez-MoguerXukun YinJoaquin SanchizXun DouJoaquín Torres-SospedraXuyang ZhangJobst WurlXuyuan TaoJoceli MayerXxiaorui ShaoJochen HillerYadong ZhouJochen LangYael NemirovskyJochen ManaraYafeng YinJochen SeitzYago DiezJoe WiartYahui PengJoel Antonio Trejo SánchezYahya MezianiJoël CollocYahya RharbiJoël LemaireYajun LiuJoep Van Den BroekeYakoub BaziJohan CasselgrenYakushenko AlexeyJohan J. Estrada-LópezYalan LiuJohan Jair Estrada-LópezYan HongJohan MarkdahlYan HuangJohan N Van Der MeerYan LiJohan Van Der MeerYan LiuJohann KastnerYan MaJohann MartonYan Naung SoeJohannes BetzYan (Rockee) ZhangJohannes FresnerYan ShiJohannes P. Van DijkYan WangJohannes RothsYan ZhangJohannes VorwerkYanbin WangJohannes W. PassandYanbo NiuJohannes WinterYanbo PanJohari Yap AbdullahYanbo YangJohn A. MercerYanchao SunJohn AbelaYanchao ZhangJohn AdamovicsYanfeng JiangJohn AllenYanfeng ShenJohn C.-C. LuYang HuJohn D. KechagiasYang Jun KangJohn DermentzoglouYang KailuJohn EnrightYang KuangJohn G. KellettYang LiJohn GialelisYang LinJohn GountasYang LouJohn HansenYang LuJohn J. GuersYang RanJohn Jairo Villarejo MayorYang ShaoJohn JelonnekYang ShenJohn M. AbendrothYang ShuangJohn M. AckenYang SongJohn McCamleyYang WangJohn McNuttYang XuJohn Michael RettererYang YangJohn Murray-BruceYang YuJohn NassourYang YueJohn RebulaYang ZhaoJohn S. MitchellYangchun ChengJohn SaffellYang-han LeeJohn ThomasYanglong LuJohn TsiligkaridisYangmao WenJohn Withers WalkerYangong ZhengJohn-Lewis Zinia ZaukuuYang-wei LinJoice MathewYang-Xin YuJolanta KarpowiczYangyang HeJolanta PaukYanhu ZhangJon AshleyYanhua LuoJon Glenn GjevestadYanjie JiJon Legarda MaconYanjie SuJon YounYanjie ZhouJonas H. OsórioYanjun YanJonas MatijošiusYanki AslanJonas NeuserYankui SunJonatan LergaYanliang HuangJonathan BrembeckYanlin GengJonathan EngelYanling DuJonathan EvansYanmin ZhangJónathan HerasYannick SalaminJonathan RealmutoYanpeng CaoJonathan ResopYanping WangJong Il RheeYanping XuJong Moon HaYansheng LiJong Yong LeeYanshun ZhangJong Yun LeeYanwei LiuJongbeom KimYanxia HouJongBeom LimYao GuoJong-Chan KimYao MaoJongCheol PyoYao ShenJong-Chul LeeYao SongJong-Ho ShinYao ZhangJonghoek KimYaodong GuJong-Hoon WonYaofei ChenJong-Il ParkYao-Feng ChangJong-Jin ParkYaokai FengJongkil KimYaoli WangJongmin Andrew YuYaolu LiuJongmin LeeYaoxiang LiJongmin MoonYaping CaiJongmin ParkYaqing LiuJong-Myon KimYary VolpeJongpil JeongYasir Al-YasirJong-Ryul YangYasir Beeran PottatharaJongsang SonYasir LatifJongshill LeeYasmen Ahmed Abd El Kader KamelJongsub MoonYasmin FathyJong-Won ParkYassine ChaibiJongwook JangYasue MitsukuraJong-Wook LeeYasuhiro AkiyamaJongwoong ParkYasuhisa OmuraJonqlan LinYasuomi IbarakiJoo Chuan YeoYasushi ShimadaJooeun AhnYasutaka KitahamaJoohan KimYau-Zen ChangJoonas IivanainenYawen GuanJoonas MultanenYazan A. AlqudahJoong Ho ShinYe MaJoong-Wook LeeYe ShaJoonha JungYe ZhouJoonho SeoYee Loo FooJoon-Yong LeeYee Yan LimJoonyoung KimYeejin LeeJooyong KimYeh Hsi-Jen JamesJordi CusidoYejin KimJordi Fonollosa MagrinyaYelena R. SliozbergJordi MallorquiYemin GuoJordi Mongay BatallaYen Ting ChenJordi OrtizYenny Villuendas-ReyJordi PalacínYeo Jin KimJordi SacristanYeong Hwan KoJordi-Roger RibaYeonsik KangJörg BlankenbachYeou-Jiunn ChenJörg DomaschkaYew Hoong WongJörg PezoldtYi BaoJorg RadnikYi CuiJorge Alfredo Ardila-ReyYi FangJorge Antonio Silva CentenoYi LeiJorge BernardinoYi LuJorge BrievaYi LuoJorge C. S. CardosoYi QinJorge Caceres-GianniYi WangJorge Calvillo-ArbizuYi XuJorge CamachoYi YangJorge Cerqueiro-PequeñoYiannis KantarosJorge Cesar MasiniYi-Bing LinJorge DiasYichao ZhaoJorge E. Ibarra-EsquerYi-Chen ChenJorge E. Jimenez-HorneroYi-Chen LiJorge E. LuzuriagaYicheng LiaoJorge EscorihuelaYichuan DengJorge Fernández-BerniYichuan WuJorge FeuchtwangerYichun DingJorge Galván-TejadaYidong TanJorge Garcia-MarquezYifan LiuJorge GosalbezYifan LuJorge GuerraYih JengJorge HenriquesYihai HeJorge Herrera-TapiaYihong QiJorge IgualYihua TanJorge LanzaYi-Hung LiaoJorge López-FernándezYijin PanJorge Luis Camas AnzuetoYik-Chung WuJorge Luis García-AlcarazYi-Kuang YenJorge Luís Victória BarbosaYi-Lang ChenJorge Luis Zambrano MartinezYili ZhengJorge Maestre VidalYilong HuiJorge Marcos-AcevedoYilun ShangJorge Mario Cruz-DuarteYiming HuoJorge Paredes VieyraYiming ZhuJorge Pérez-GómezYin BaoJorge Ricardo Mejía-SalazarYin ZhangJorge RoperoYi’nan HeJorge Sanjurjo-SánchezYing DongJorge TobonYing FuJorge Torres SánchezYing LiuJoris PascalYing MaoJorn MehnenYing MuJos ElfringYing TanJosé A. OrosaYing TieJosé A. PeláezYing WangJosé A. RabadánYing-Chih LaiJosé A. Siqueira DiasYing-Chih LiaoJosé Alberto Batista RodríguezYing-Hao YuJose Alberto Benítez AndradesYingjie XiaJosé Alberto GonçalvesYinglei TengJose Alejandro Galaviz-AguilarYingnong ChenJosé Alons GómezYingpei ZengJose Angel Corbacho MerinoYingpeng SangJose Antonio Alvarez-BermejoYing-Ren ChienJose Antonio Barrera-VeraYingrui ShangJosé Antonio De La O SernaYingtao LiuJosé Antonio FilipeYingwei LiJosé Antonio García NayaYingying WangJose Antonio Garcia SoutoYi-Ning WuJose Antonio Gazquez ParraYinji MaJosé Antonio González PrietoYinlai JiangJose Antonio Lozano-GalantYinnian FengJosé AzinheiraYinsheng ChenJose Balsa-BarreiroYin-Tsung HwangJosé Benito Bouza-RodríguezYinwei ZhanJosé BritoYiran LuoJosé C. Ramírez-FazYi-Ren WangJosé CampeloYirui WuJosé Carlos Fernández-GarcíaYishou WangJose Carlos MetrôlhoYishuo HuangJosé CecílioYisong TanJosé ChiloYiting KangJosé Cleiton Sousa Dos SantosYiwei ShiJosé Cleydson F. SilvaYixing DingJose Costa-RequenaYixuan FengJosé Daniel Hernández SosaYiyan FeiJosé DarrozesYiyan LiJose De Jesus Agustin Flores CuautleYiyang ChenJose De Jesus RubioYiyang DaiJose DuenasYi-Yung ChenJosé Emilio Guerrero-GinelYizhi LiuJose Emilio MeroñoYizhou ZhuangJosé Fidel Baizabal-CarvalloYngvar LarsenJosé Francisco Gómez-AguilarYoav LinzonJose Francisco Saenz-CogolloYogang SinghJose Francisco Tornero-AguileraYogeenth KumaresanJose Franco Da Cunha Leme FilhoYohei KurataJosé GonçalvesYohei MatsumotoJosé Guillermo Collí AlfaroYolanda Blanco-FernándezJosé I. De La RosaYong GuJose Ignacio Lijarcio CarcelYong Han AhnJose Ignacio Priego QuesadaYong HuangJosé Ignacio Rodríguez MolanoYong Ju JungJosé Ignacio SerranoYong LiJosé Israel Martínez-LópezYong Oh LeeJose Jaime Camacho-EscotoYong PanJosé Jair Alves Mendes JuniorYong ShangJosé Javier Astrain EscolaYong Soo ChoJosé Javier Reyes-LagosYong WanJose Juan Arranz-JustelYong WangJose Juan Garcia-HernandezYong XuJose L. García-SoidánYong YangJosé L. HernándezYong ZhangJosé L. PastorYong ZhaoJosé LázaroYong ZhuJose Luis Cruz-MuñozYongbin LiuJosé Luis Díaz-HernándezYongchuan TangJosé Luis FelipeYongcun HaoJosé Luis Hernández RamosYongfeng LiuJosé Luis Hernández-HernándezYonggang HuJosé Luis LimaYonggang XiaoJosé Luis López-MartínezYong-Gyoo KimJosé Luis OlazagoitiaYongho ChoiJose Luis Outón MéndezYonghua QuJose Luis PuraYong-Hwa ParkJose Luis Sanchez-RojasYongjia XuJose Luis UbagoYongjie ZhuJose Luis ValenzuelaYong-Jin LiuJose Luis Vazquez-AvilaYong-Joo ChungJose Luiz Sousa LimaYong-Joo KimJose M. Cabeza-LainezYongjun ZhengJose M. ClaverYongkai YinJose M. JimenezYongli ZhuJose M. Jimenez-OlmedoYongliang QiaoJosé M. SigarretaYongliang WangJosé Machado Da SilvaYongming LiJosé Manuel Almerich-SillaYongning WuJose Manuel AndradeYongqiang HaoJosé Manuel CabedaYongrae RohJose Manuel Cano GarciaYong-Sang KimJose Manuel Cano-GarciaYongseok JeeJose Manuel De AlmeidaYong-Seok JeeJosé Manuel Díaz-CruzYong-Seok LeeJose Manuel Garcia AlonsoYongsu ParkJose Manuel Gimenez-GuzmanYongzhen HuangJosé Manuel Mirás-AvalosYoo Min ParkJosé Manuel M.M. De AlmeidaYoon-Sik YooJosé Manuel ParedesYoon-Soo JangJosé Manuel QueroYooseob SongJosé Manuel Sáez LópezYo-Ping HuangJose Manuel Soto HidalgoYorgos StephanedesJosé Manuel VilladangosYosef PinhasiJosé MarcatoYoshiaki AdachiJosé Marcato JúniorYoshifumi SaijoJosé Marcos OrtegaYoshihiro FukumotoJosé María Cámara-ZapataYosi KristianJosé María EnguitaYosi Shacham-DiamandJosé María Garrido BalsellsYosoon ChoiJosé María López GullónYosuke AokiJosé María Martínez-OtzetaYosuke SugiokaJosé María Palacios-SantanderYoubin ZhengJose Maria Sebastian Y ZuñigaYougan ChenJosé María Senciales-GonzálezYougang BianJosé Mario De MartinoYougang SunJosé Martinez-CarranzaYougen YiJosé Martínez-LilloYoukou DongJosé MaurícioYoukyung HanJosé Miguel López-HigueraYounes JabraneJosé Miguel MotaYoung Chang JoJose MillaYoung Chul LeeJosé Naranjo-TorresYoung Doo LeeJose Navarro-PedreñoYoung Eun SongJosé NovoaYoung Min SongJosé Paulo Ferreira LousadoYoung Soo SuhJosé Pedro AmaroYoung Sub KwonJosé Pelegrí-SebastiáYoungbae HwangJose Priego-QuesadaYoungchul BaeJose R. Guzman-SepulvedaYoung-Dam KimJosé R. LlataYoung-du LeeJose R. ValverdeYoung-Duk KimJosé Raduan JaberYoung-Gun ChoJosé Rafael García-SánchezYoung-Hwan YouJose Ramírez-FazYoungjin ChoiJosé Ramón Gaxiola-CamachoYoungJin JungJosé Ramon Lillo-BeviáYoungjoon HanJose Ramón Quevedo PérezYoung-Ju KimJosé Ramón Saura LacárcelYoungoh LeeJosé Roberto CardosoYoungok KimJosé Rodríguez-AviYoung-Pil KimJosé SaenzYoung-Seob JeongJosé SaiasYoung-Sik KimJosé SantaYoungsook BaeJosé SantosYoungsun RyuhJose Torres FarinhaYoungwook SeoJosé Vicente Francés-VilloraYousaf Bin ZikriaJosé Vicente MartíYousef Rezaei TabarJosé VilcaYoussef BichiouJoseb BlazekYoussef RouphaelJosef BlažejYouwen ZhangJosef BuchnerYouyang QuJosef PiheraYouzhong LiuJosefina GutiérrezYu CaiJose-Maria Serrano ChicaYu CaoJosep AlosYu CherepennikovJosep Lluis Rosa EstevaYu FujimotoJoseph AzarYu HanJoseph BevittYu Hen HuJoseph Chang Lun ChanYu KumeJoseph ChazalonYu Kyoung RyuJoseph HupyYu LiJoseph KvedarYu LiuJoseph M. FortunakYu MatsudaJoseph M. MahoneyYu MiaoJoseph NassifYu SongJoseph NdieyiraYu TakJoseph NgYu TangJoseph P. HornakYu Ting TsaiJoseph SniderYu Tzu WuJoseph SpernyakYu WuJoseph VerfaillieYu ZhangJoseph W. LowdonYuan MaJoseph WasswaYuan MengJosé-Victor RodríguezYuan PingJosh JesselYuan WuJosh SugarYuanchang ChenJoshi NeerajYuanchao LiuJoshua EnsworthYuancheng FanJoshua P. BaileyYuan-Chien LinJosias BatistaYuan-Ho ChenJosip KasaćYuanjian ZhangJosip LorinczYuanjie SuJosip MusićYuanjin ZhengJosip PeranicYuanjing LinJosko SodaYuankai QiJosko ZecYuankai WuJounsup ParkYuansong QiaoJoydev GhoshYuanwei WuJoyraj ChakrabortyYuanxi PengJoyram ChakrabortyYuanxi SunJože GunaYuanxin YeJože KutinYuanyao LuJože Martin RožanecYuanyuan JiaJozef GnapYubin GuoJozef JuhárYu-Bo ShengJózef LisowskiYuchao BaiJózef OberYu-Chen LinJozef PapanYu-Cheng FanJozef RoehrichYucheng WuJozsef KatonaYu-Chi LeeJozue Vieira FilhoYuchu QinJu LinYu-Chun KungJu Seok RyuYu-chung HsiehJu WangYudhi AdhityaJu Wook JangYudith CardinaleJuan Antonio Alvarez-GarciaYudong LuJuan Antonio AñelYudong ShenJuan Antonio Castro-GarciaYudong ZhangJuan Antonio Guerrero-IbáñezYu-Dong ZhangJuan Antonio Holgado-TerrizaYue CuiJuan Antonio López RamosYue GuJuan Antonio Martinez NavarroYue GuoJuan Antonio Martinez RojasYue LiJuan Antonio Valera-CaleroYue LiaoJuan Arturo SquellaYue WangJuan BanderaYue WuJuan Baselga LlidóYuecheng ShenJuan Camilo Vásquez-CorreaYuefeng ZhaoJuan Carlos Alvarez AlvarezYuei-An LiouJuan Carlos Cuevas MartínezYuejian ChenJuan Carlos GrandaYuejun KangJuan Carlos Martinez EspinosaYuelei XiaoJuan Carlos MosqueraYue-Ling CaoJuan ChengYuelong ChuangJuan Cota-RuizYuemin YueJuan CruzYuen ClementJuan De Dios Sanchez-LopezYueshen WuJuan F. PrietoYue-Shi LeeJuan Francisco Dols RuizYueyun ChenJuan Francisco Fuentes-PérezYufei LiuJuan Gabriel Avina-CervantesYufei MaJuan Gerardo ÁvalosYufeng ShiJuan Gregorio Rejas AyugaYuguang FuJuan I. GuerreroYuhai BaoJuan Ivan Nieto-HipolitoYuhan HuangJuan J. CastilloYuhang LiJuan Jesús García DomínguezYuhao WangJuan Jesús Roldán GómezYuh-Chung HuJuan José De DiosYuhong LiJuan José EscobarYuhong LiuJuan Jose Galiana-MerinoYuhong ZhengJuan José Jiménez MartínYu-Hsi HuangJuan José López EscobarYuhsin HungJuan José Moyano CamposYu-Hsiu LinJuan José Murillo-FuentesYuhuai PengJuan Jose Saucedo-DorantesYuichi FujinoJuan José UnzillaYuichi OtsukaJuan Luis CarúsYu-Jen ChenJuan Luis GonzaloYuji DongJuan Luis Higuera-Trujillo Yuji WadaJuan Luis Navarro-MesaYujuan SiJuan Luis NievesYujung ChenJuan M. CalderonYukai ChenJuan M CorchadoYu-Kai WangJuan M. GandariasYuki FunaboraJuan M. SantosYuko NakamuraJuan M Sierra-HernandezYukun LaiJuan Manuel EscañoYulei QianJuan Manuel GutiérrezYulia Yu BozhkoJuan Manuel Jacinto-VillegasYuliang MaJuan María Hidalgo-BetanzosYuliang ShiJuan MelchorYuliang ZhaoJuan Mielgo-AyusoYulong GuoJuan Miguel Fernández-CampoyYuming LiuJuan Miguel Soria GarciaYun Jung HeoJuan Mora-MacíasYun LinJuan Moreno García-LoygorriYun PanJuan P. Amezquita-SanchezYun Suen PaiJuan P. Domínguez-MoralesYun ZhangJuan P. VasconezYunbin DengJuan Pablo Amezquita-SanchezYunchao TangJuan Pablo Garcia VazquezYunchen YangJuan Pablo MartínezYuncheng ManJuan Pablo PascualYundong LiJuan Pascual-GarcíaYunfeng CaoJuan PereiroYunfeng WuJuan Rodrigo Vélez-CorderoYung Hsiang ChenJuan ShanYung Po TsangJuan VillanuevaYunghui LiJuan YiYung-Keun KwonJuan ZhuYung-Sheng ChenJuan-Chen HuangYun-ho ShinJuan-Luis GorrichoYunhua RaoJuan-Pablo Ramirez-ParedesYun-Ju LeeJuarez Bento Da SilvaYunli ShaoJucielle Veras FernandesYunlong GuoJudah LevineYun-Lu SunJude HemanthYunMook LimJudicaël PicautYun-Sik NamJue HouYuntao GuoJuerg NeuenschwanderYunus EgiJüergen BrunsYun-Wei LinJuha Ala-LaurinahoYunxiang WangJuha KangasluomaYunxu SunJuha RoningYunyoung NamJuhani H. MäättäYunyuan GaoJuhász JánosYunze HeJuhyeon HongYuqi FanJui-Chiu (Rachel) ChiangYuqi LiJui-chung HungYuqing SunJui-yu WuYuren ChenJui-Yung ChangYuri D’AlessandraJukka TalvitieYuri FeldmanJules-Raymond TapamoYuri LópezJulia KowalYuri RzhanovJulia V. TsyganovaYuri S. KivsharJulián Castellanos-RamosYuri VoznyakJulián Fernando Calderón-GarcíaYuriy OrlovJulian KagerYuriy PovstenkoJulian Marius MüllerYury MarapuletsJulián Salas PiñónYury YasyukevichJulian VargheseYusen HeJulian VasilevYushan SunJulian WebberYu-Shen LiuJuliana Aparecida AnochiYu-Sheng SuJuliana Azoia LukiantchukiYustus Eko OktianJuliane R. SempionattoYusuf OzturkJuliang XiaoYusuf Valentino KanetiJulie MarteauYusuke KajiwaraJulie WallYusuke YamadaJulien LesoupleYutaka KawabeJulien MarletYutaka WatanobeJulieta Domínguez-SoberanesYu-Ting BaiJulio Cesar Rosas-CaroYuting GaoJulio E. Normey-RicoYuting ZhangJulio J. Carabias-OrtiYuvaraja TeekaramanJulio MarcoYuwei WangJulio RomeroYuwen ChenJuliusz HuberYuxia DuanJun ChenYuxiang HongJun Cong GeYuxiang SunJun Dong ChoYuxin HuJun FangYuxin LengJun HongYuxin XingJun HuYuxing HanJun KawakamiYuxing LiJun LiYuxing WangJun LinYuyang WeiJun LiuYves DubéJun Long LimYves GourinatJun MaYves MahéoJun Min SuhYves ReulandJun MouYvonne TranJun NodaZabit HameedJun RenZacharias A. ViskadourakisJun ShibayamaZachary BallardJun SuzurikawaZachary H. LevineJun TakamatsuZafar BalochJun WangZahir HussainJun YangZahir M. HussainJun YuanZahra AbbasiJun ZhengZahra KazemiJun ZhuZahra PooranianJunaid ShujaZaid AlyasseriJunbang WangZaidao WenJunbo SunZaifeng ShiJuncai XuZain Ul-AbdinJung Hwan ParkZain-Aldeen A. RahmanJung Jin KimZakaria MelianiJung Min PakZakariya OraibiJung Sik JeongZaloa Sanchez VarelaJung Tae (Steve) KimZan LiJung Woo SohnZaneta Swiderska-ChadajJung-Ah ShinZao YiJungchan ChoZaojun FangJung-Chih ChenZara BagdasarianJunghee HanZavala-Rivera PaulJung-hee KimZbignevs MarcinkevicsJung-hwan HwangZbigniew AdamiakJungil ChoiZbigniew DziongJungkwun KimZbigniew GomolkaJungmyung LeeZbigniew J. SrokaJungrack KimZbigniew KrzemianowskiJung-Ryun LeeZbigniew KuleszaJungsu ChoiZbigniew MarszalekJungwon HuhZbigniew NadolnyJung-Yeol YeomZbigniew OtrembaJun-Ho HuhZbigniew UsarekJunho JeongZbigniew ZembatyJunho YeoZbisław TaborJunhong ParkZbyněk RaidaJun-Hong ShenZdenek MartinasekJunhu RuanZdenek SlaninaJunhui HuZdzisław GosiewskiJunhui LiZe JiJun-Hwan ShinZeel MaheshwariJunichi InoueZeev Vladimir VolkovichJunichi NaganawaZeev VolkovichJunjie LinZehan YaoJunjing DengZekra MousaviJunjue WangZelei ChengJunko KaneroŽeljka CvejićJunkyeong KimZeljko HocenskiJunli JinŽeljko ŠpoljarićJunliang JinZeljko ZilicJunqi GaoZengsheng ChenJunqi WangZeno GeradtsJun-Rui LiangZenon LukaszewskiJunseok KwonZenon NieckarzJun-seok ParkZenonas TurskisJunseop LeeZesong FeiJunsheng LiZeyang ZhouJunsheng RenZhan GaoJunsheng WangZhan SongJuntong XiZhan WangJunwei CaoZhangjie LiuJun-Won HoZhangjun YuJunxiang LianZhangyong LiJunxiang TanZhanhui WangJunxiang ZhuZhanjun WuJunyi CaoZhanlin JiJunzhe ZhangZhanna A. BoevaJunzheng JiangZhanping SongJuraj BartolicZhan-Yi HuJuraj DianZhao Cheng HeJuraj GašinecZhao RenJuraj GazdaZhao XuJuraj GerliciZhaogang ShuJuraj KacurZhaohui LiJuraj MachajZhaojie LuoJuraj PetrovićZhaoqian XieJuraj SinayZhaoqiang ChuJūratė Sužiedelytė-VisockienėZhaowei KongJürgen FreudenbergerZhaowei ZhangJürgen HartmannZhaowen YanJürgen SachsZhaoyang DingJurgita AntuchevicieneZhaoye QinJurgita MalaiškienėZhaozheng HuJuri TaborriZhaozong MengJuris BurlakovsZhe ChenJustin AlbertZhe ShenJustin ChapmanZhe SunJustin F. SchneidermanZheguang ZouJustin VandenbrouckeZhen QiuJustina SestokeZhen WangJustus MarquetandZhen WuJustyna BohaczZhen XuJustyna KozlowskaZhen ZhangJustyna LiberaZhenbin ZhangJustyna Patalas-MaliszewskaZhenfeng GongJustyna SulejZhenfeng ShaoJustyna TomaszewskaZheng BinJyh-Miin LinZheng CuiK. Abhimanyu Kumar PatroZheng (Eddie) LiKa KimZheng FanKaan AkşitZheng JiKaare MikkelsenZheng KuangyuKa-Chun (Joseph) SiuZheng LiKagan KermanZheng QianKah Hou TengZheng TongKai ChenZheng WangKai ChengZhengfang WangKai DingZhenggan ZhouKai DongZhengkuan LeeKai HuangZhengliang ZhuKai LiuZhenglin LiKai MichelsZhengqiang WangKai ShenZhenguang LiuKai WangZhengwei LiKai WuZhengxiang ShiKai XiongZhengxing SunKai XuZhenhu JinKai ZhouZhenhua YuKaichen SongZhenhua ZouKaichen XuZhenhuan YiKai-Chun LiuZhenhui DuKaifeng ZhangZhenkun LeiKaige WuZhenling Ma‪Kai-Jun PaiZhenlong XuKaikai XuZhenming PengKailun YangZhenping GuanKaimeng DingZhenxiang YiKaimin SunZhenxin WangKaiqing ZhouZhenxin ZhangKais GadhoumiZhenxing GaoKaitao LiZhenyang DingKaixuan ChenZhenyin HaiKaixuan ZhaoZhenyu LiKaiyuan ShiZhenzhan WangKalathur SanthanamZhenzhi LinKalayci TunaZhenzhong HuKaleeswaran BalasubramaniamZhenzhou WangKalevi KilkkiZheyuan ChenKalle TammemaeZhezhuang XuKaloyan YovchevZhi NingKamal ChapagainZhibin SunKambiz TehraniZhibin WangKamel BelloulataZhicheng CuiKamil DimililerZhicheng QiuKamil GareevZhidong XueKamil KowalczykZhifang LiangKamil KrasuskiZhifang PanKamil KrzywinskiZhifeng TangKamil MakiełaZhifu LiuKamil StaniecZhigang LiKamila HauleZhiguang TangKamila ŘasováZhiguo ZhangKamran AvanakiZhihai QiuKamran IqbalZhihong DengKamran JavedZhihong YaoKamran ShaukatZhihua XiongKamrul IslamZhihua XuKan ShojiZhihua YuKan WangZhihui ZengKang AnZhihuo XuKang DingZhiJun YanKang Hao CheongZhikang LiKang ShiZhiling HouKang SongZhilu LaiKang-Chun PengZhimeng YinKanghyeok YangZhiming ZhangKangjae LeeZhiping LiuKangli LiuZhiqian ChenKang-min LeeZhiqiang HuKang-Ming ChangZhiqiang LiangKanika BansalZhiqiang XuKanike Raghavendra KumarZhiqin ZhuKanji OnoZhiqun HuKaori FujinamiZhisheng GaoKaoru IchikawaZhitao XiaoKapil GulatiZhitian LiuKara GavinZhiwei HeKarcius Day Rosário AssisZhiwei MaoKarekin EsmeryanZhiwei ZhaoKarel CharvatZhixi NieKarel MlsZhiyi TangKarel PavelkaZhiyi WuKarel RoubikZhiyong LiuKaren GordonZhiyong ZhangKari KopraZhiyong ZhouKari LappalainenZhiyu JiangKari V. VienolaZhiyuan FengKarim AlyZhiyuan RenKarin Anna HummelZhiyuan YuKarin ConinxZhiyuan ZhuKarina FigueroaZhizhong DingKarina LebelZhong SuKarl AnderssonZhongbao WeiKarl Dietrich Von EllenriederZhongfeng WangKarla MerazzoZhonghao LiKarla MossiZhonghua XueKarla Munoz EsquivelZhongli WangKarlo GriparićZhongmin JinKarna BryanZhongmin QiuKarol AbratkiewiczZhongmin XiaoKarol KrólZhongqi LiuKarol MiądlickiZhongxiang WeiKarol WinkelmannZhongyi HuKarolina KudelinaZhou XuKashif SaleemZhou ZhangKashif ZiaZhoutao ZhengKasim KorkmazZhouyi WangKaspar LasnZhu SunKaspar M.B. JansenZhu XiaoKasper Van GasseZhuang ChengKasper Van WijkZhuangqiang GaoKasun Pradeepa WijayaratnaZhuangzhi SunKatarina ItrićZhuhua HuKatarína PukanskáZhuofu LiuKatarzyna AntoszZhuoqi ChengKatarzyna BialasZia UllahKatarzyna BoratyńskaZicheng ChiKatarzyna HarezlakZichun ZhongKatarzyna Jochymczyk-WoźniakŽiga GosarKatarzyna KopczewskaZigang DengKatarzyna KurzatkowskaZihan KanKatarzyna Marciniak-ŁukasiakZijia LiKatarzyna Nowakowska-LipiecZijia ZhongKatarzyna PentośZijian QiaoKatarzyna Tyszczuk-RotkoZijian WangKatarzyna Zabielska-AdamskaZijie ZhuKatarzyna ZawadaZijing WongKaterina D. TzimourtaZijun WangKaterina KabassiZilong ZhangKaterina LazarovaZilu LiangKaterina TzafilkouZimi SawachaKaterine RomeoZinaida M. KuznetsovaKateryna SergieievaZine El Abiddine FellahKatharina RombachZipei FanKatherine KirkZiping LiuKathiravan SrinivasanZitong YuKathleen A. R. SchildrothZiv YanivKathryn CrossŽivana Ninčević-GladanKathryn L. HavensZiyue ZengKathyayini SivasubramanianZlatin ZlatevKati LaaksoZofia RzepeckaKatia PaneZoheir KhademianKatina KralevskaZois BoukouvalasKatja KousZoka MilanKatrin SchmittZöldy MátéKatsunori OyamaZoltán BálintKavita SharmaZoltán GillayKay-Pong Daniel YipZoltan JuhaszKazimierz BecekZoltán KolláthKazimierz FabisiakZoltan RozsaKazimierz GutZongjie WangKazimierz WilkoszZongmei GaoKazuhiko KawamotoZongsong GanKazuhiro ArugaZongyin YangKazuhiro P. IzawaZoran MinicKazuki IkedaZoran PerićKazuo OnoZoran SalcicKazuo YonekuraZoran StamenkovicKazuyuki NakahataZouhair HananiKC SantoshZsolt GulácsiKe BiZsolt MolnárKe ChenZuguang YingKe FengZuojun LiuKe GuanZuo-Min TsaiKe ShiZuomin ZhaoKe SiZuqing ZhuKe TianZvi RothKe WangZvonko RadosavljevicKe XuZygmunt MiknoKe Zhang



